# Supplementary figures and images for: Dual-specific autophosphorylation of kinase IKK2 enables phosphorylation of substrate IκBα through a phosphoenzyme intermediate (part 1 of 2)
Source: eLife. 2025 Jun 30;13:RP98009. doi: 10.7554/eLife.98009 (PMC12208667; doi:10.7554/eLife.98009)

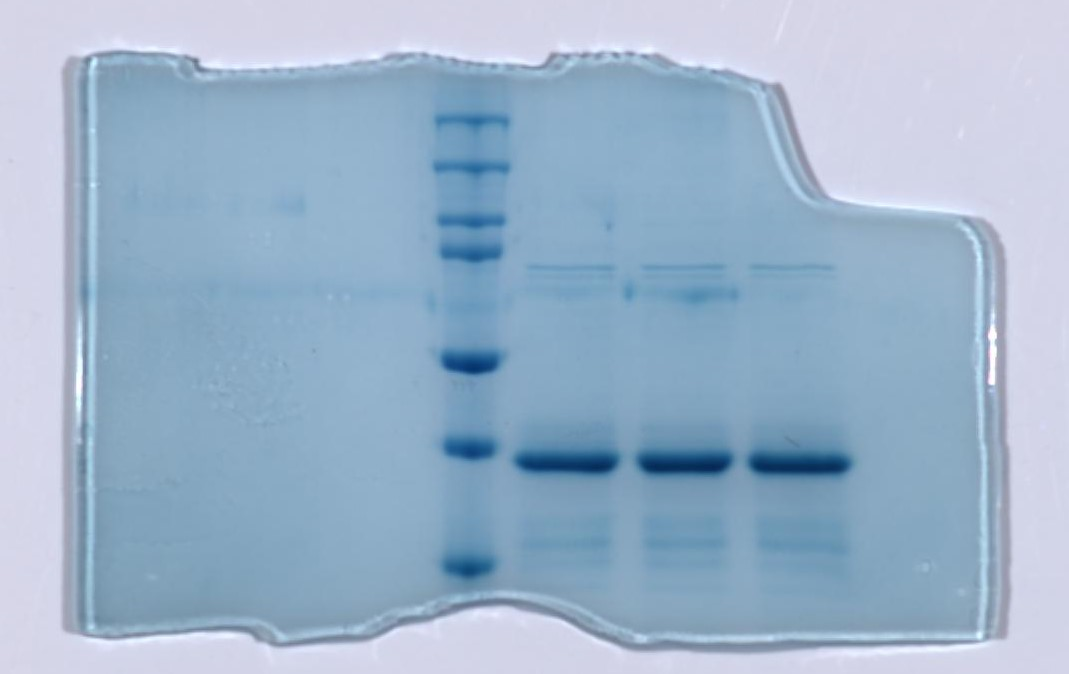

Supplement: Figure 1—source data 1. [file elife-98009-fig1-data1.zip › Figure 1-source data 1/Fig1E-coomassie.tif]

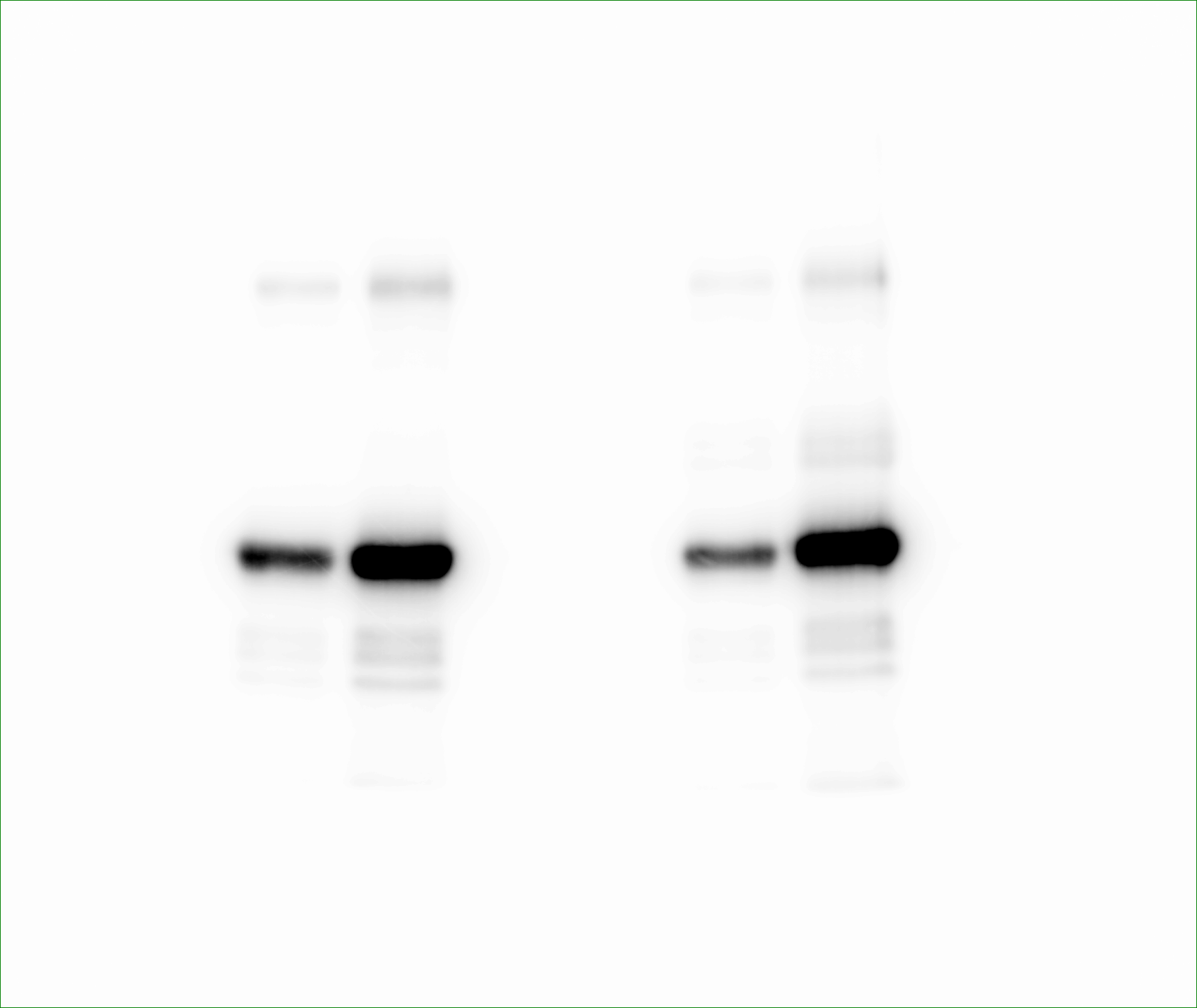

Supplement: Figure 1—source data 1. [file elife-98009-fig1-data1.zip › Figure 1-source data 1/Fig1D_right-autorad.tif]

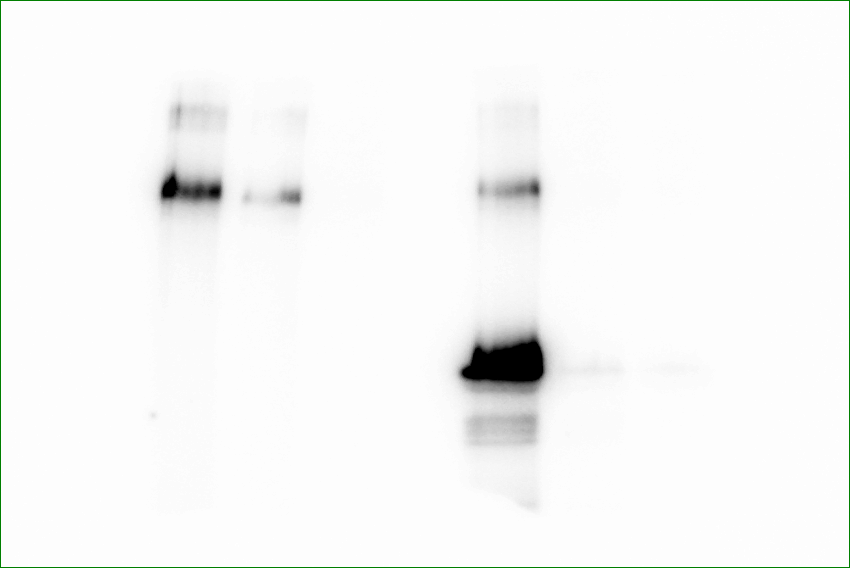

Supplement: Figure 1—source data 1. [file elife-98009-fig1-data1.zip › Figure 1-source data 1/Fig1E-autorad.tif]

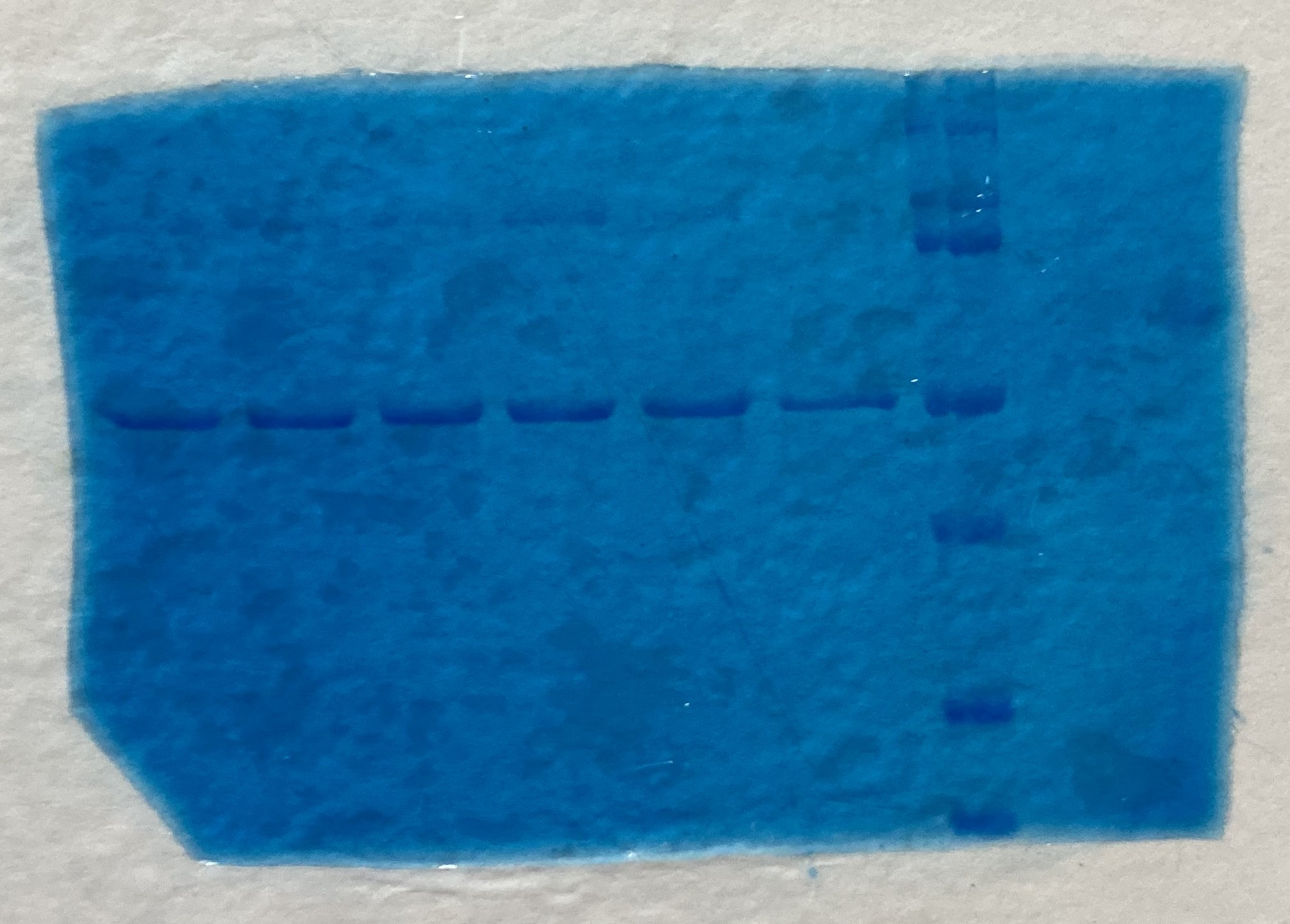

Supplement: Figure 1—source data 1. [file elife-98009-fig1-data1.zip › Figure 1-source data 1/Fig1D_left-coomassie.tif]

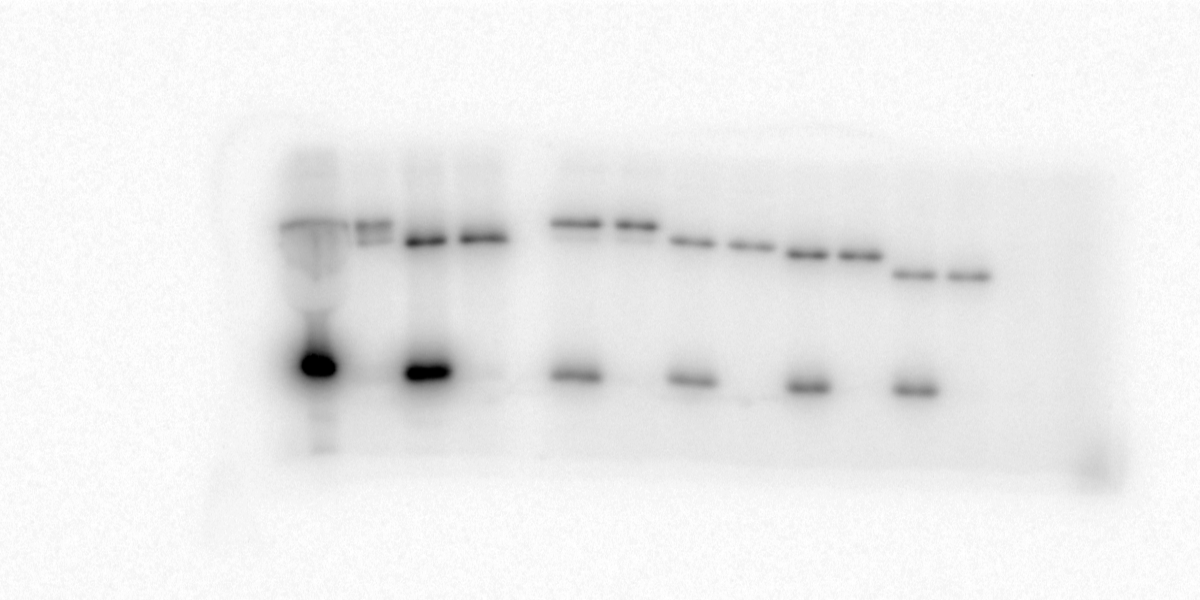

Supplement: Figure 1—source data 1. [file elife-98009-fig1-data1.zip › Figure 1-source data 1/Fig1F-autorad.tif]

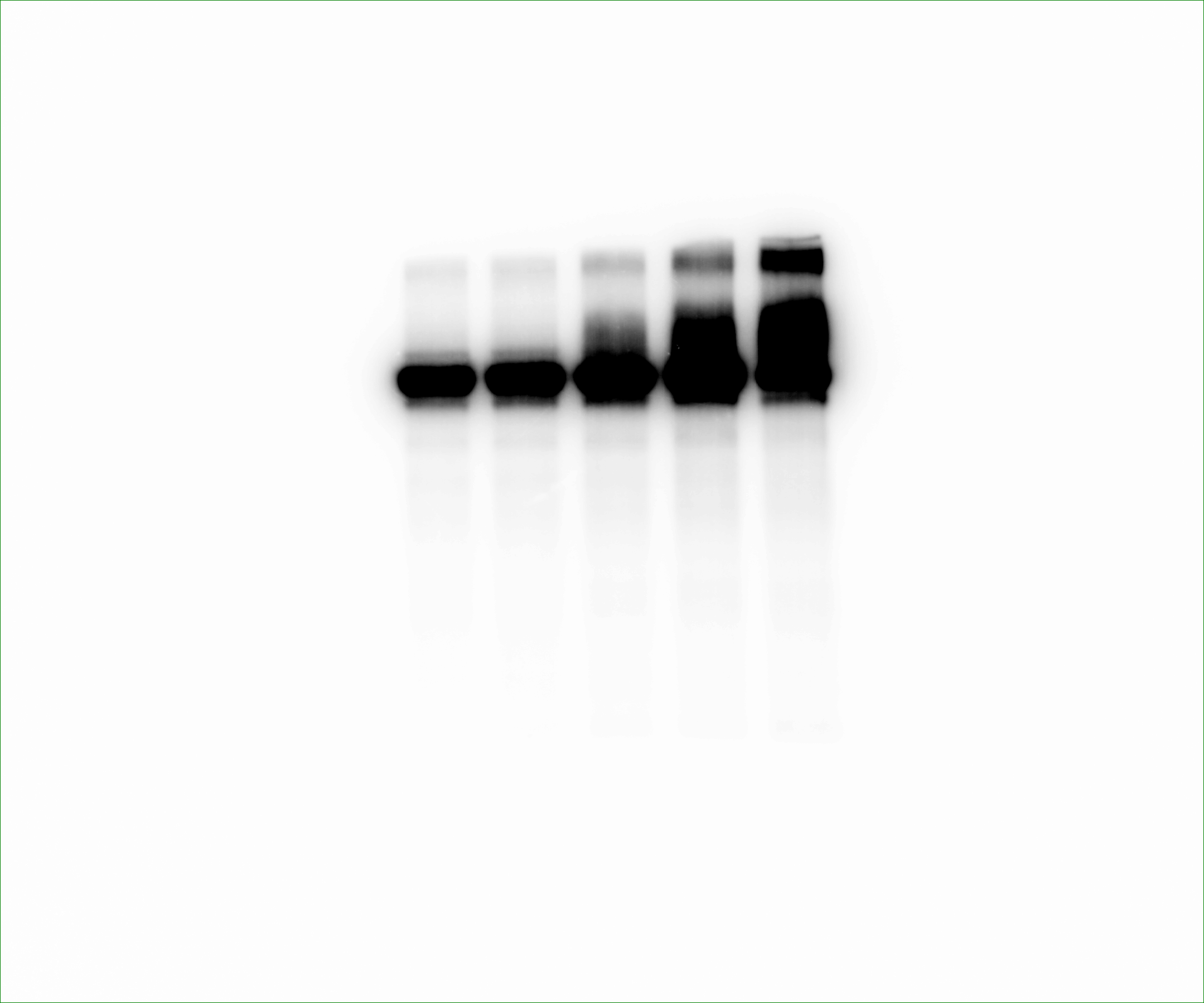

Supplement: Figure 1—source data 1. [file elife-98009-fig1-data1.zip › Figure 1-source data 1/Fig1C-autorad.tif]

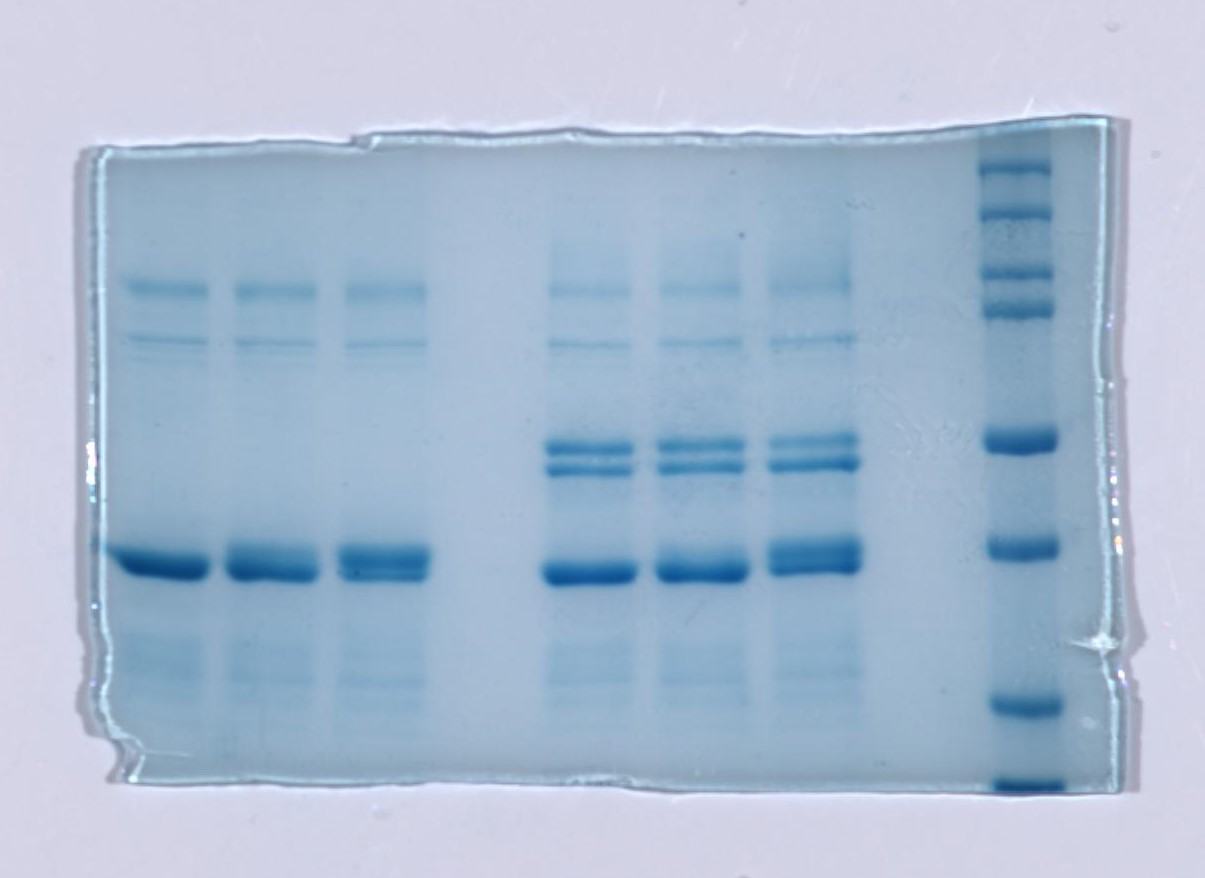

Supplement: Figure 1—source data 1. [file elife-98009-fig1-data1.zip › Figure 1-source data 1/Fig1D_right-coomassie.tif]

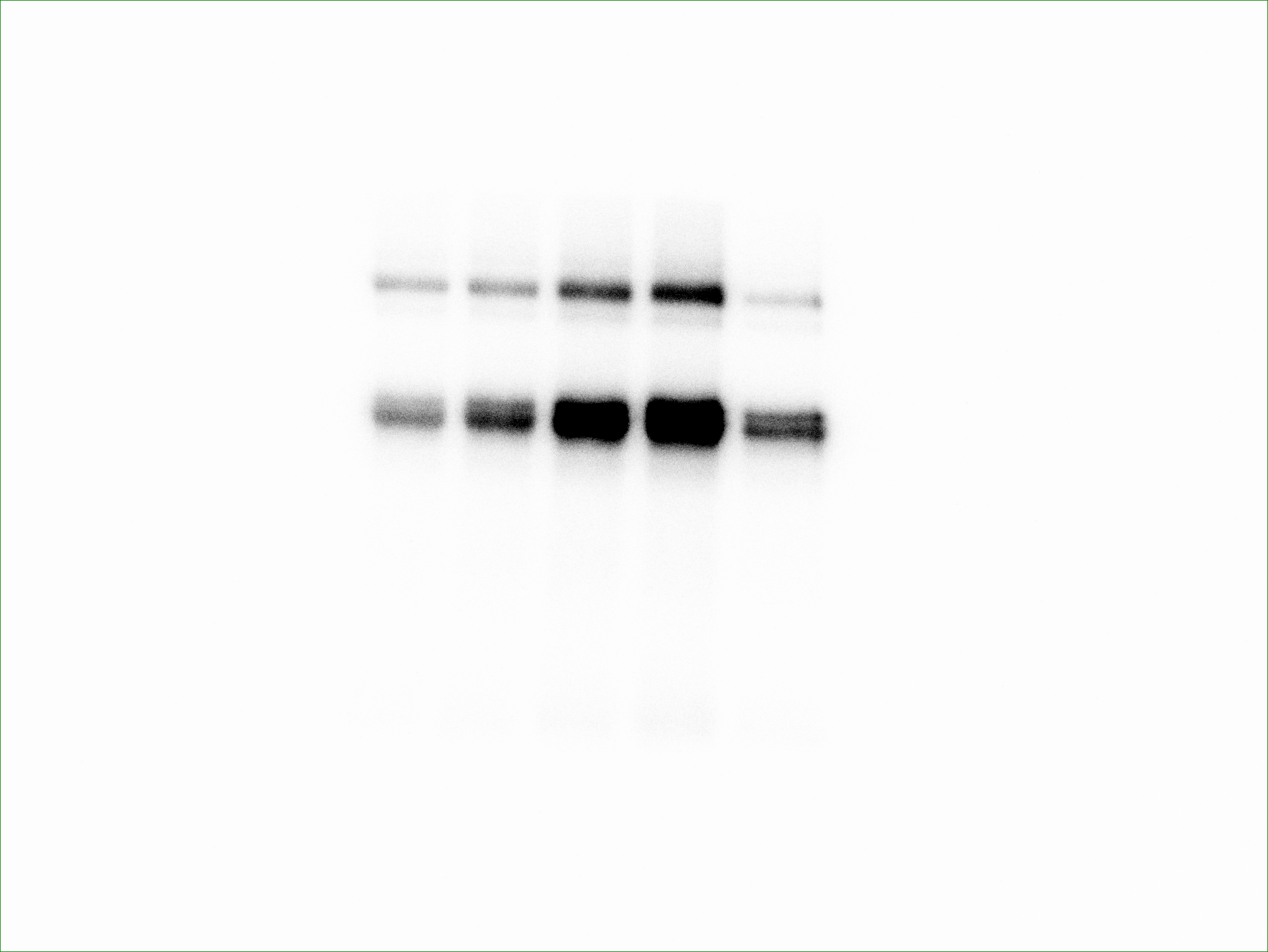

Supplement: Figure 1—source data 1. [file elife-98009-fig1-data1.zip › Figure 1-source data 1/Fig1D_left.tif]

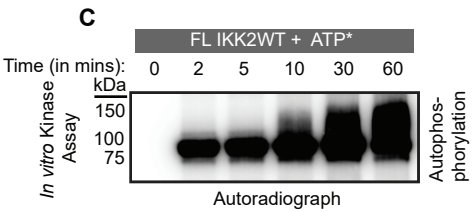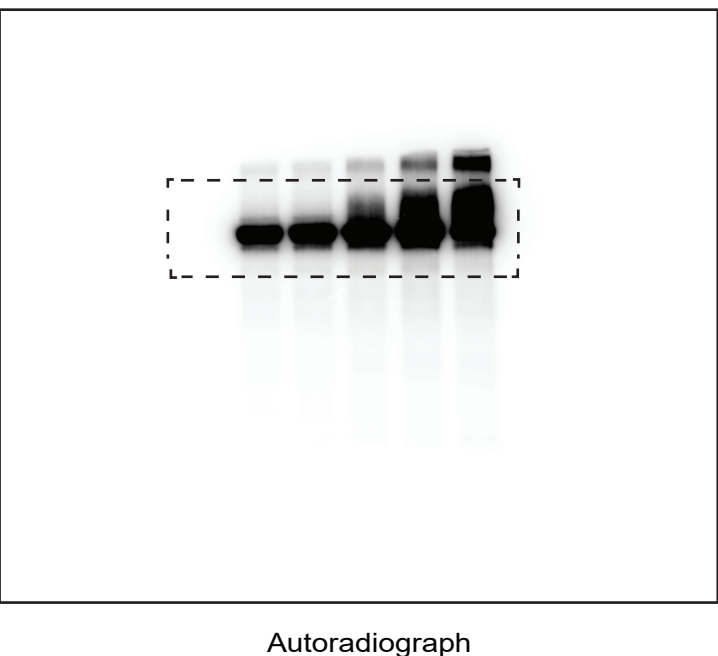

Supplement: Figure 1—source data 2. [file elife-98009-fig1-data2.zip › Figure 1-source data 2/Fig1C.pdf]

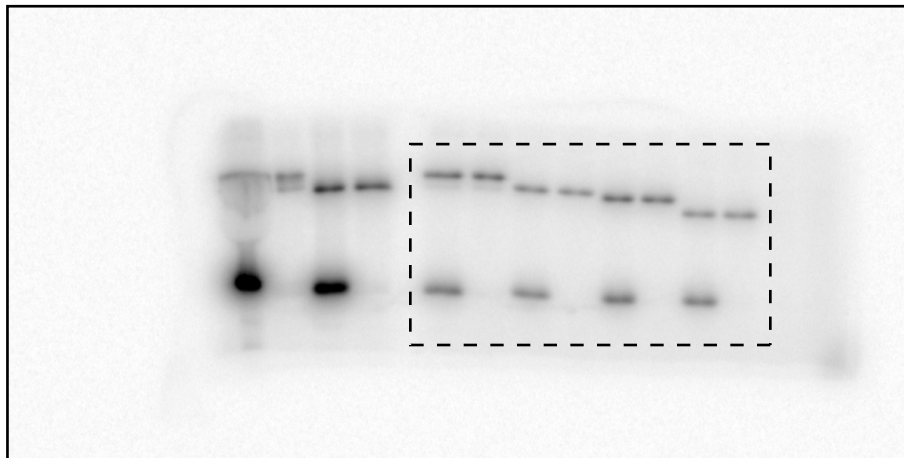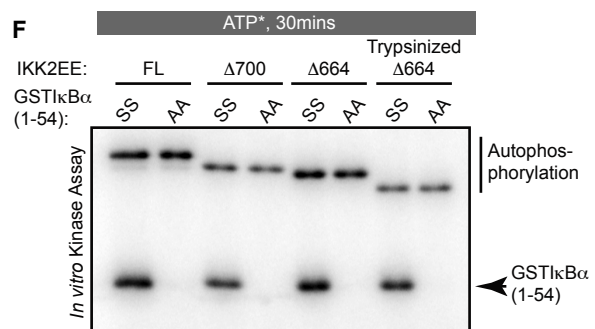

Supplement: Figure 1—source data 2. [file elife-98009-fig1-data2.zip › Figure 1-source data 2/Fig1F.pdf]

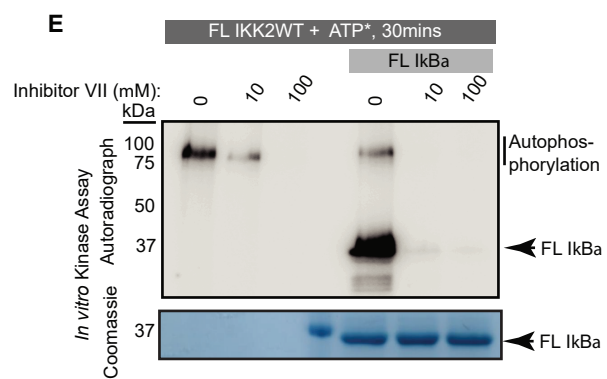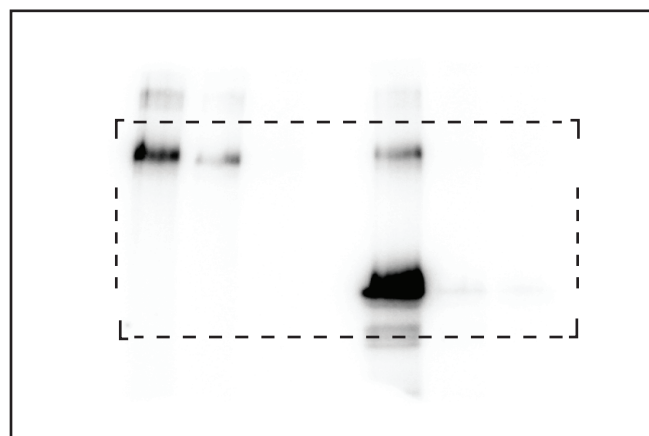

Autoradiograph

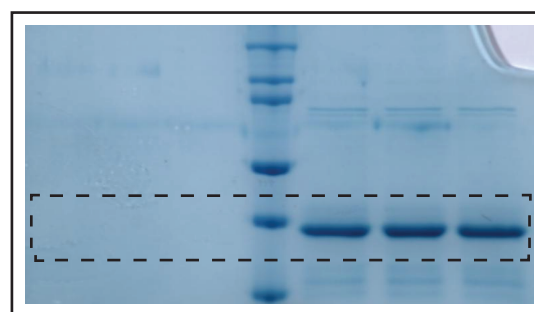

Coomassie

Supplement: Figure 1—source data 2. [file elife-98009-fig1-data2.zip › Figure 1-source data 2/Fig1E.pdf]

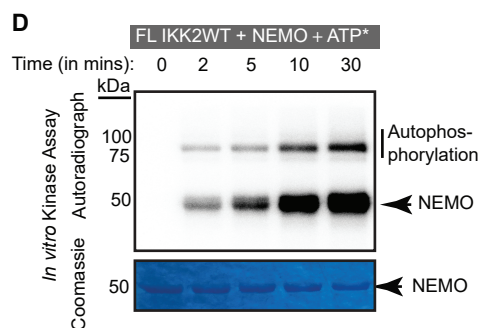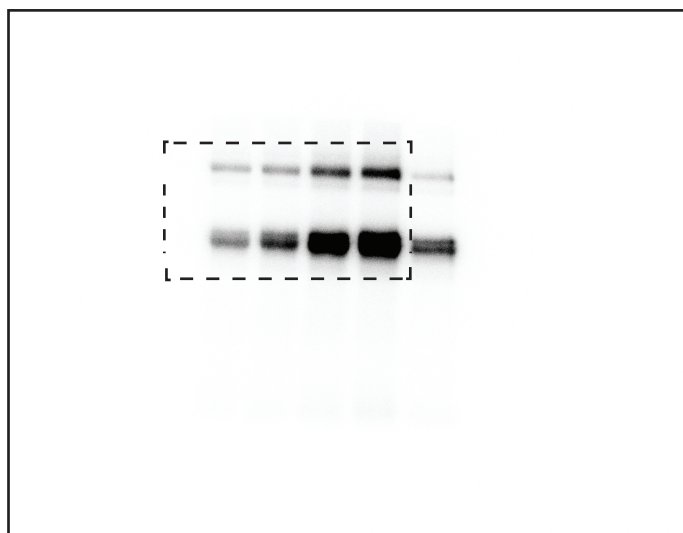

Autoradiograph

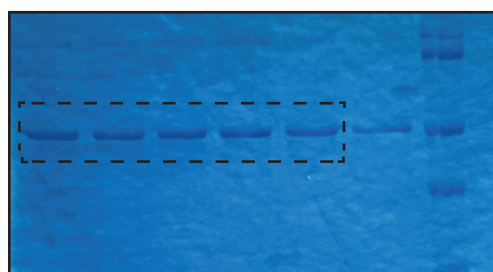

Coomassie

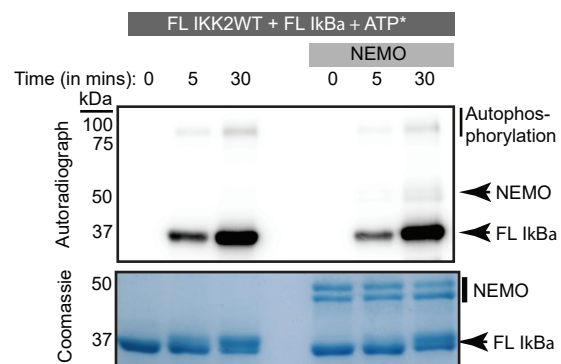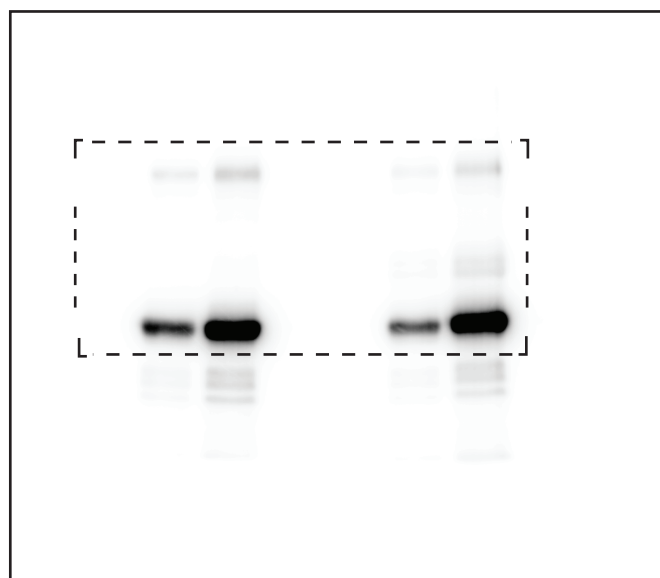

Autoradiograph

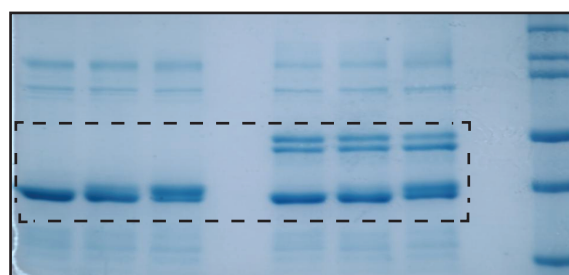

Coomassie

Supplement: Figure 1—source data 2. [file elife-98009-fig1-data2.zip › Figure 1-source data 2/Fig1D.pdf]

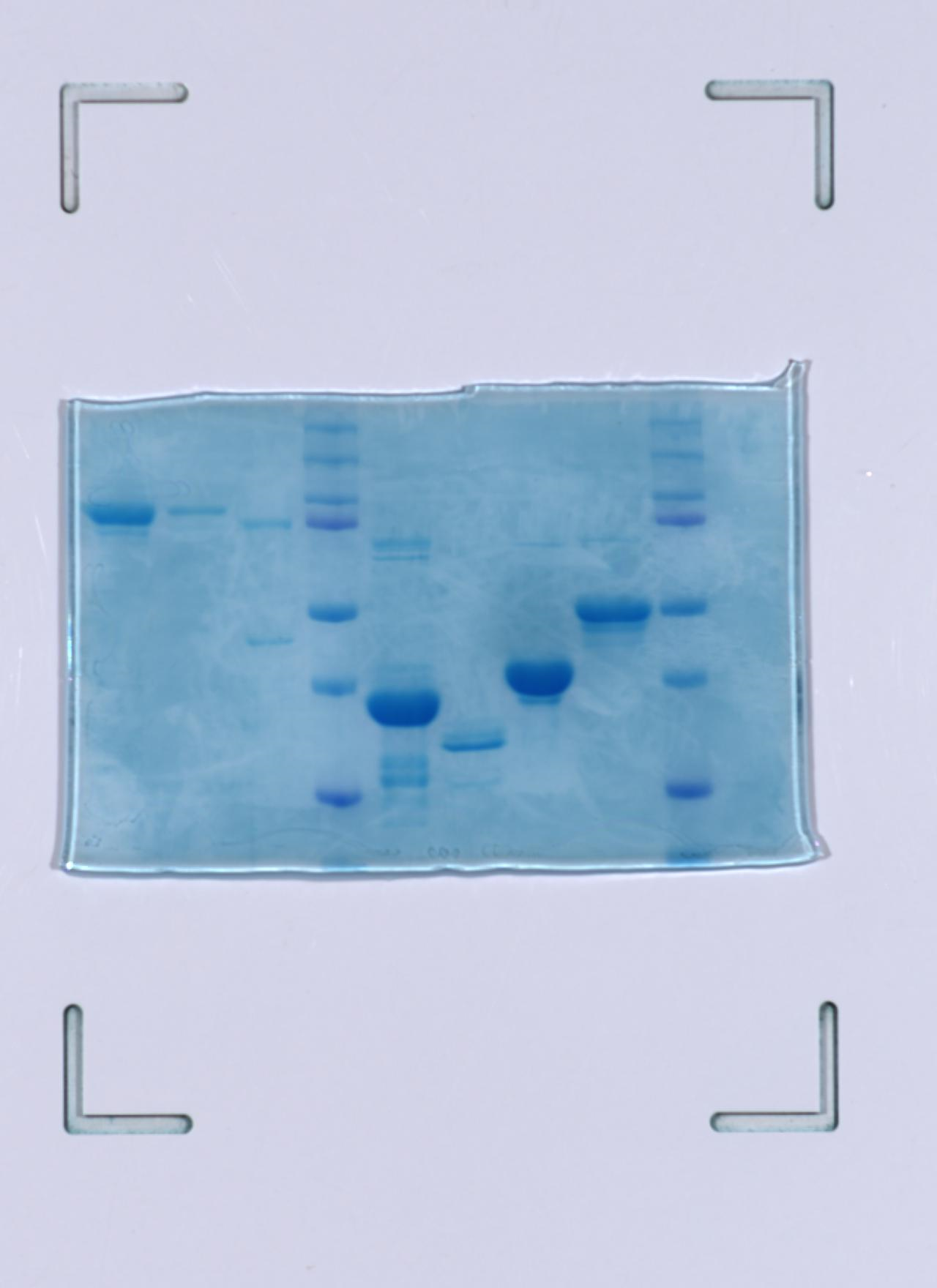

Supplement: Figure 1—figure supplement 1—source data 1. [file elife-98009-fig1-figsupp1-data1.zip › Figure 1-figure supplement 1-source data 1/Fig1-fig supp 1C-coomassie.tif]

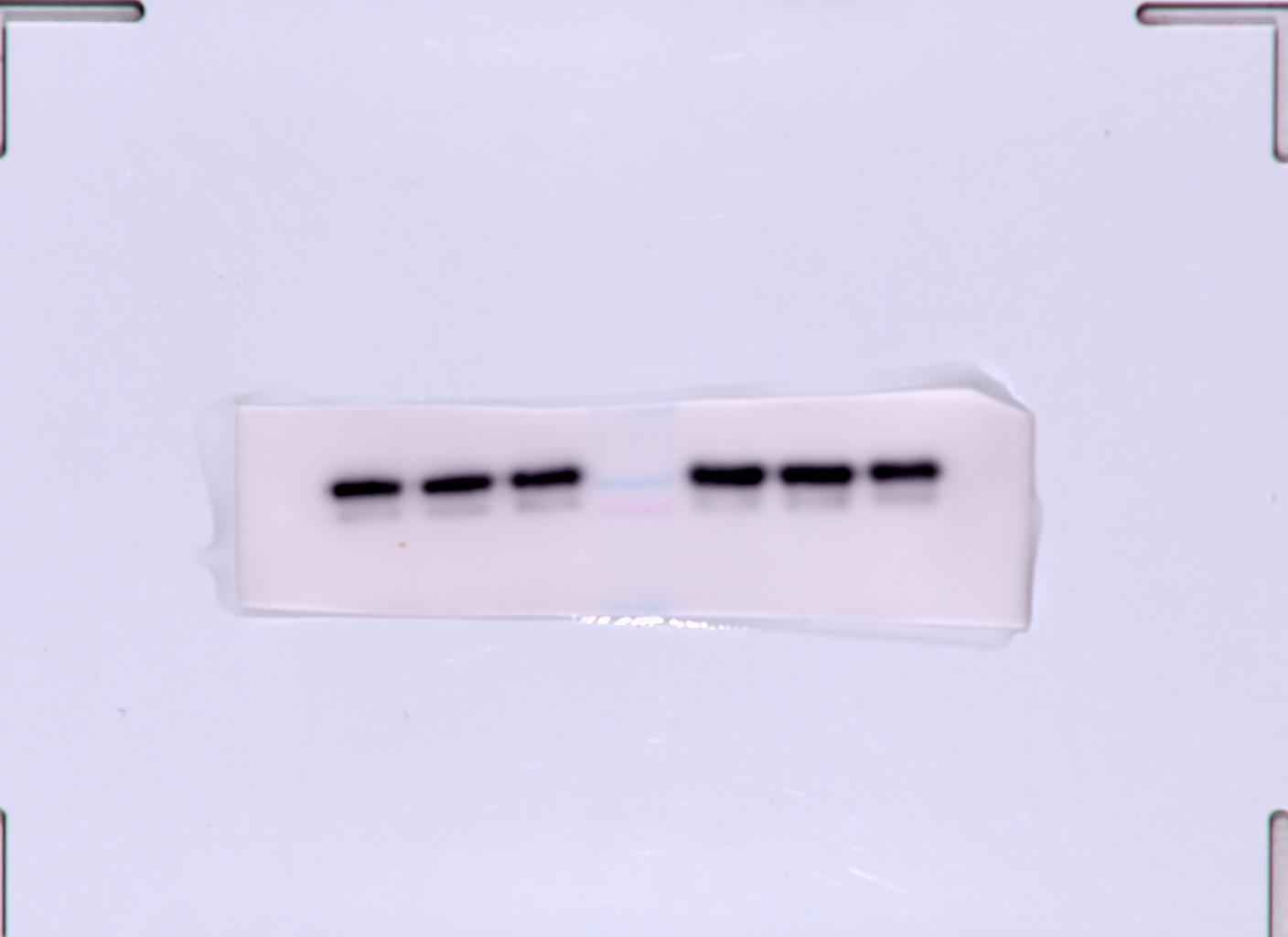

Supplement: Figure 1—figure supplement 1—source data 1. [file elife-98009-fig1-figsupp1-data1.zip › Figure 1-figure supplement 1-source data 1/Fig1-fig supp 1B-IKK2.tif]

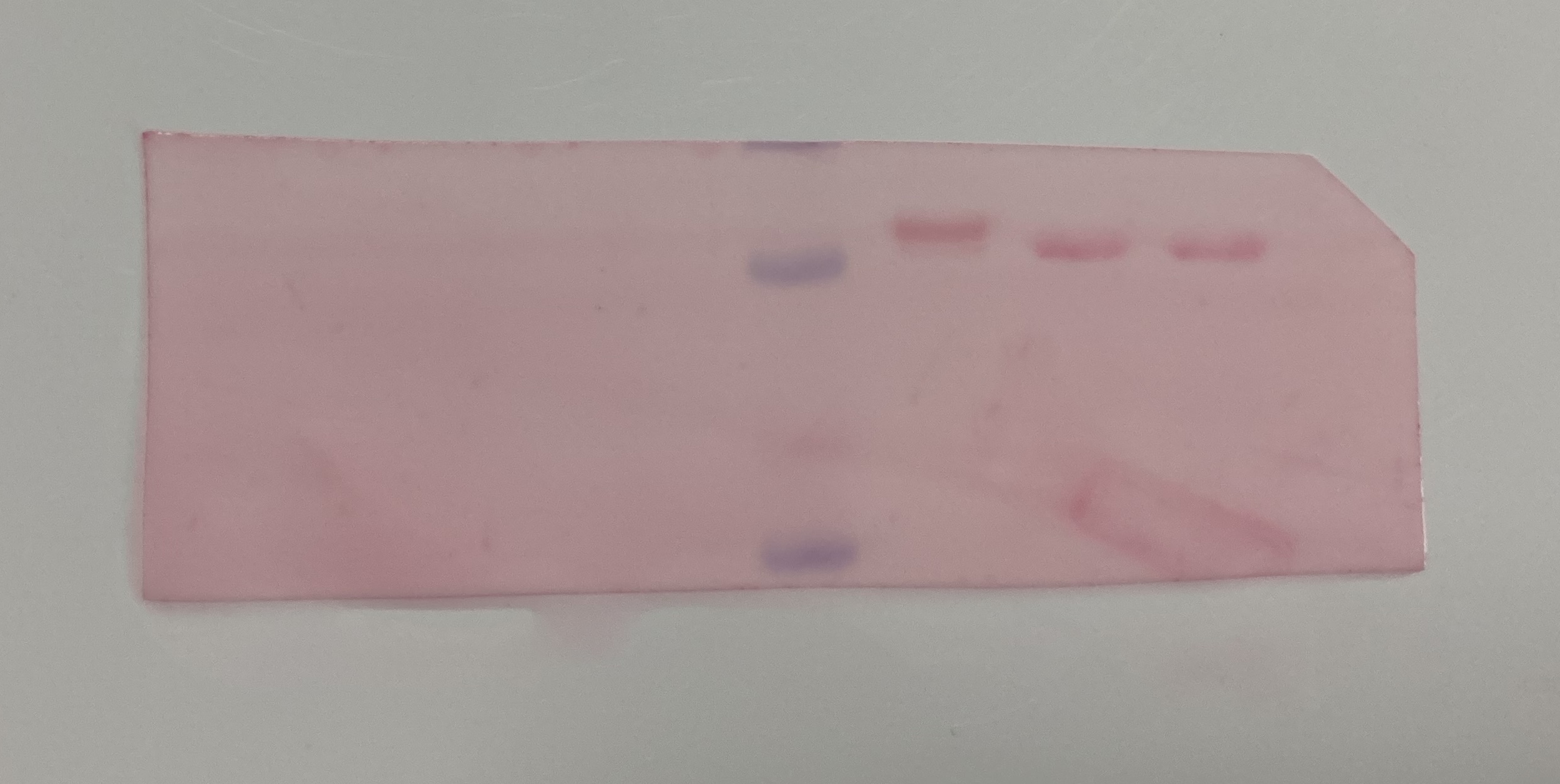

Supplement: Figure 1—figure supplement 1—source data 1. [file elife-98009-fig1-figsupp1-data1.zip › Figure 1-figure supplement 1-source data 1/Fig1-fig supp 1B-Ponceau.tif]

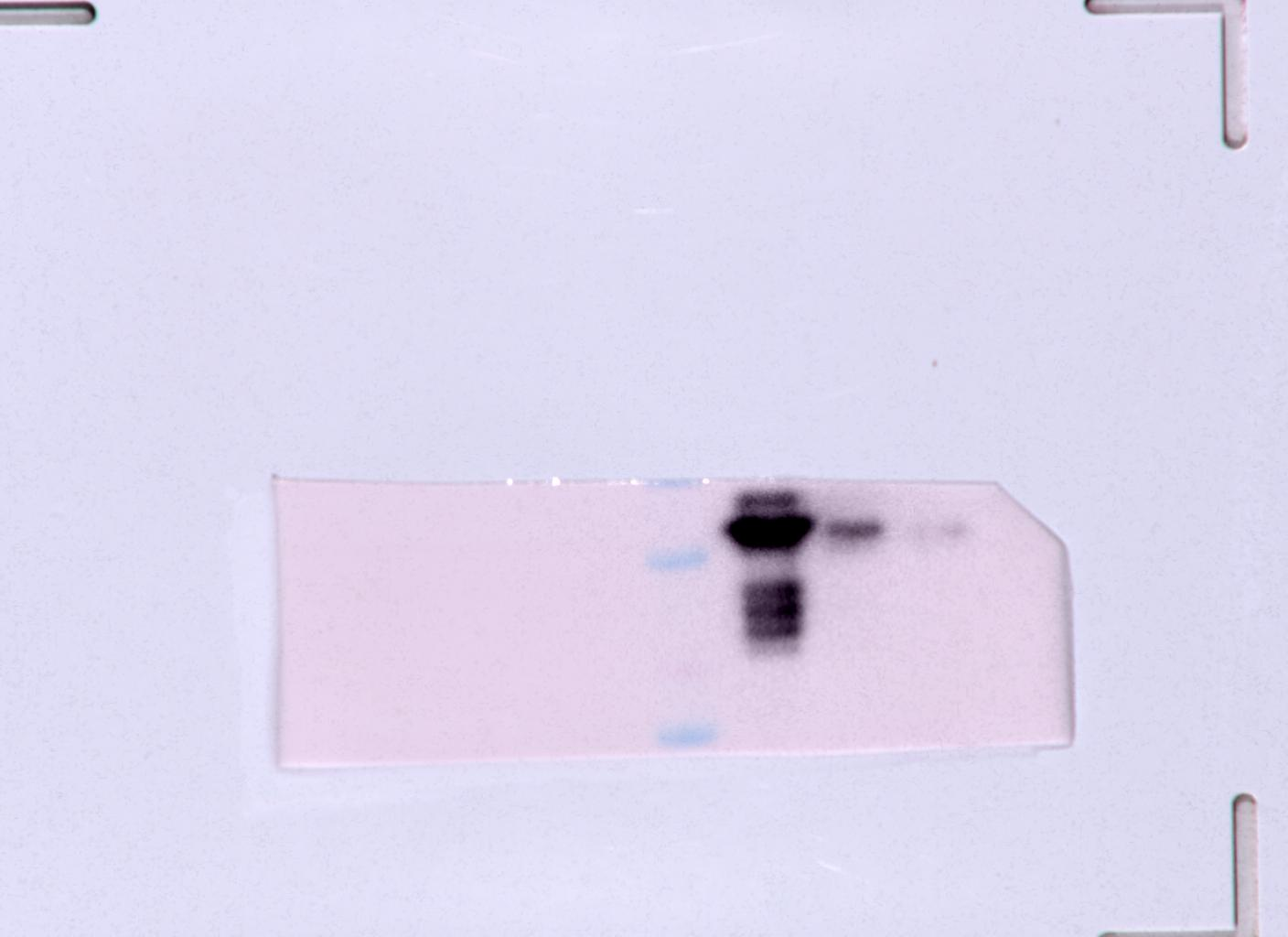

Supplement: Figure 1—figure supplement 1—source data 1. [file elife-98009-fig1-figsupp1-data1.zip › Figure 1-figure supplement 1-source data 1/Fig1-fig supp 1B-pIkBa.tif]

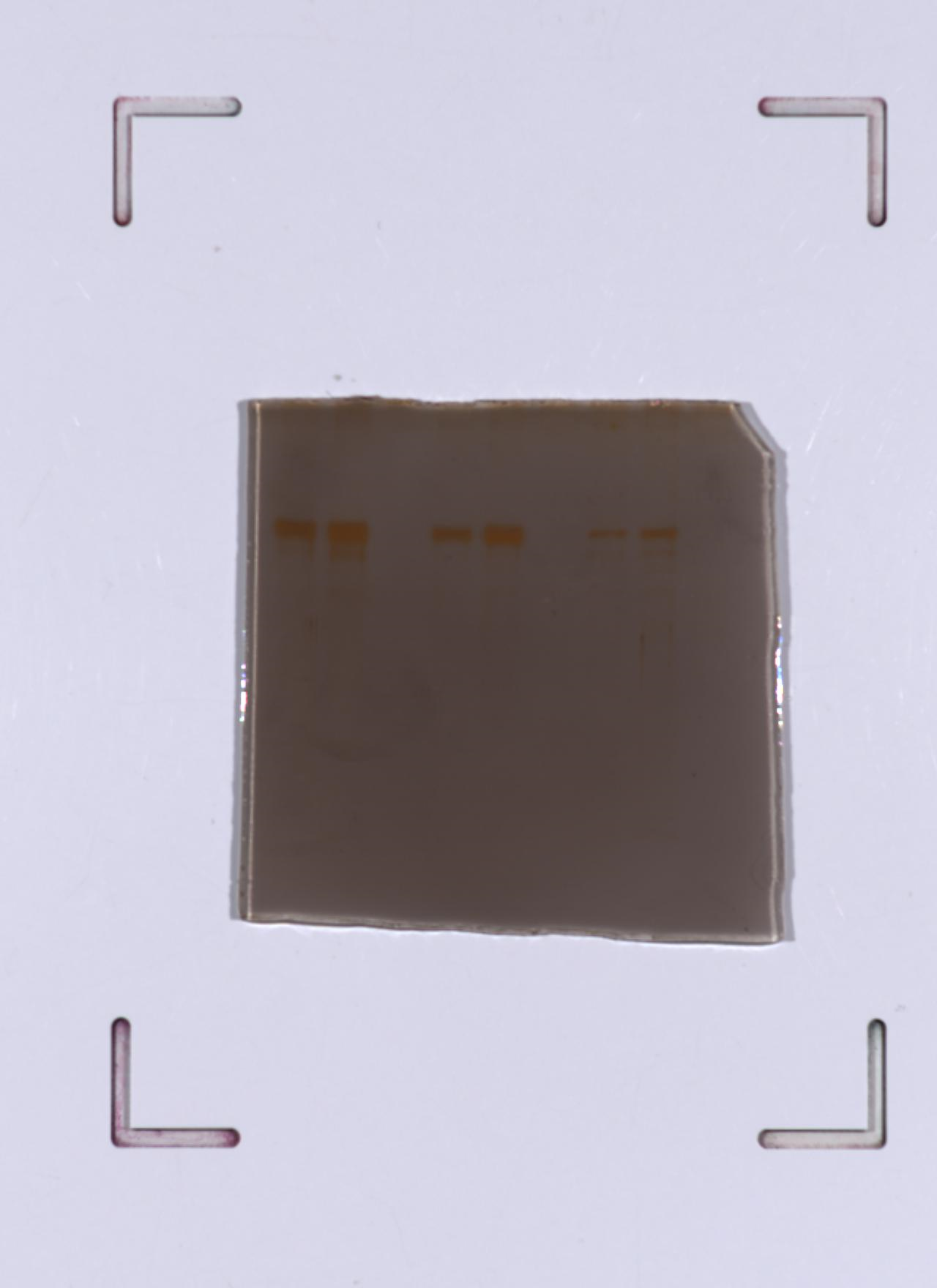

Supplement: Figure 1—figure supplement 1—source data 1. [file elife-98009-fig1-figsupp1-data1.zip › Figure 1-figure supplement 1-source data 1/Fig1-fig supp 1C-silver staining.tif]

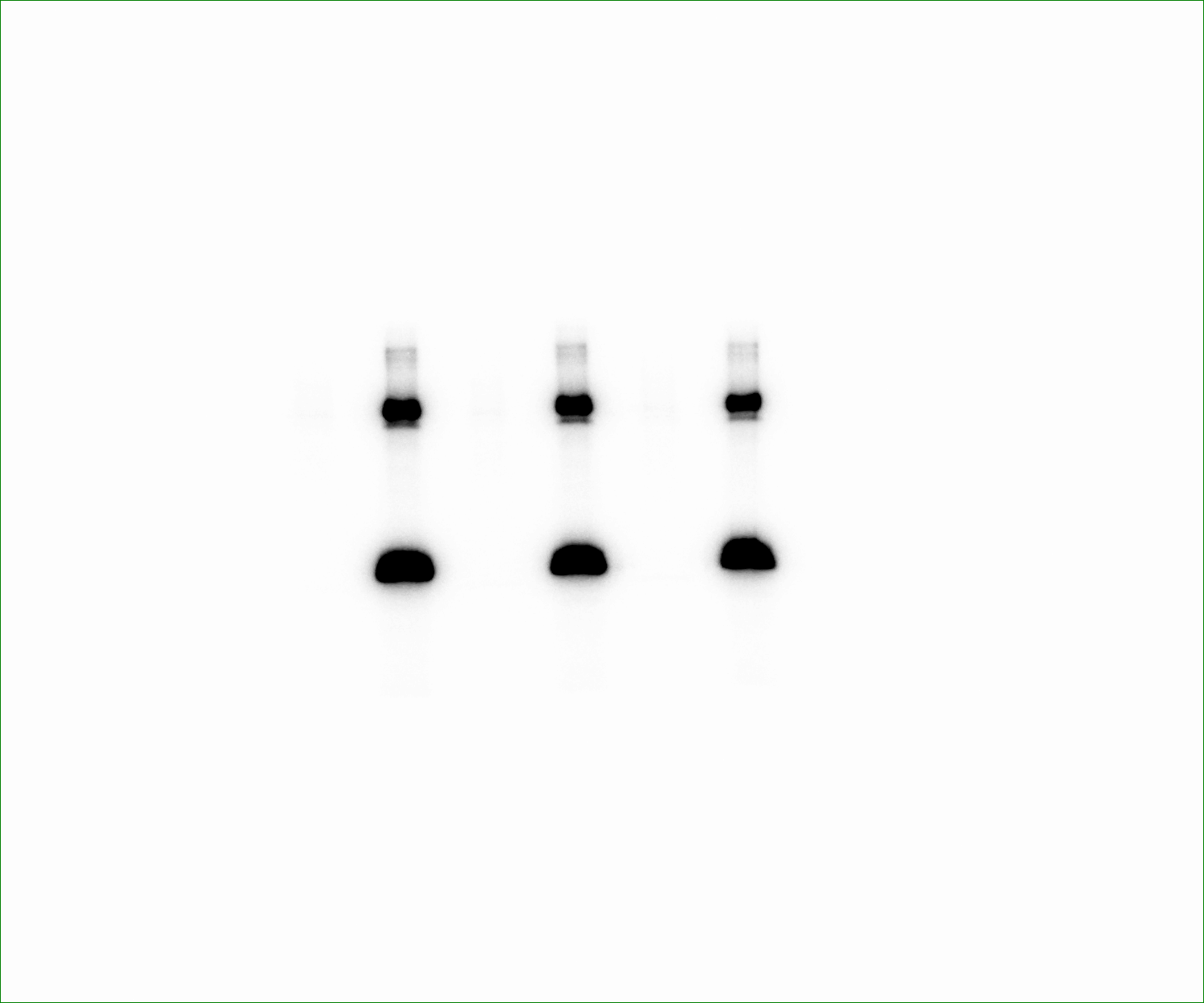

Supplement: Figure 1—figure supplement 1—source data 1. [file elife-98009-fig1-figsupp1-data1.zip › Figure 1-figure supplement 1-source data 1/Fig1-fig supp 1A-autoradiograph.tif]

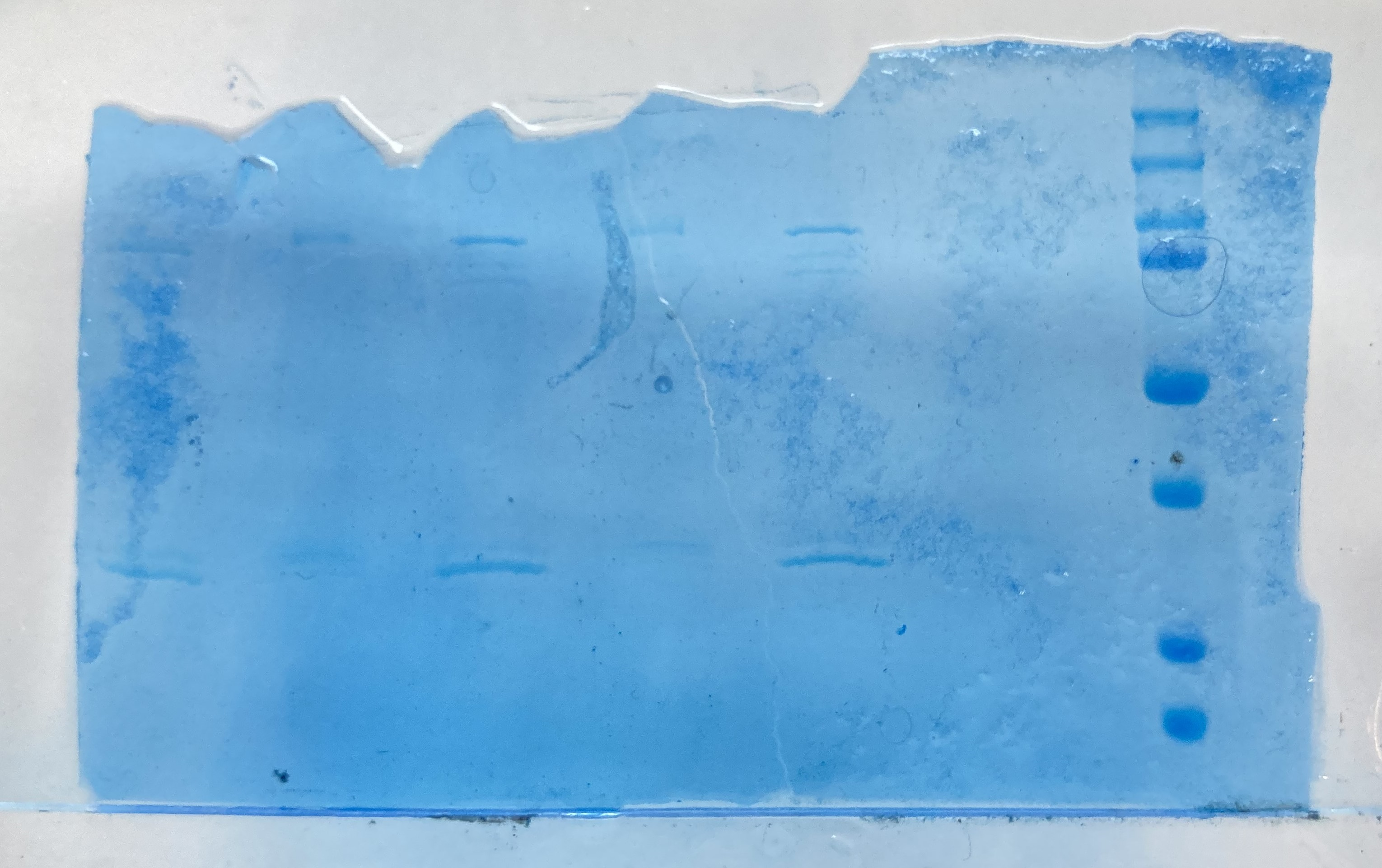

Supplement: Figure 1—figure supplement 1—source data 1. [file elife-98009-fig1-figsupp1-data1.zip › Figure 1-figure supplement 1-source data 1/Fig1-fig supp 1A-coomassie.tif]

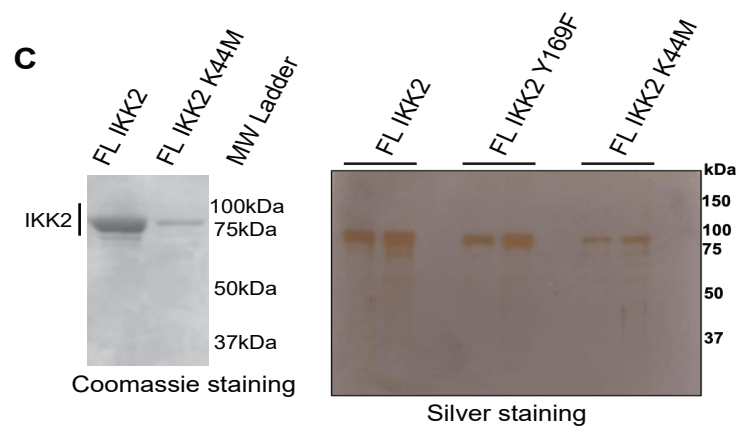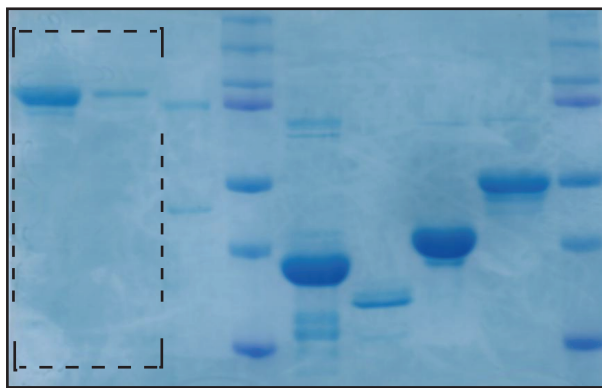

Coomassie staining

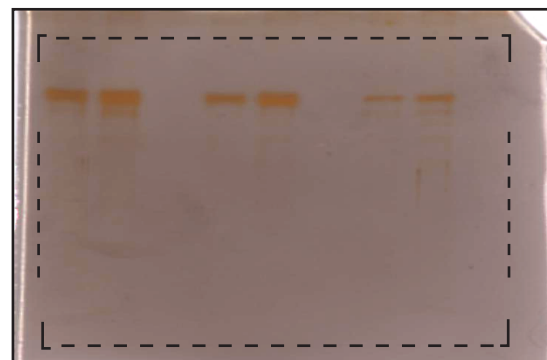

Silver staining

Supplement: Figure 1—figure supplement 1—source data 2. [file elife-98009-fig1-figsupp1-data2.zip › Figure 1-figure supplement 1-source data 2/Fig1-figure supplement 1C.pdf]

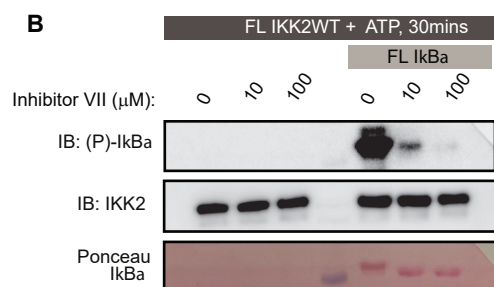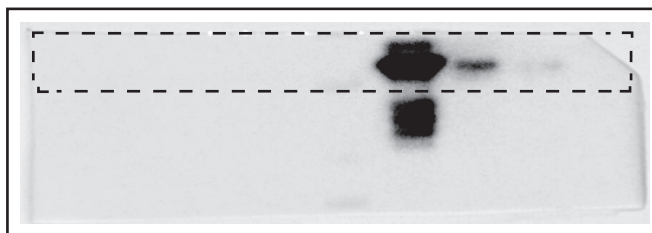

IB: (P)-IκBa

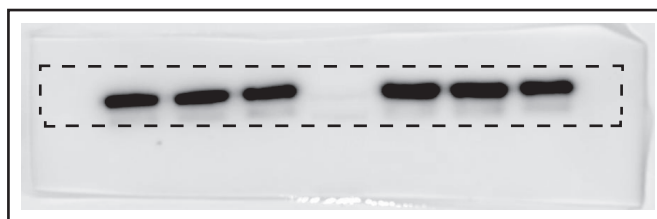

IB: IKK2

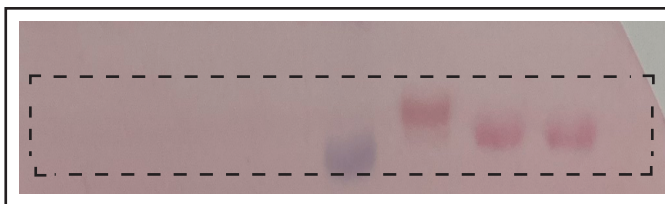

Ponceau IκBa

Supplement: Figure 1—figure supplement 1—source data 2. [file elife-98009-fig1-figsupp1-data2.zip › Figure 1-figure supplement 1-source data 2/Fig1-figure supplement 1B.pdf]

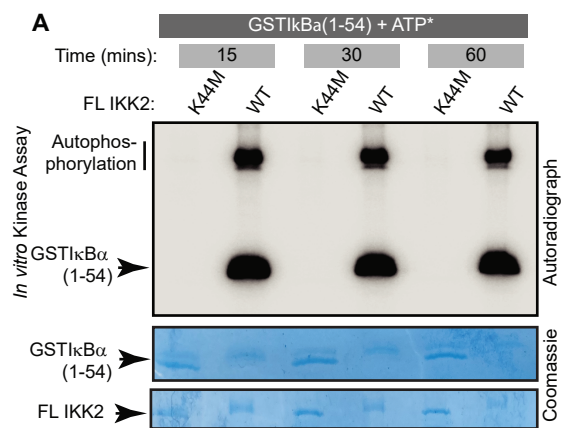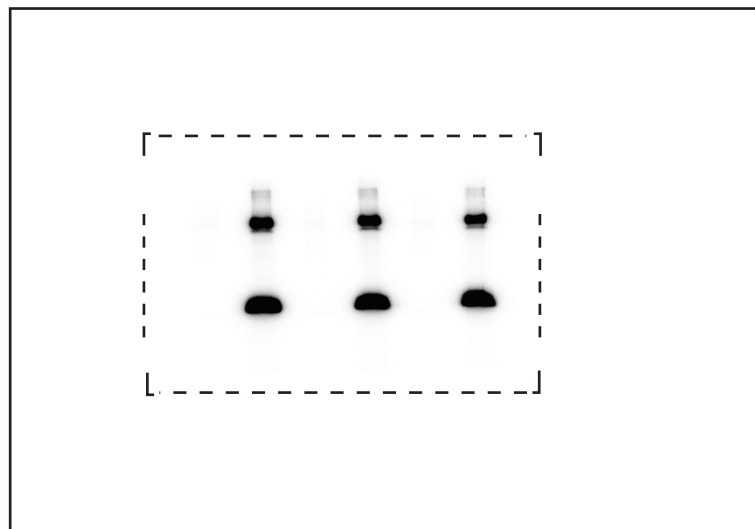

Autoradiograph

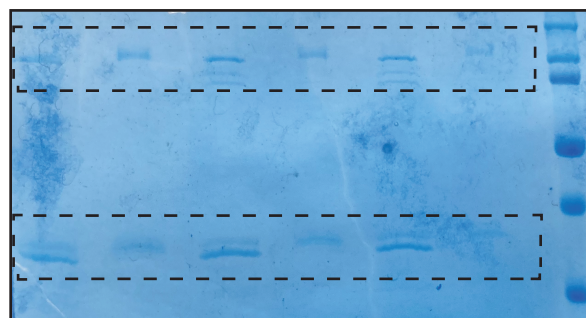

Coomassie

Supplement: Figure 1—figure supplement 1—source data 2. [file elife-98009-fig1-figsupp1-data2.zip › Figure 1-figure supplement 1-source data 2/Fig1-figure supplement 1A.pdf]

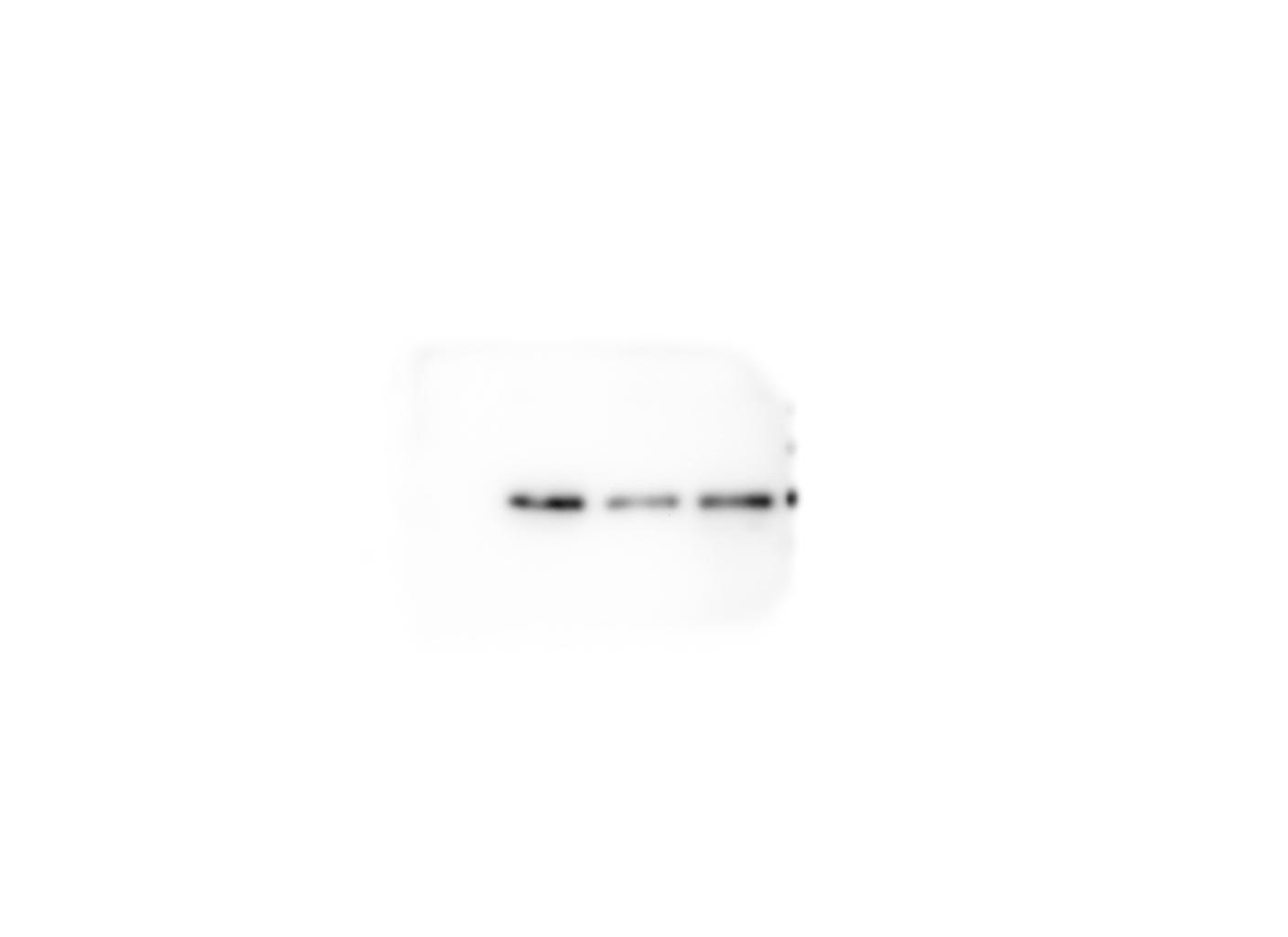

Supplement: Figure 2—source data 1. [file elife-98009-fig2-data1.zip › Figure 2-source data 1/Fig2C-6xHis.tif]

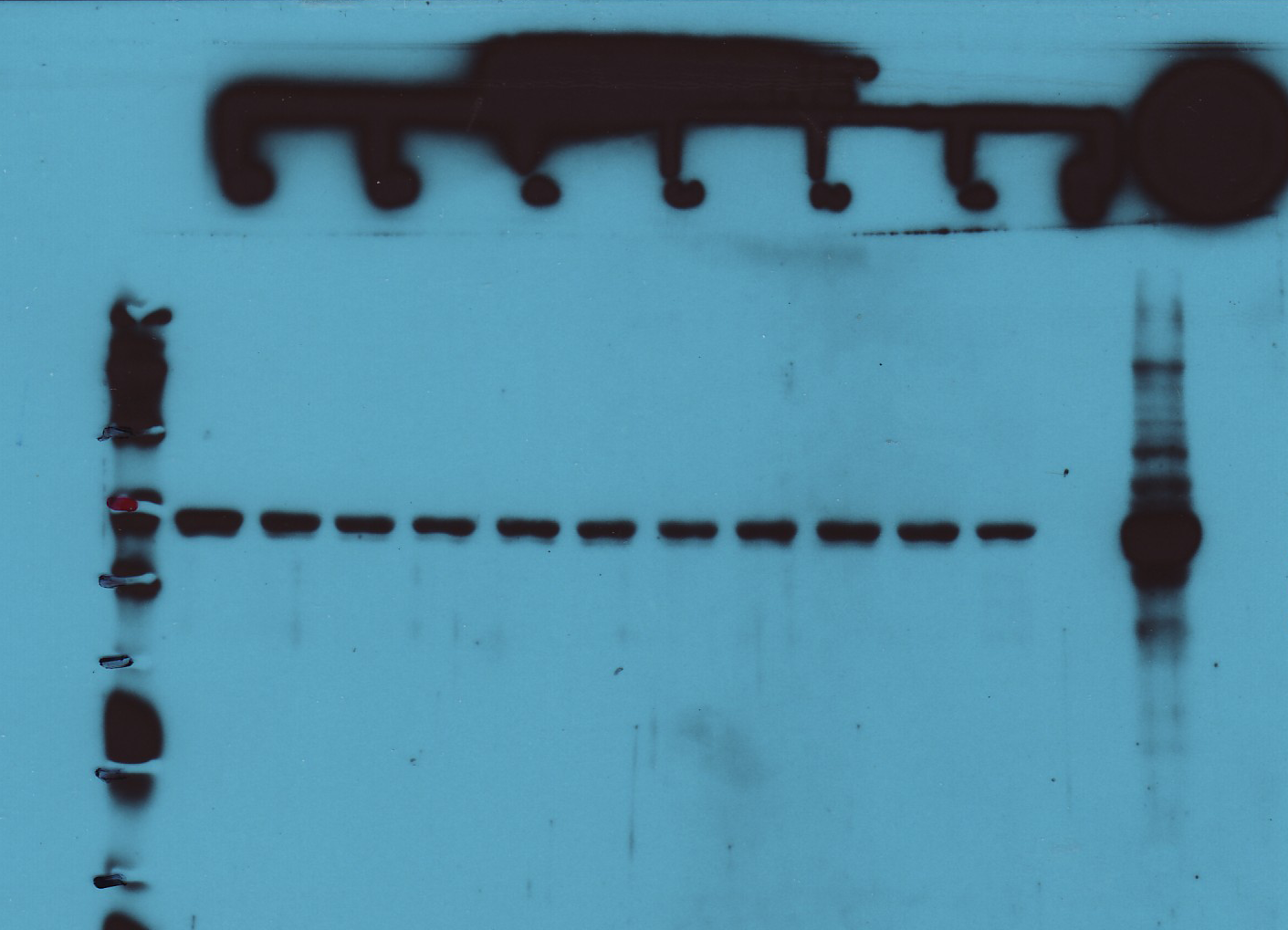

Supplement: Figure 2—source data 1. [file elife-98009-fig2-data1.zip › Figure 2-source data 1/Fig2F-pSer.tif]

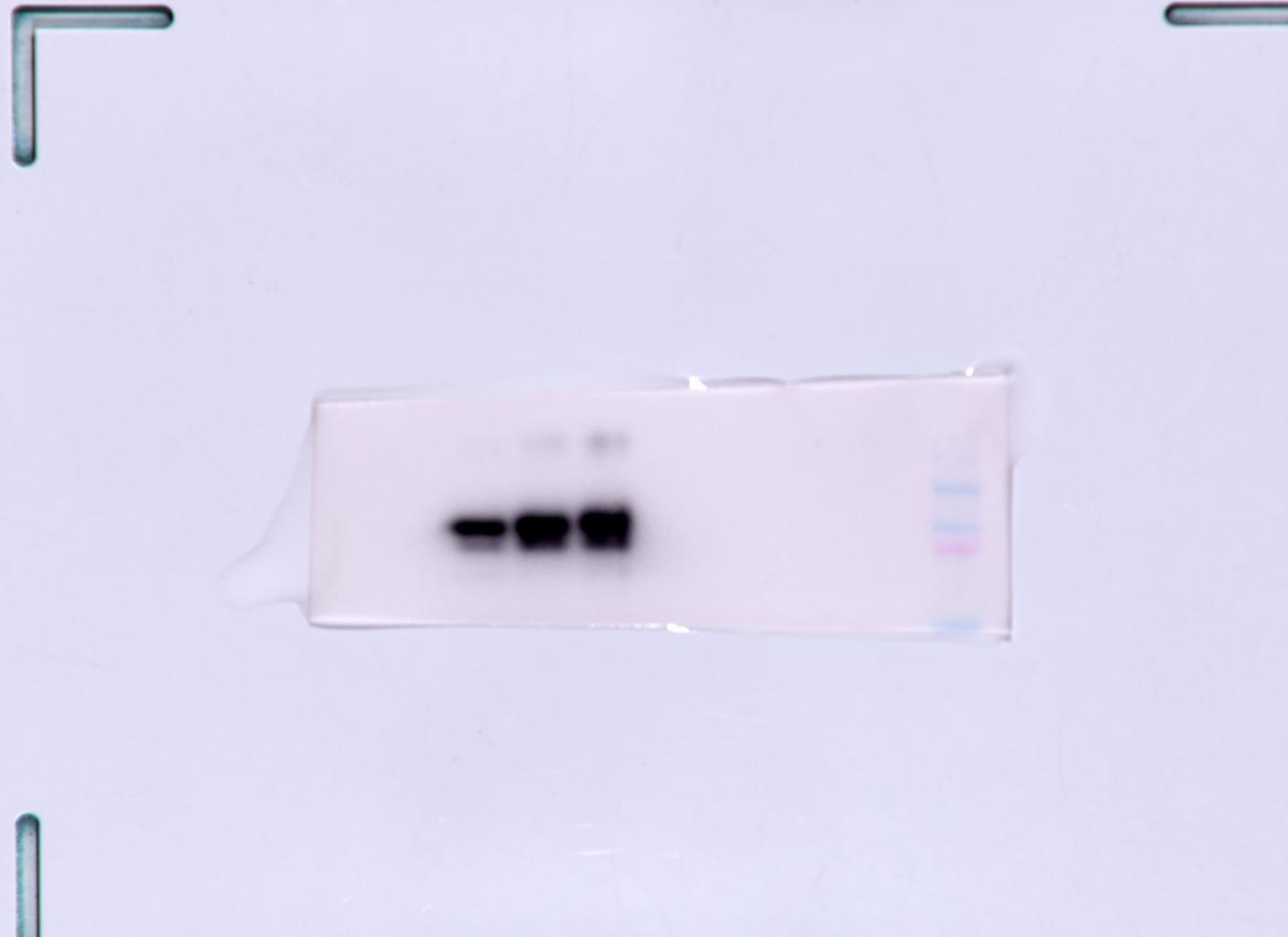

Supplement: Figure 2—source data 1. [file elife-98009-fig2-data1.zip › Figure 2-source data 1/Fig2G-pSer.tif]

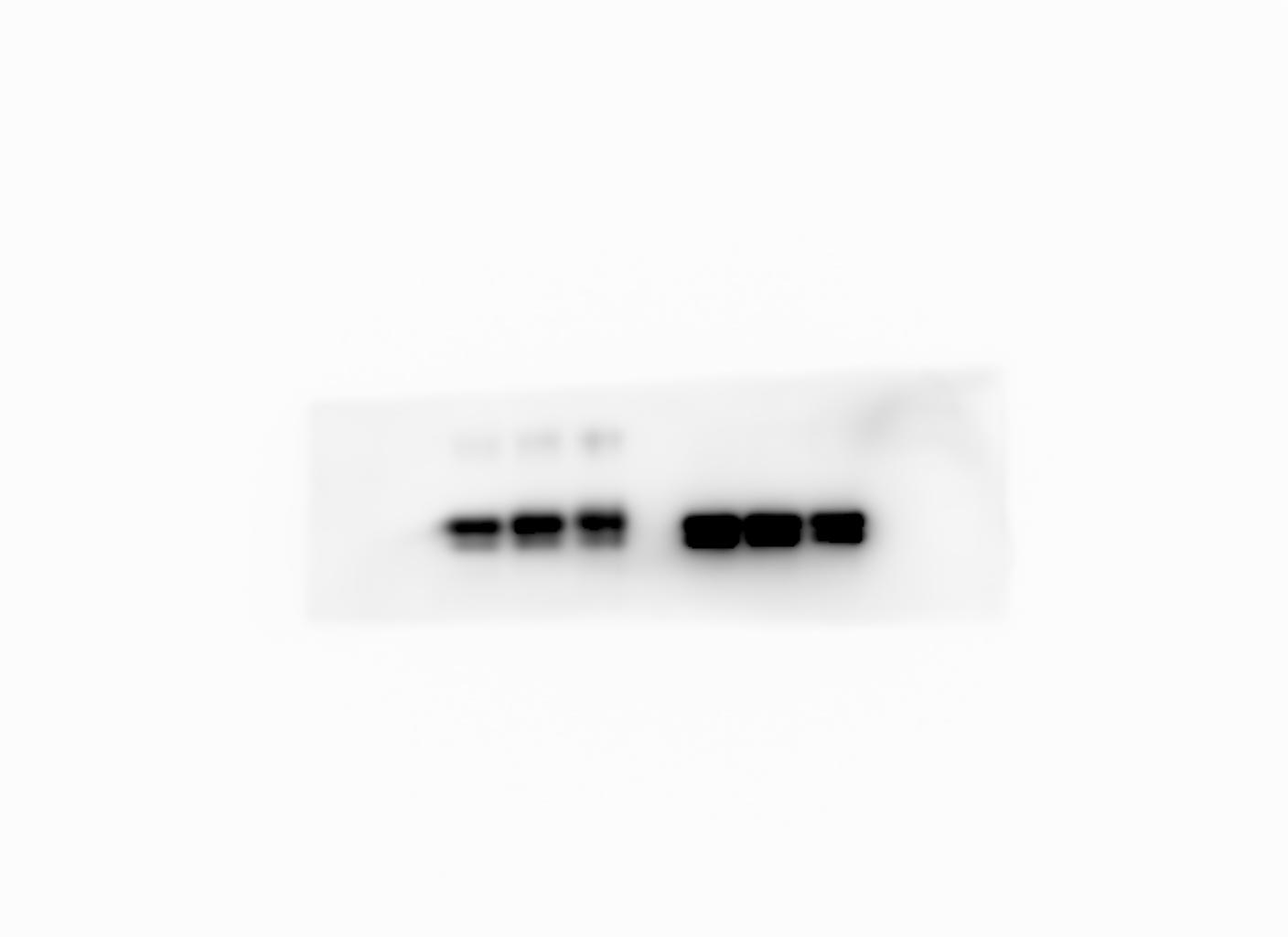

Supplement: Figure 2—source data 1. [file elife-98009-fig2-data1.zip › Figure 2-source data 1/Fig2G-IKK2.tif]

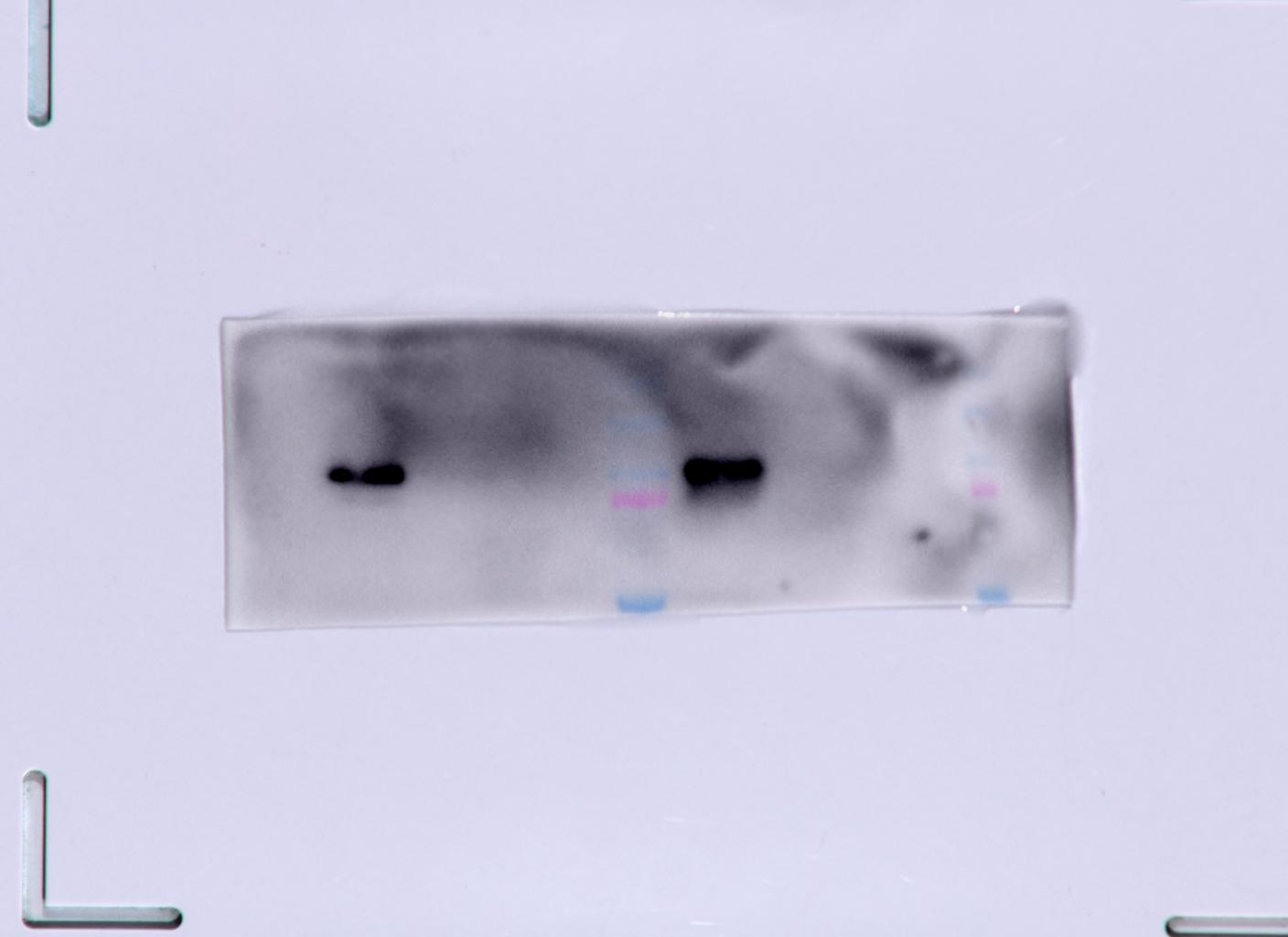

Supplement: Figure 2—source data 1. [file elife-98009-fig2-data1.zip › Figure 2-source data 1/Fig2C-pTyr.tif]

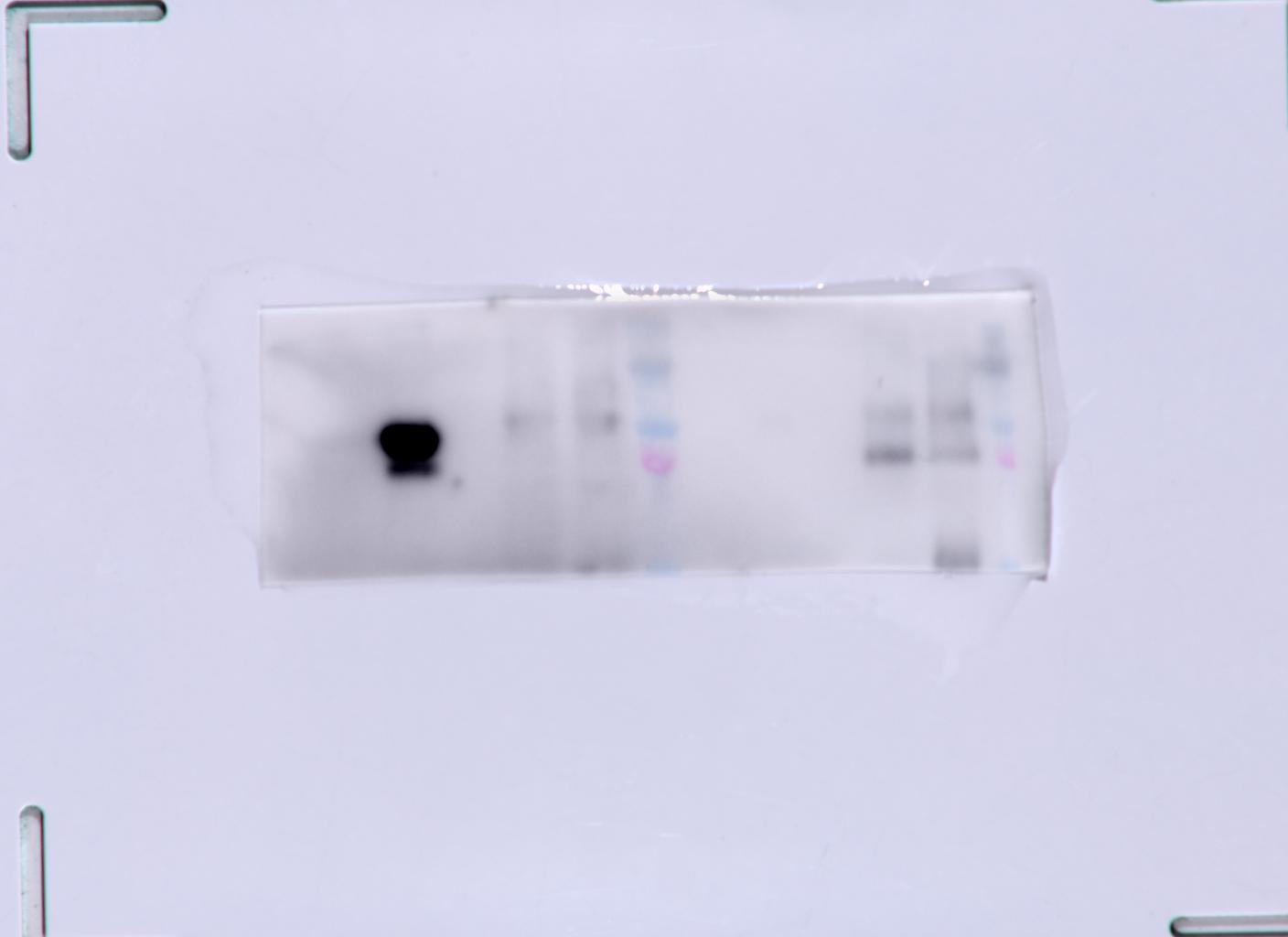

Supplement: Figure 2—source data 1. [file elife-98009-fig2-data1.zip › Figure 2-source data 1/Fig2B-pTyr.tif]

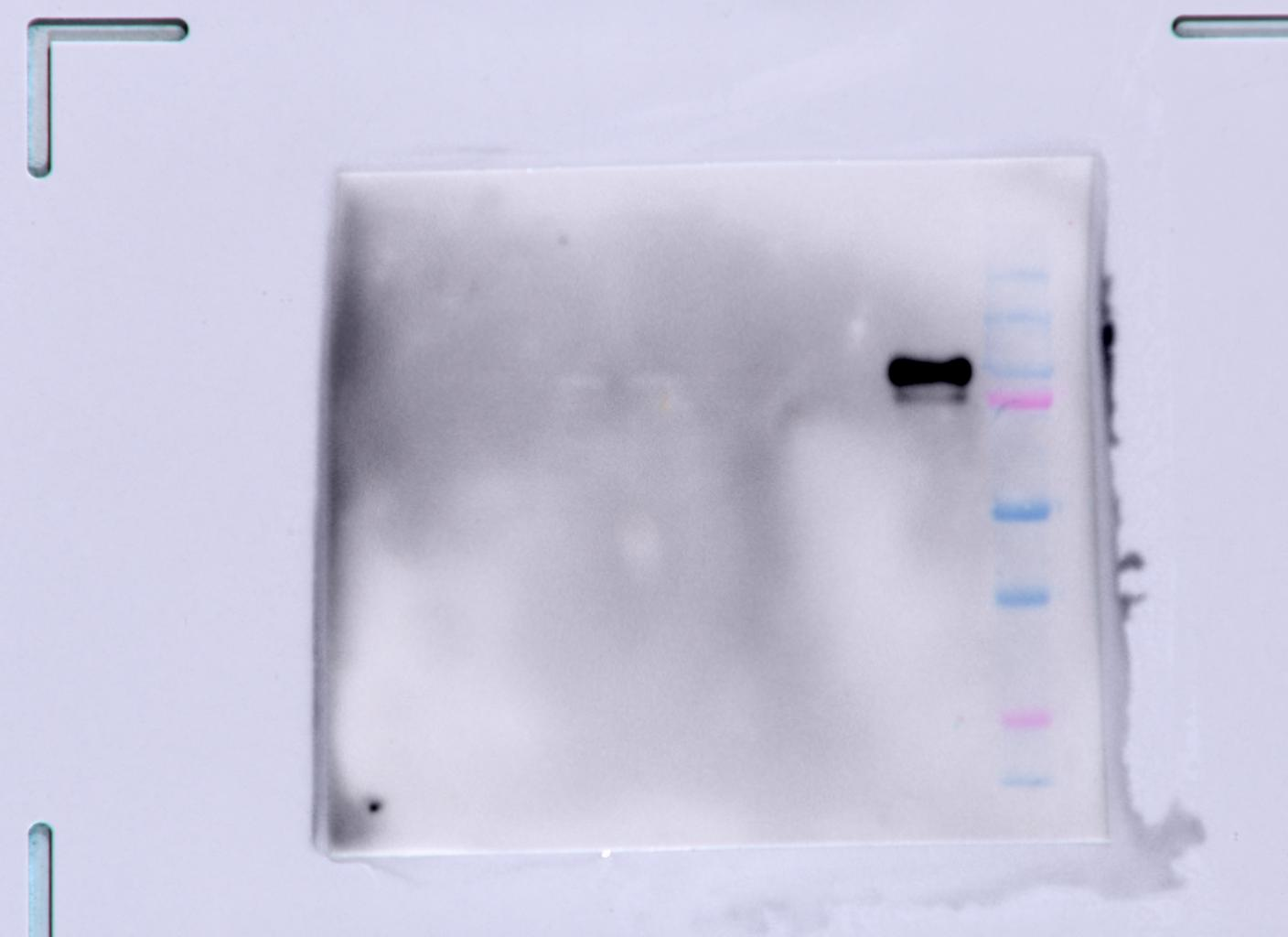

Supplement: Figure 2—source data 1. [file elife-98009-fig2-data1.zip › Figure 2-source data 1/Fig2D-pTyr.tif]

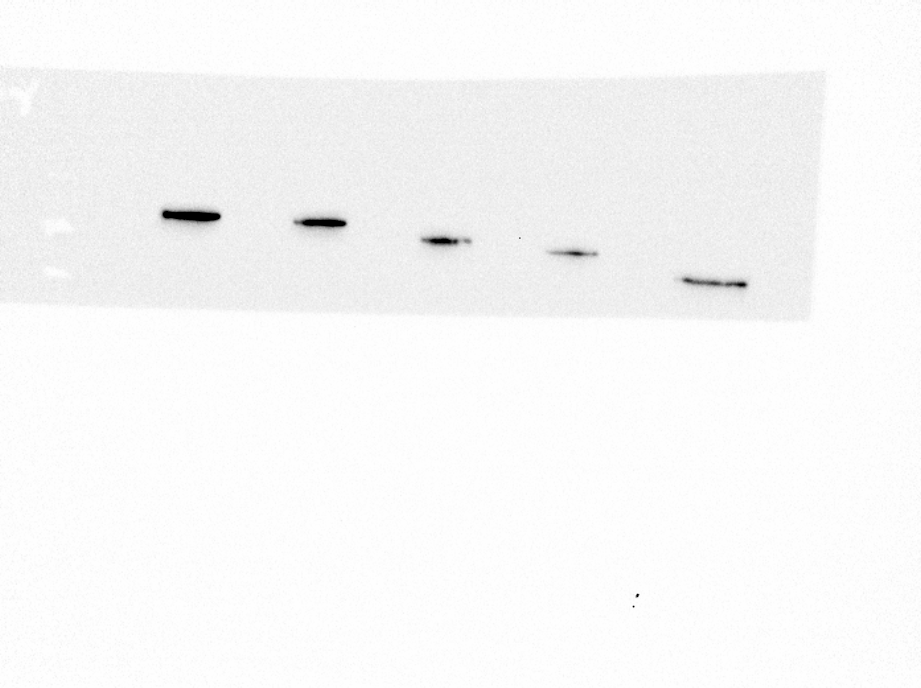

Supplement: Figure 2—source data 1. [file elife-98009-fig2-data1.zip › Figure 2-source data 1/Fig2E-pTyr.tif]

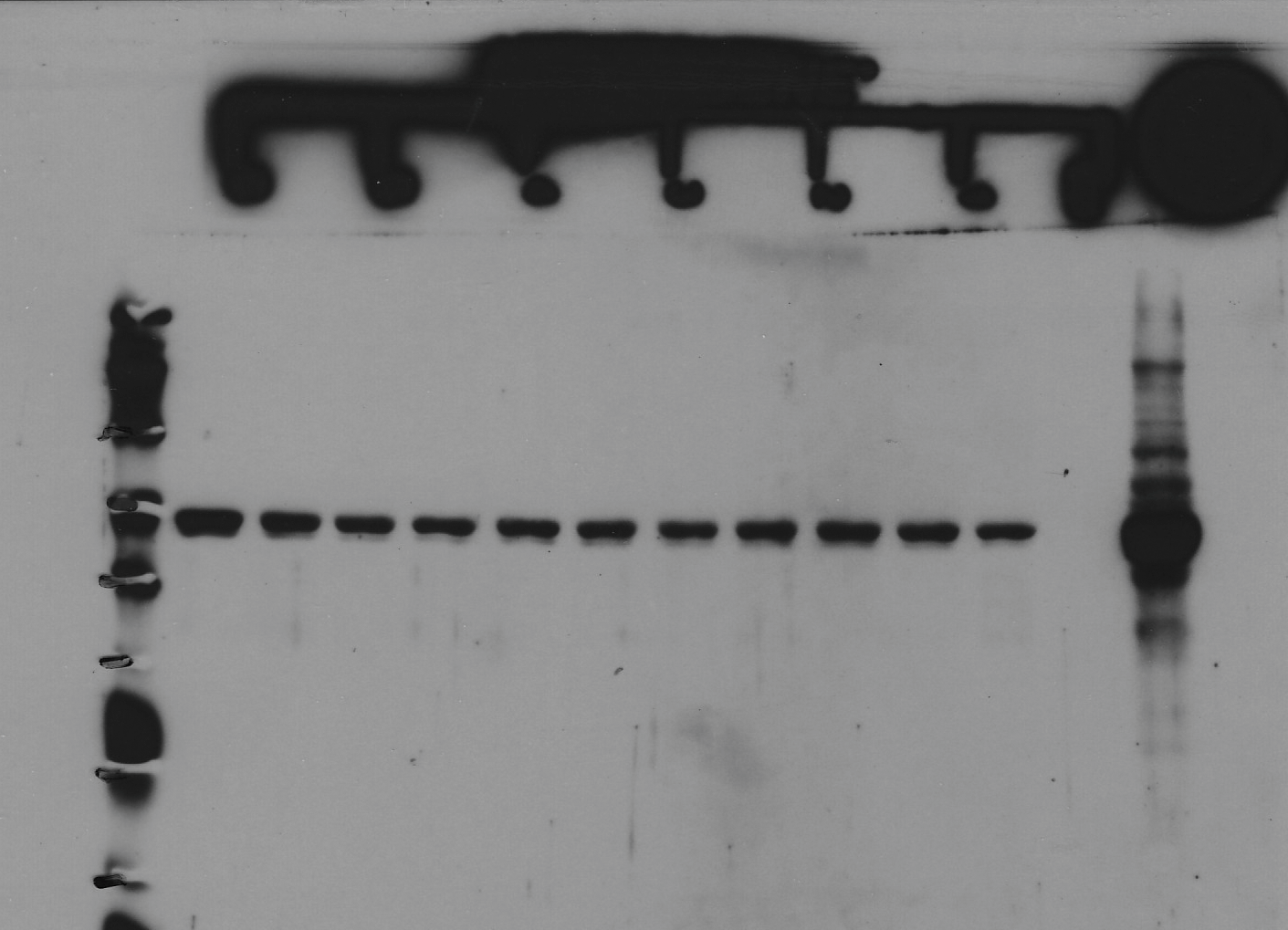

Supplement: Figure 2—source data 1. [file elife-98009-fig2-data1.zip › Figure 2-source data 1/Fig2FpSer-gray.tif]

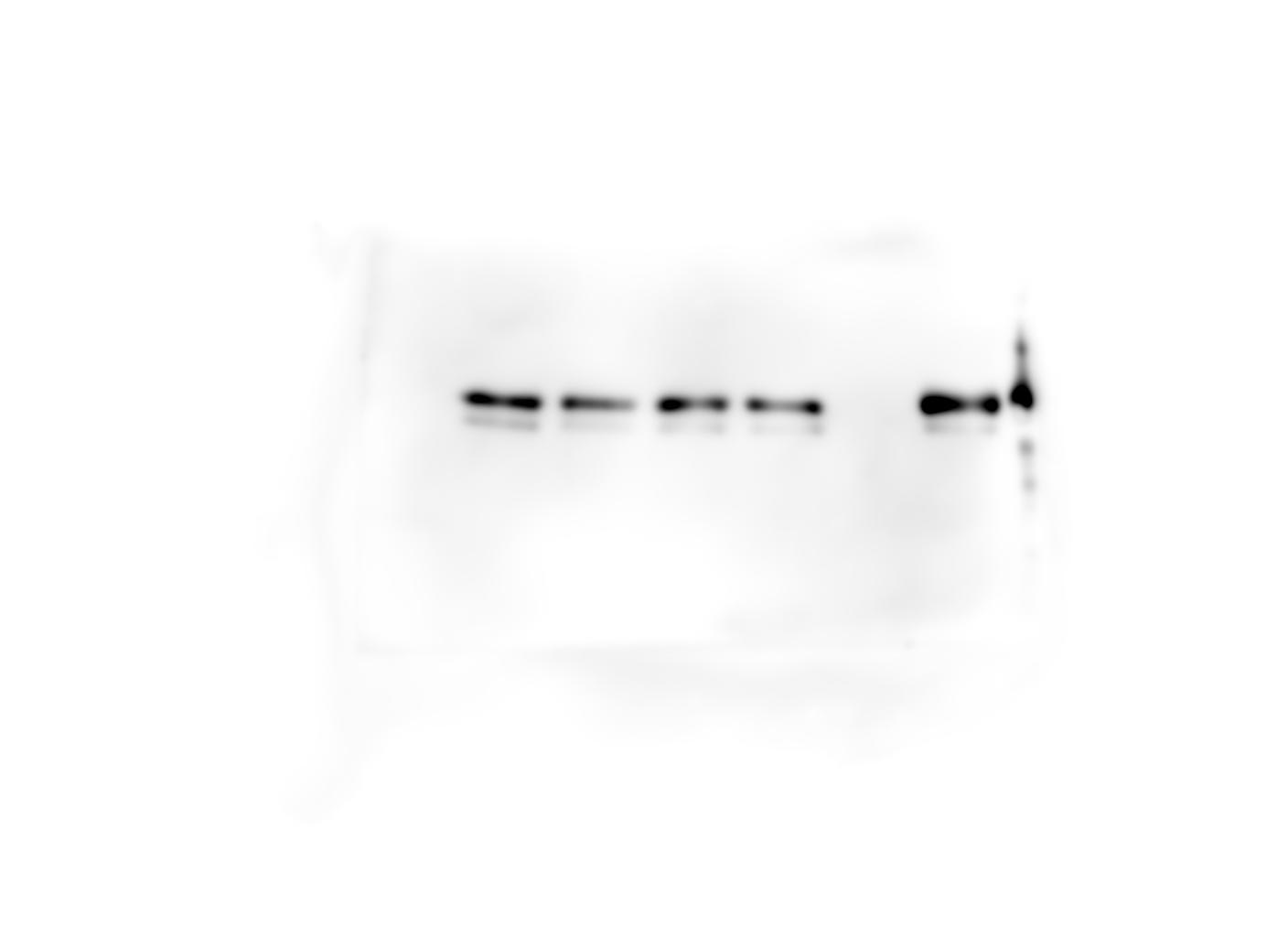

Supplement: Figure 2—source data 1. [file elife-98009-fig2-data1.zip › Figure 2-source data 1/Fig2D-6xHis.tif]

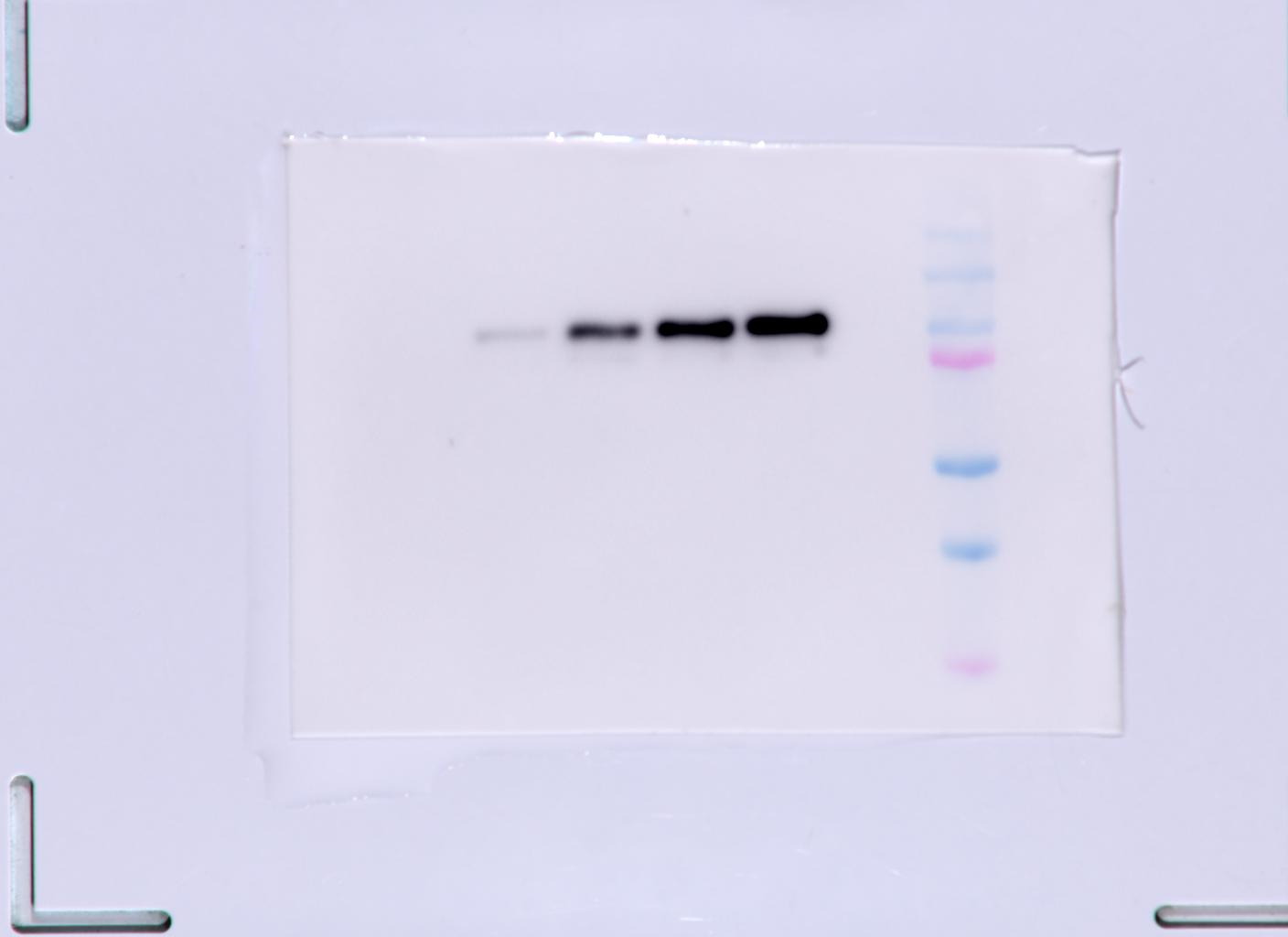

Supplement: Figure 2—source data 1. [file elife-98009-fig2-data1.zip › Figure 2-source data 1/Fig2A-pTyr.tif]

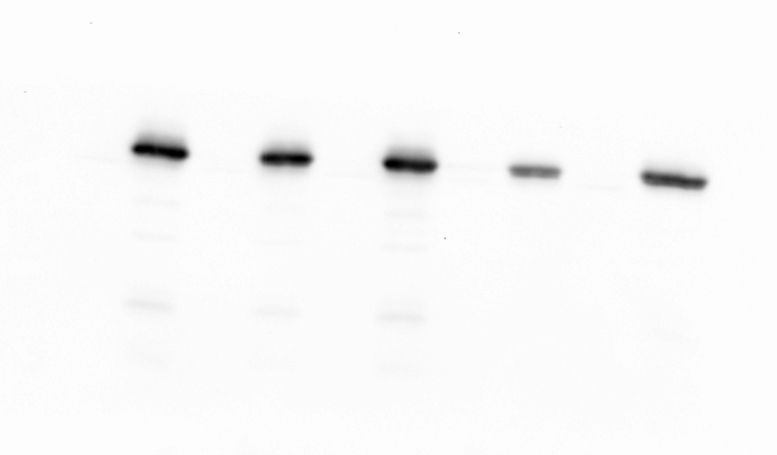

Supplement: Figure 2—source data 1. [file elife-98009-fig2-data1.zip › Figure 2-source data 1/Fig2E-pIkBa.tif]

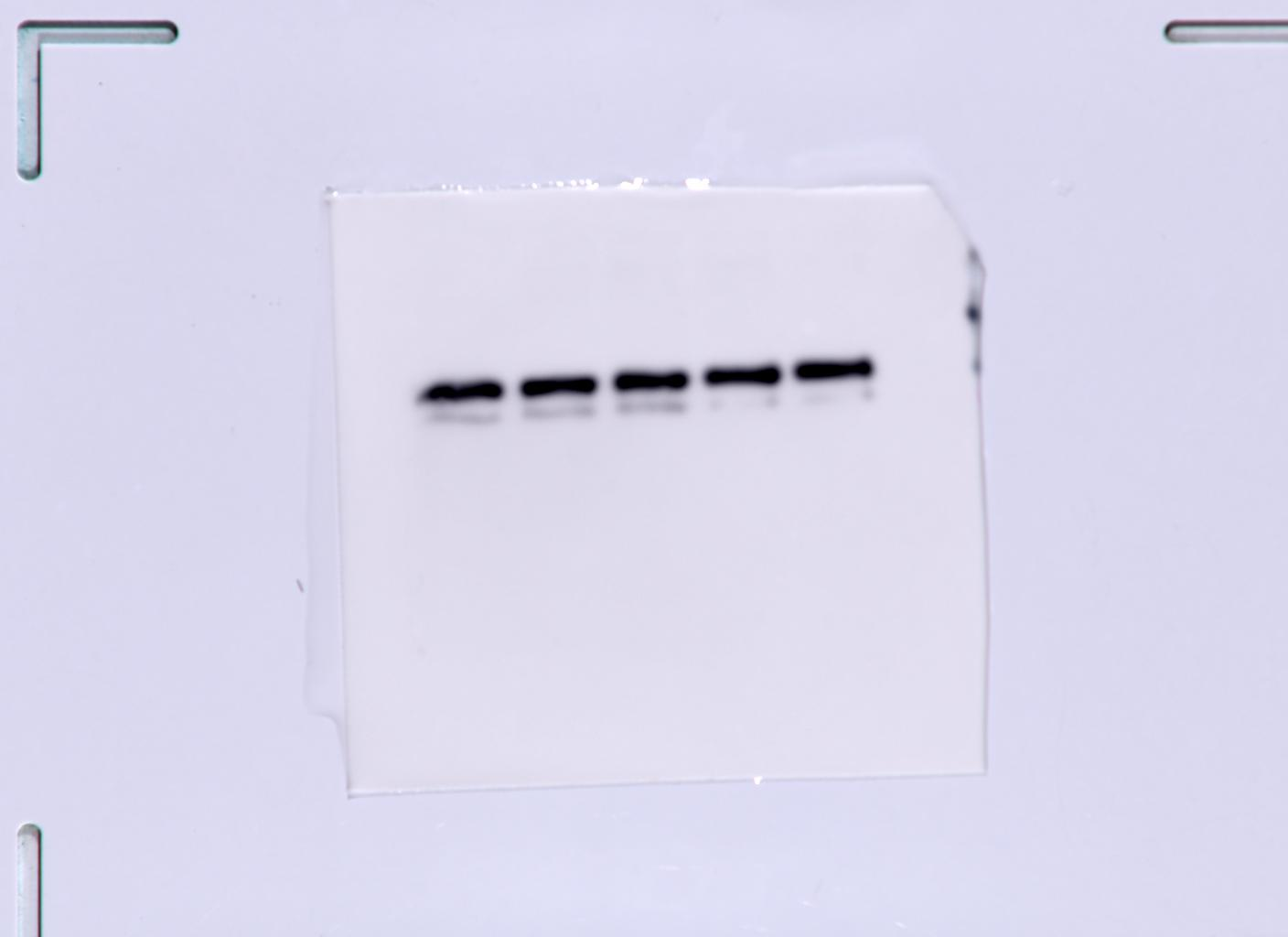

Supplement: Figure 2—source data 1. [file elife-98009-fig2-data1.zip › Figure 2-source data 1/Fig2A-6xHis.tif]

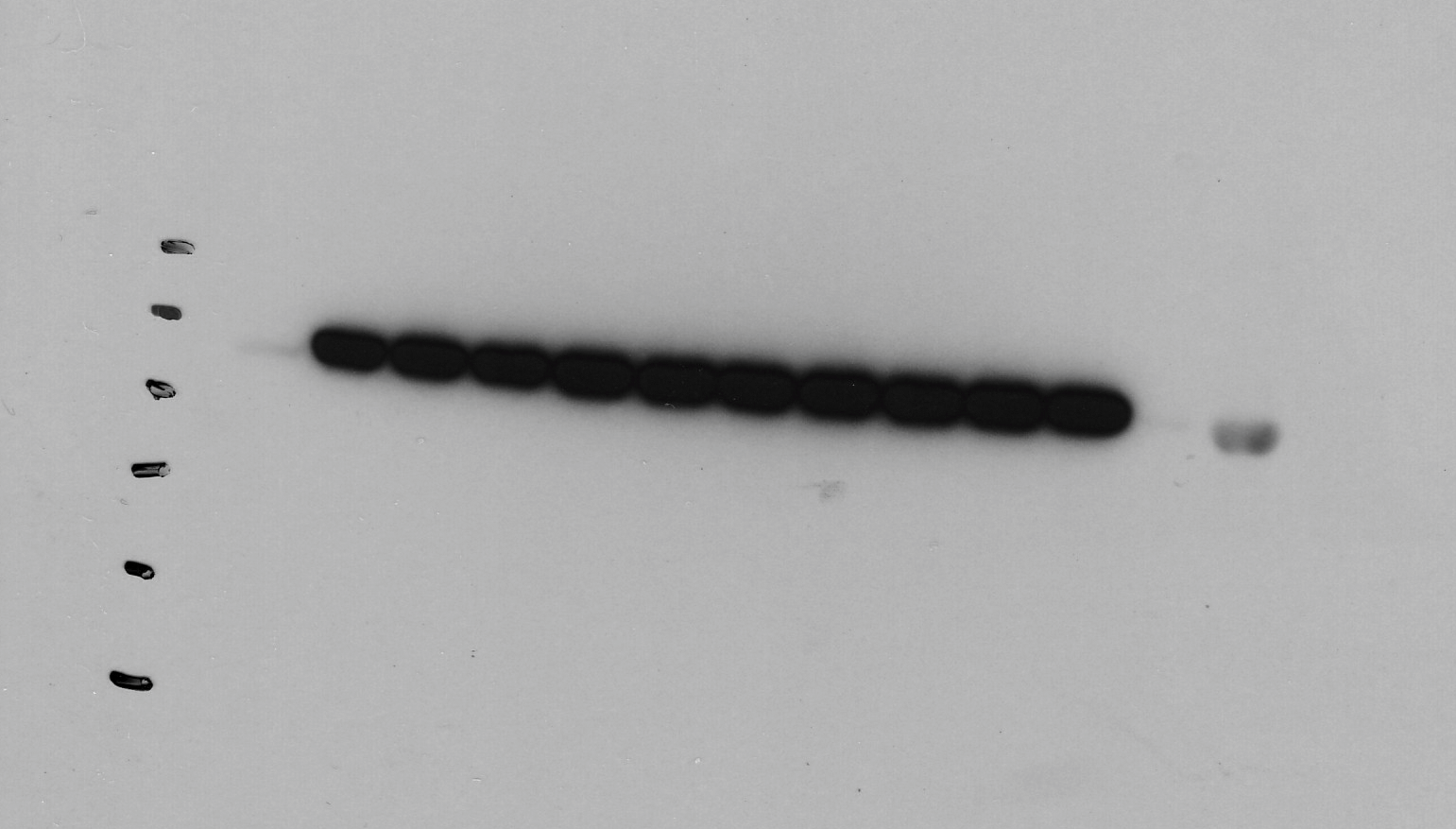

Supplement: Figure 2—source data 1. [file elife-98009-fig2-data1.zip › Figure 2-source data 1/Fig2F-pTyr-gray.tif]

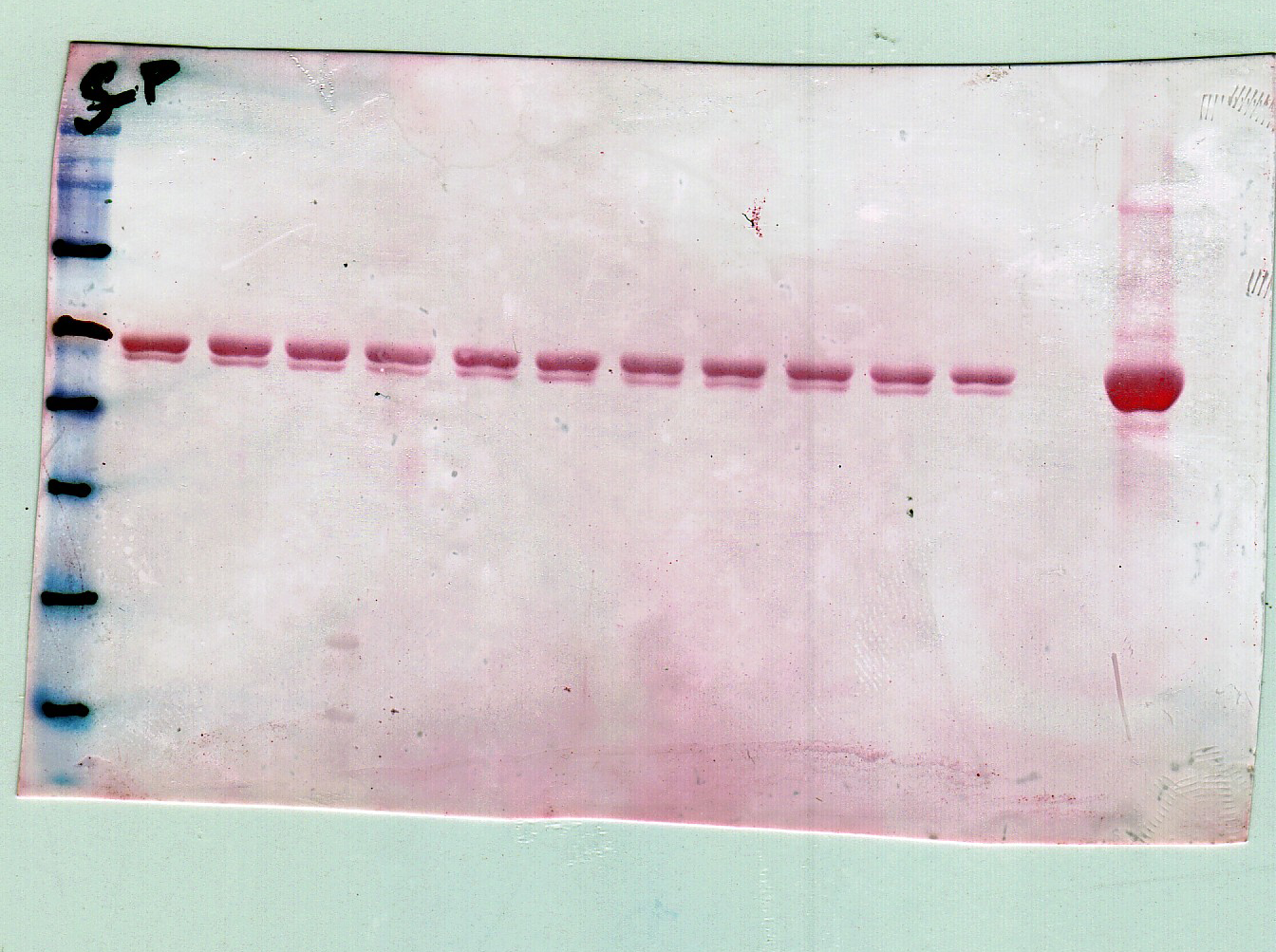

Supplement: Figure 2—source data 1. [file elife-98009-fig2-data1.zip › Figure 2-source data 1/Fig2F-Ponceau.tif]

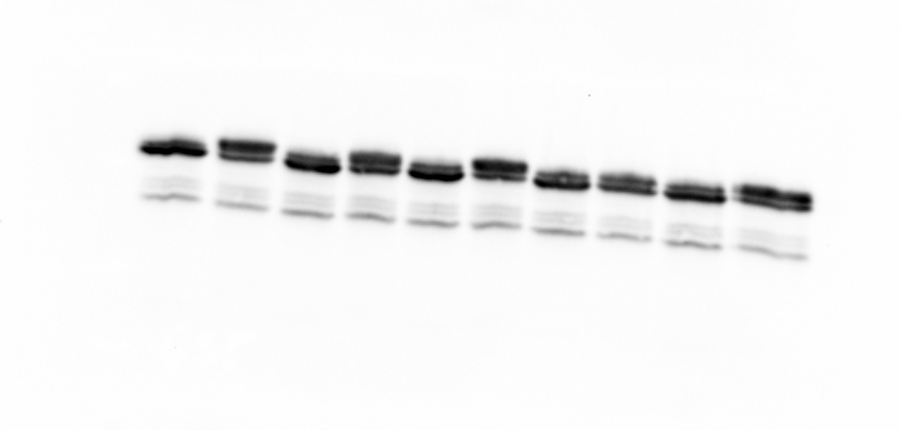

Supplement: Figure 2—source data 1. [file elife-98009-fig2-data1.zip › Figure 2-source data 1/Fig2E-GST.tif]

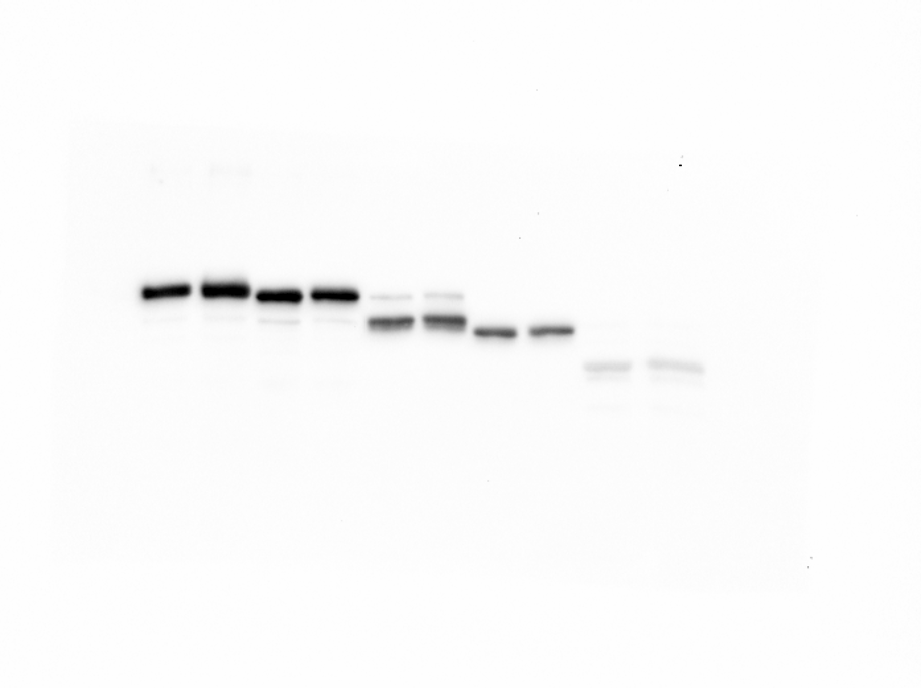

Supplement: Figure 2—source data 1. [file elife-98009-fig2-data1.zip › Figure 2-source data 1/Fig2E-IKK.tif]

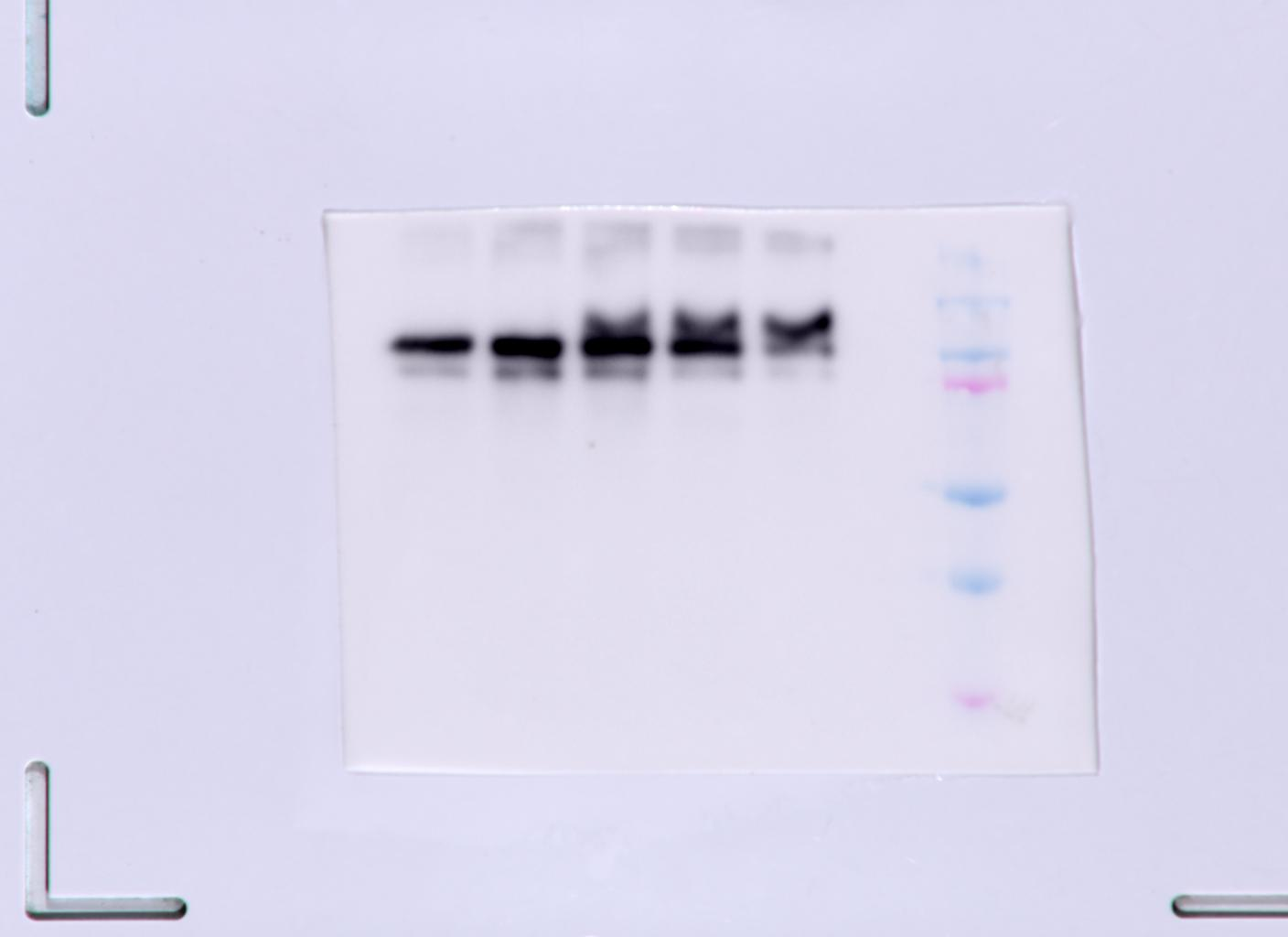

Supplement: Figure 2—source data 1. [file elife-98009-fig2-data1.zip › Figure 2-source data 1/Fig2A-pSerIKK2.tif]

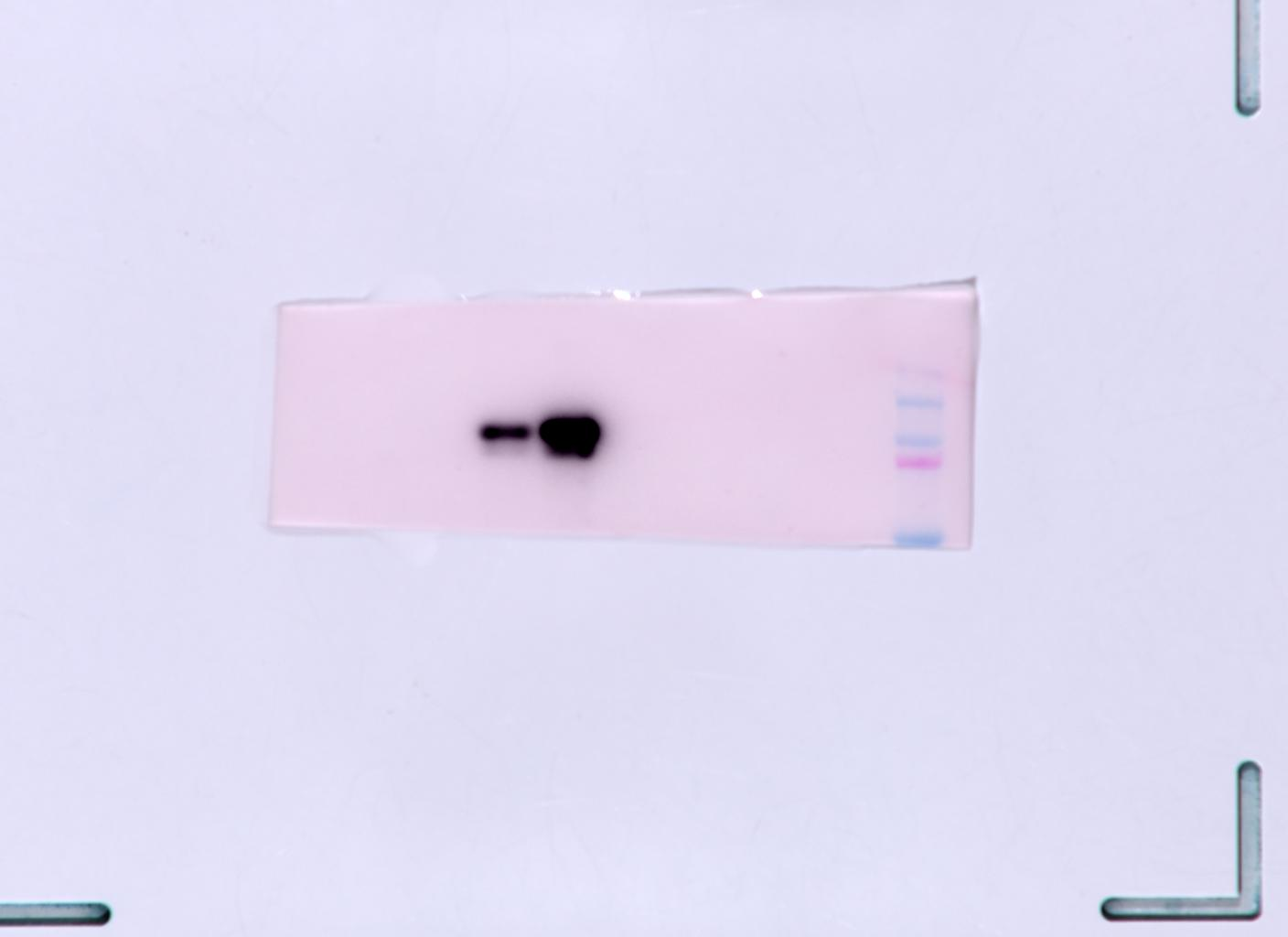

Supplement: Figure 2—source data 1. [file elife-98009-fig2-data1.zip › Figure 2-source data 1/Fig2G-pTyr.tif]

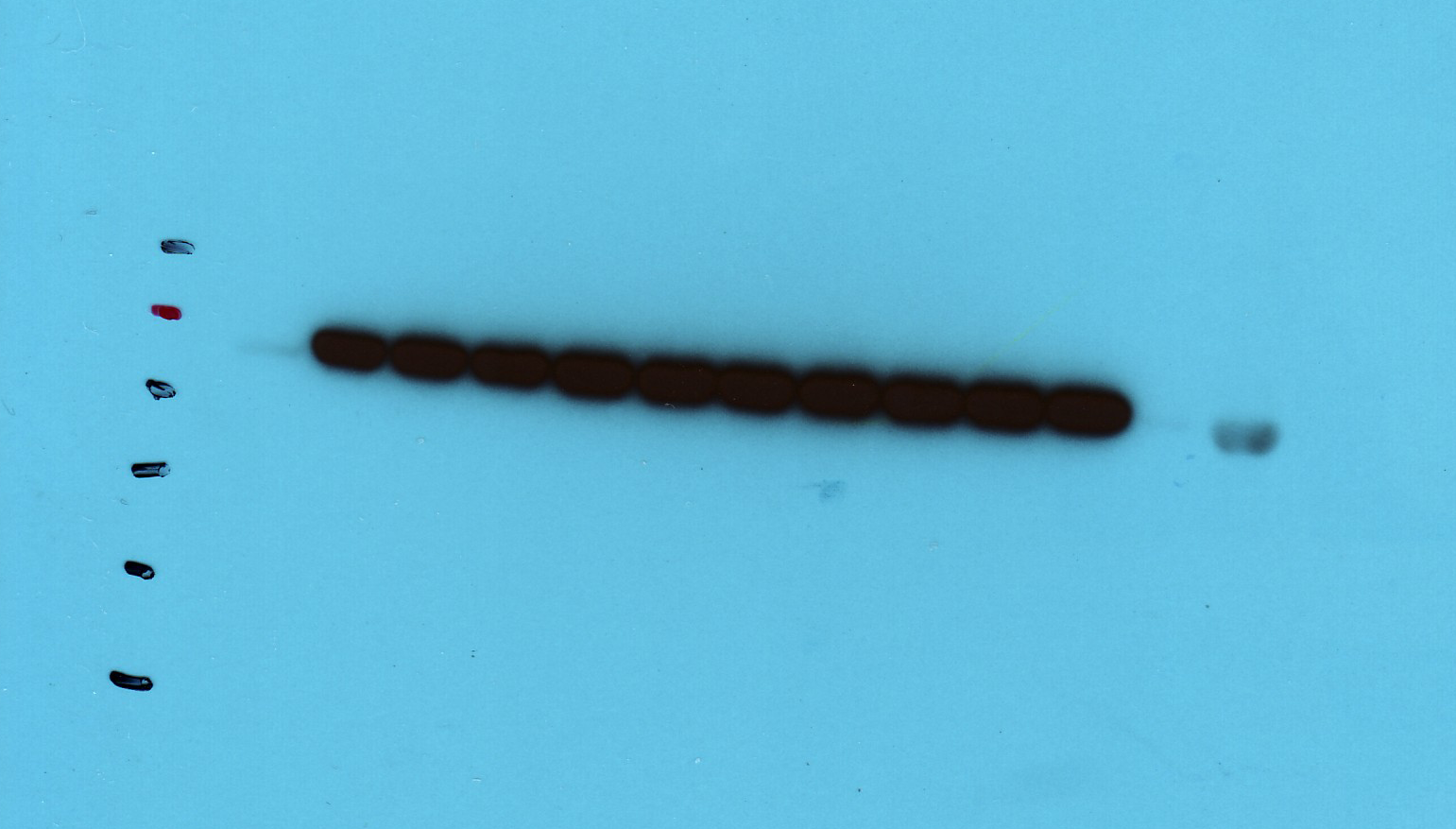

Supplement: Figure 2—source data 1. [file elife-98009-fig2-data1.zip › Figure 2-source data 1/Fig2F-pTyr.tif]

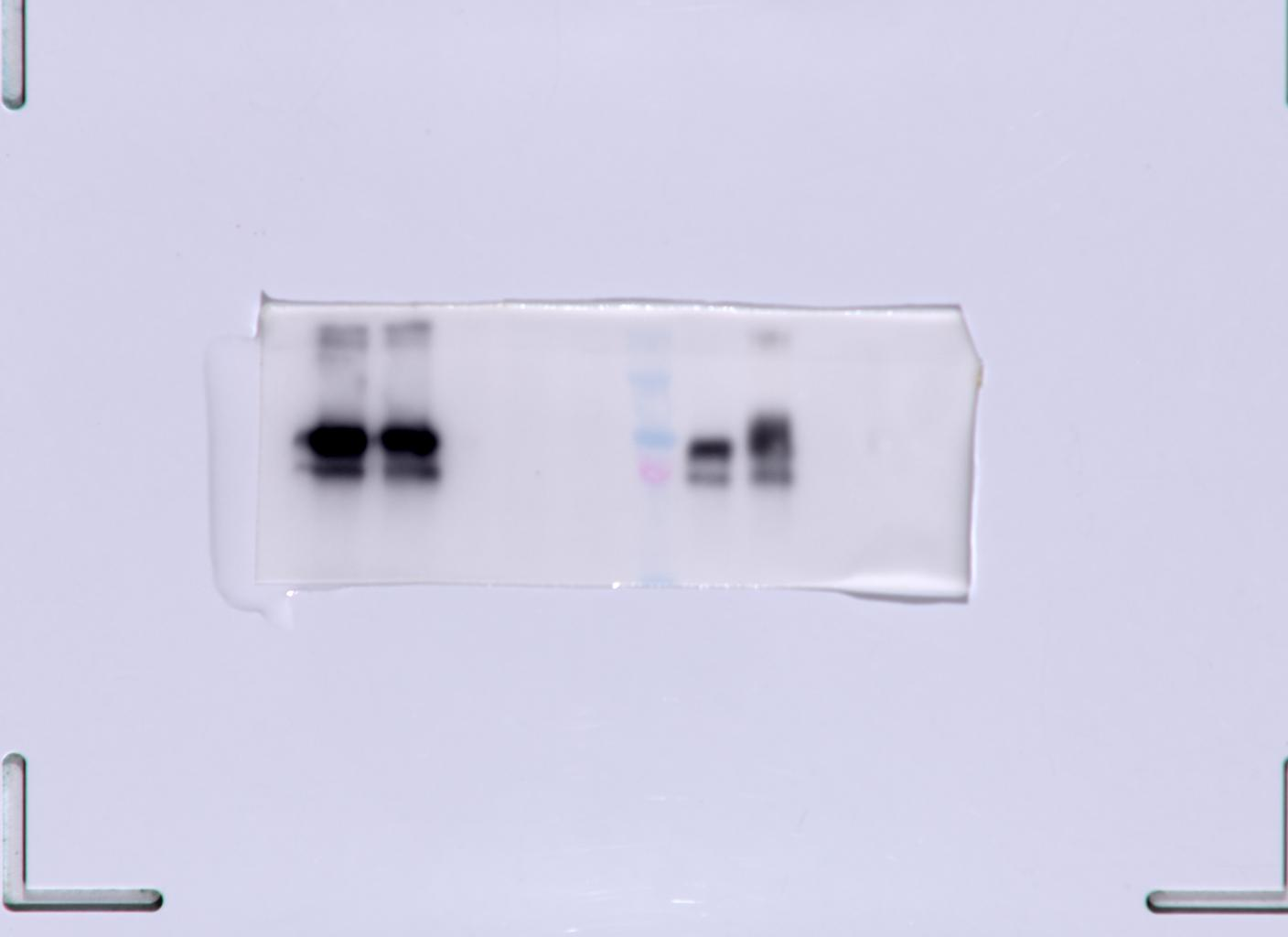

Supplement: Figure 2—source data 1. [file elife-98009-fig2-data1.zip › Figure 2-source data 1/Fig2B-IKK2.tif]

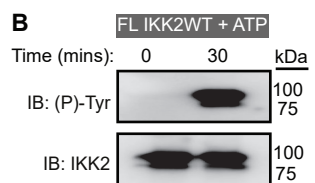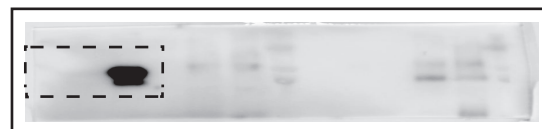

IB: (P)-Tyr

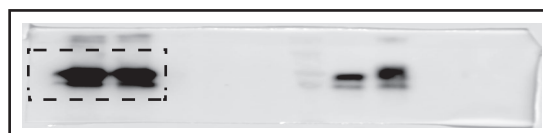

IB: IKK2

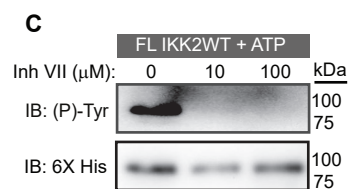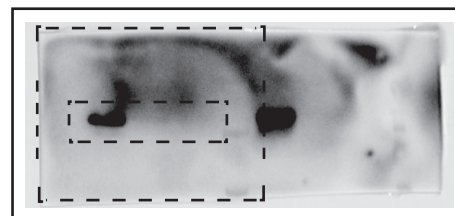

IB: (P)-Tyr

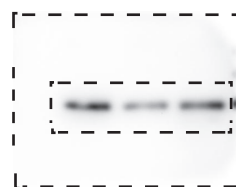

IB: 6X His

Supplement: Figure 2—source data 2. [file elife-98009-fig2-data2.zip › Figure 2-source data 2/Fig2B and Fig2C.pdf]

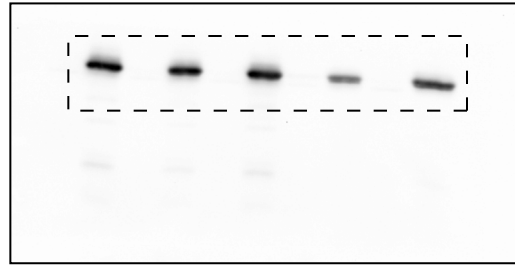

IB: (P)-IκBα

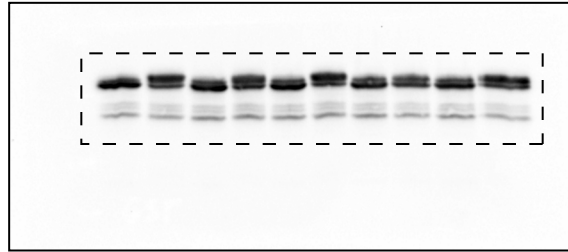

IB: GST

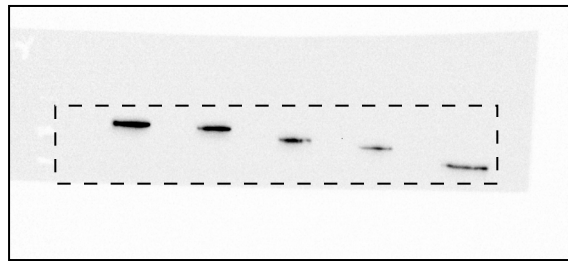

IB: (P)-Tyr

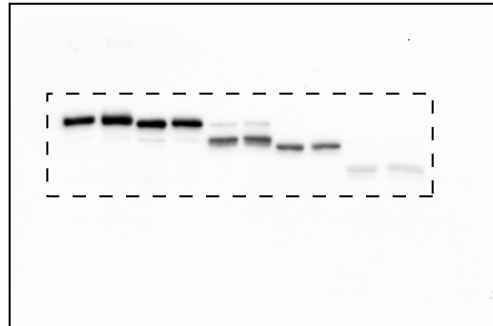

IB: IKK1/2

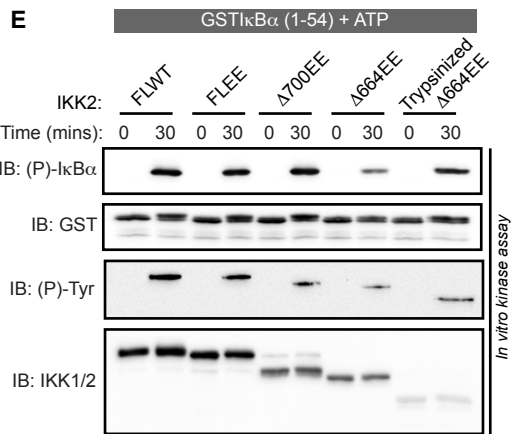

Supplement: Figure 2—source data 2. [file elife-98009-fig2-data2.zip › Figure 2-source data 2/Fig2E.pdf]

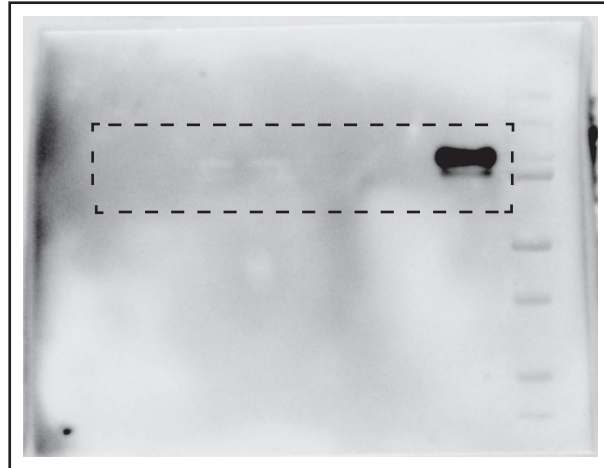

IB: (P)-Tyr

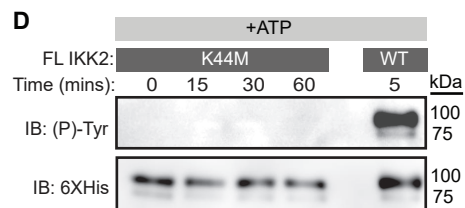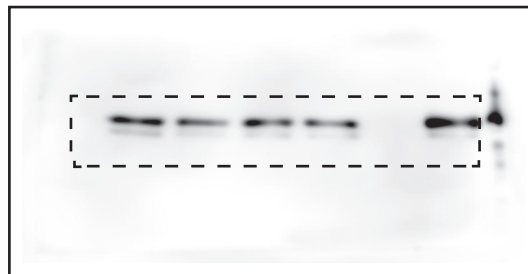

IB: 6XHis

Supplement: Figure 2—source data 2. [file elife-98009-fig2-data2.zip › Figure 2-source data 2/Fig2D.pdf]

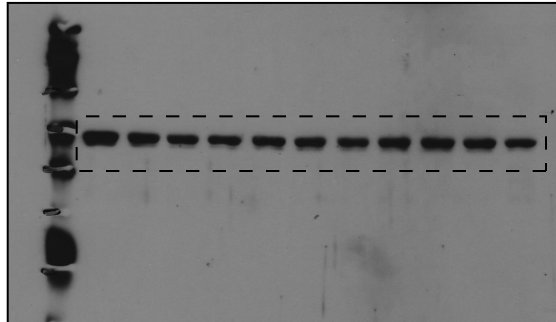

IB: (P)-Ser

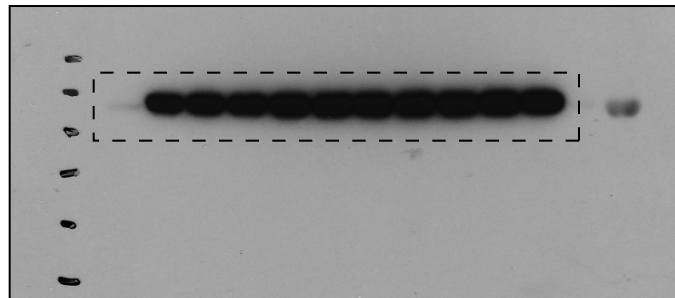

IB: (P)-Tyr

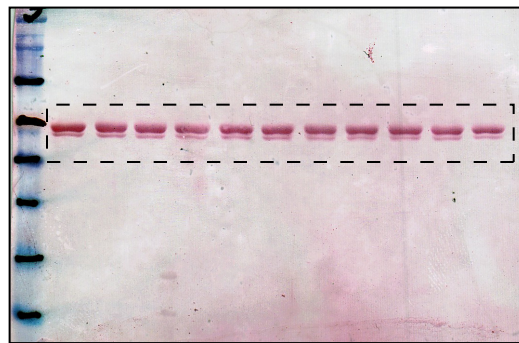

Ponceau

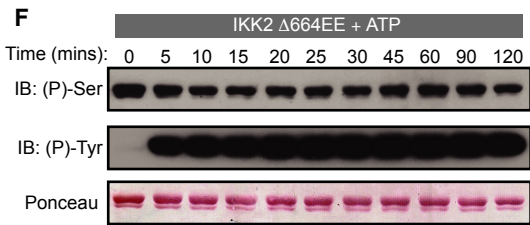

Supplement: Figure 2—source data 2. [file elife-98009-fig2-data2.zip › Figure 2-source data 2/Fig2F.pdf]

**G**

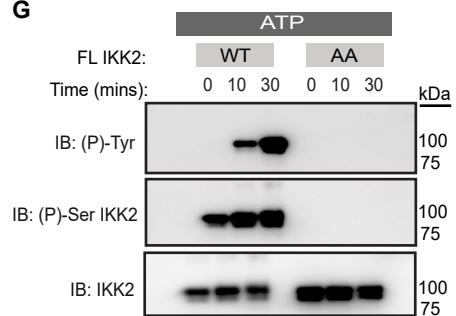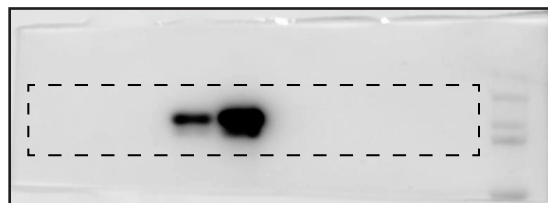

IB: (P)-Tyr

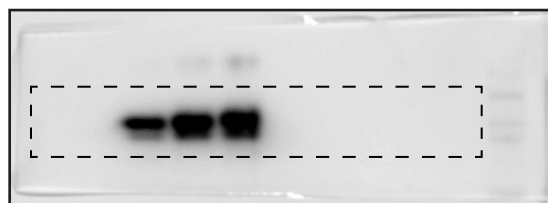

IB: (P)-Ser IKK2

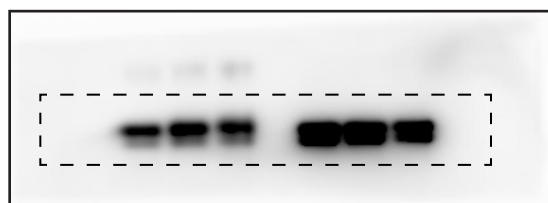

IB: IKK2

Supplement: Figure 2—source data 2. [file elife-98009-fig2-data2.zip › Figure 2-source data 2/Fig2G.pdf]

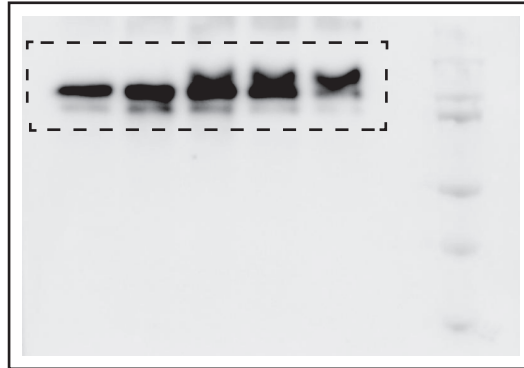

IB: (P)-Ser IKK2

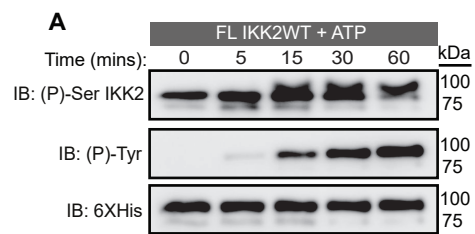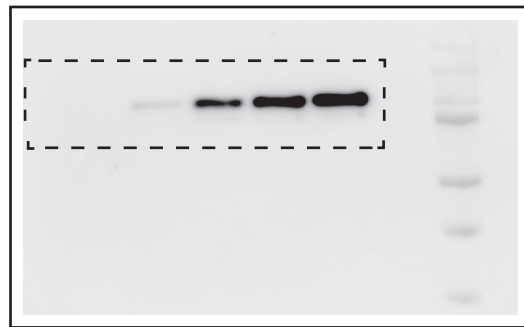

IB: (P)-Tyr

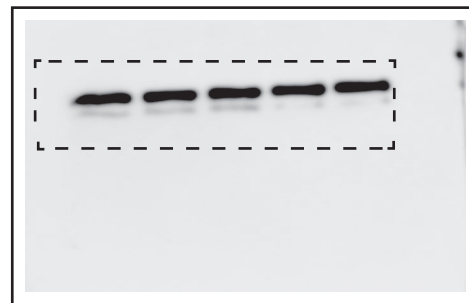

IB: 6XHis

Supplement: Figure 2—source data 2. [file elife-98009-fig2-data2.zip › Figure 2-source data 2/Fig2A.pdf]

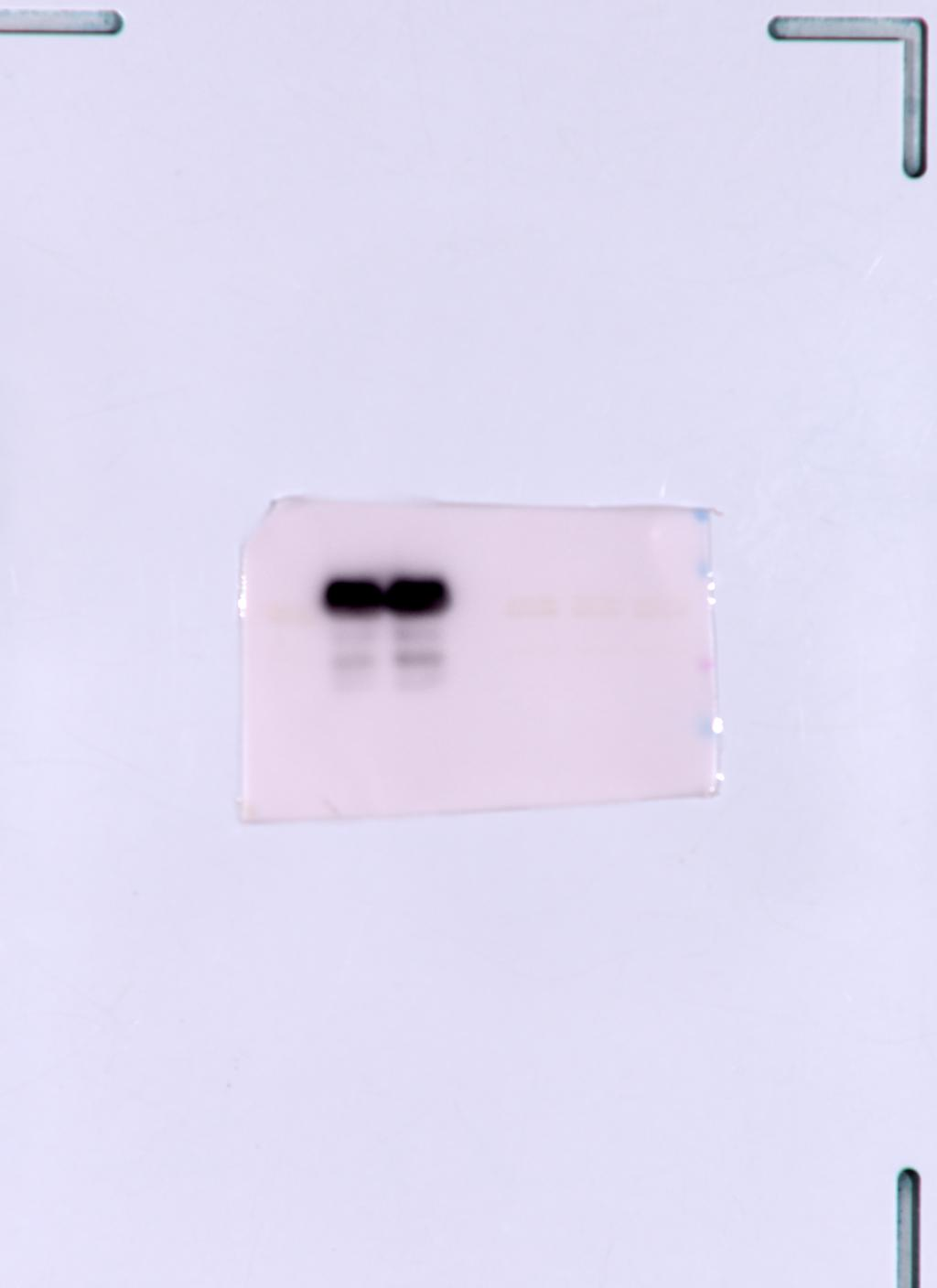

Supplement: Figure 2—figure supplement 1—source data 1. [file elife-98009-fig2-figsupp1-data1.zip › Figure 2-figure supplement 1-source data 1/Fig2-fig supp 1D-pIkBa.tif]

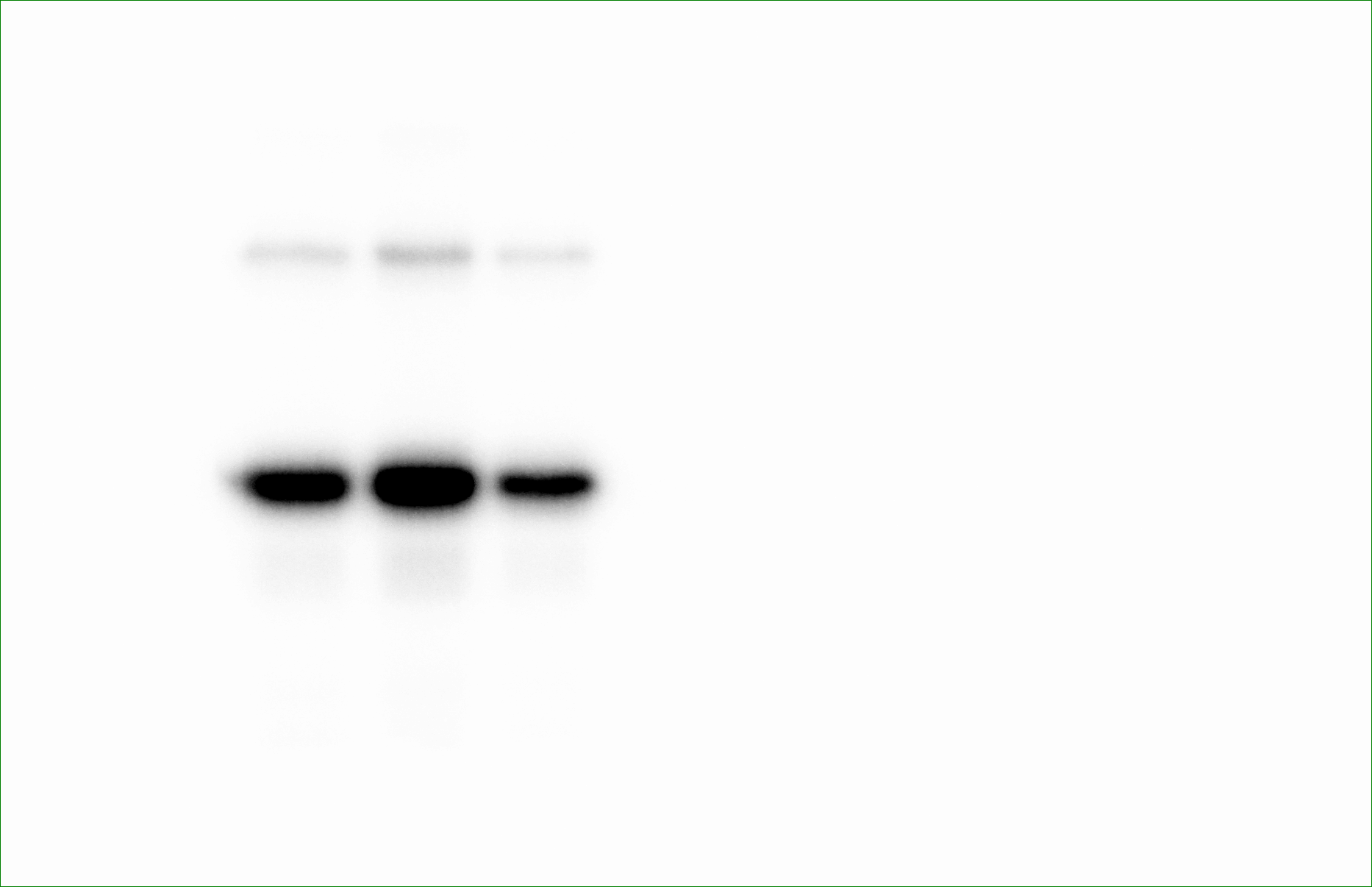

Supplement: Figure 2—figure supplement 1—source data 1. [file elife-98009-fig2-figsupp1-data1.zip › Figure 2-figure supplement 1-source data 1/Fig2-fig supp 1B-autorad.tif]

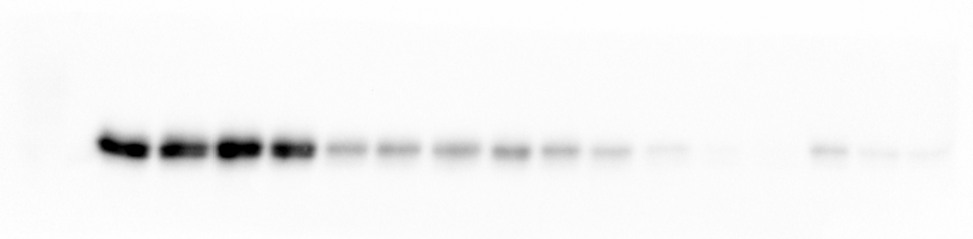

Supplement: Figure 2—figure supplement 1—source data 1. [file elife-98009-fig2-figsupp1-data1.zip › Figure 2-figure supplement 1-source data 1/Fig2-fig supp 1C-pIkBa.tif]

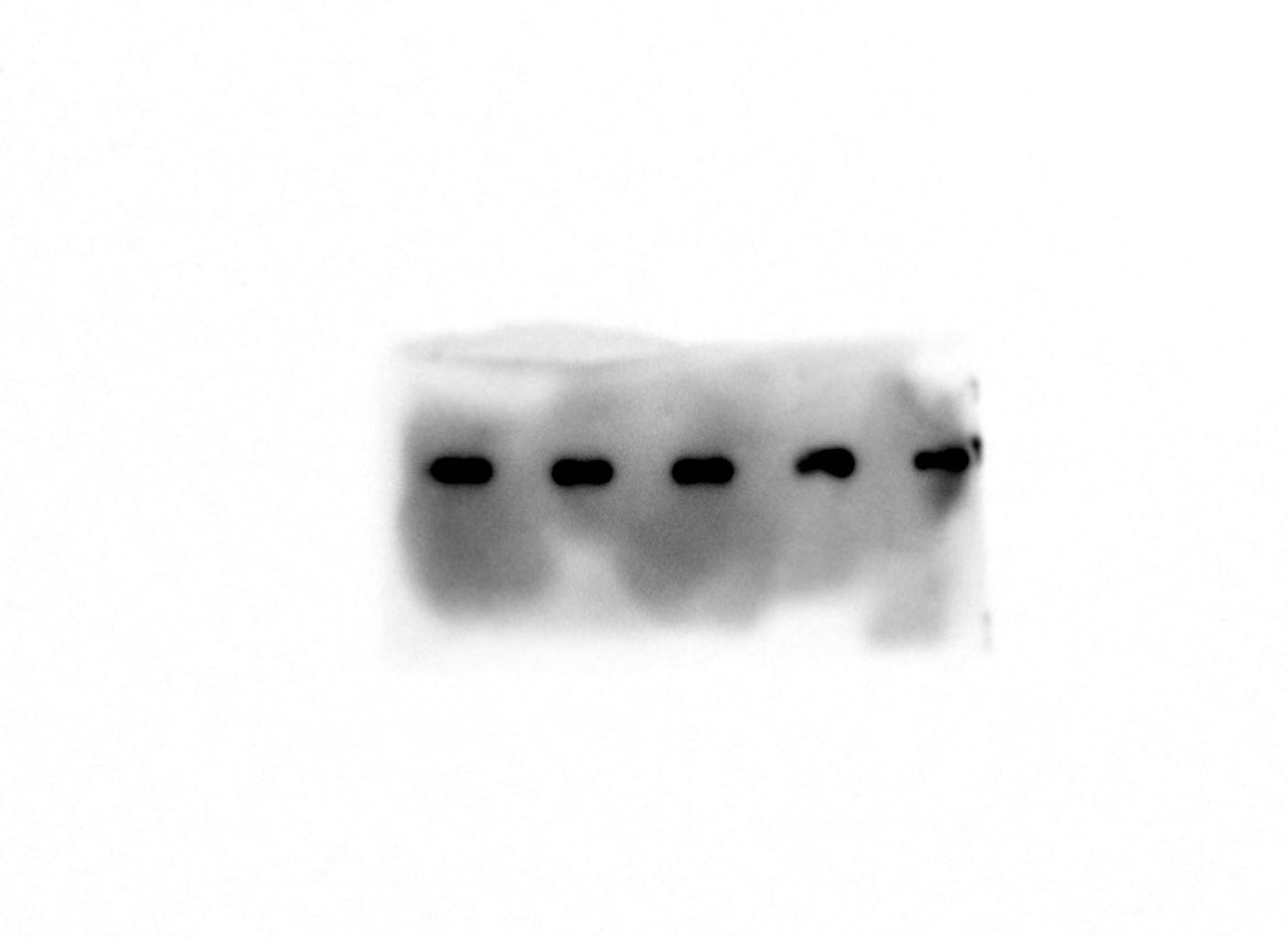

Supplement: Figure 2—figure supplement 1—source data 1. [file elife-98009-fig2-figsupp1-data1.zip › Figure 2-figure supplement 1-source data 1/Fig2-fig supp 1A-6xHis.tif]

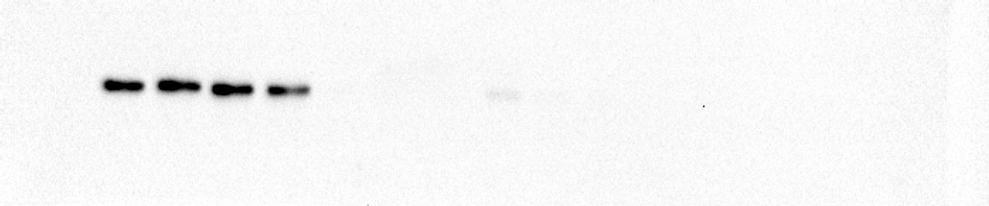

Supplement: Figure 2—figure supplement 1—source data 1. [file elife-98009-fig2-figsupp1-data1.zip › Figure 2-figure supplement 1-source data 1/Fig2-fig supp 1C-pTyr.tif]

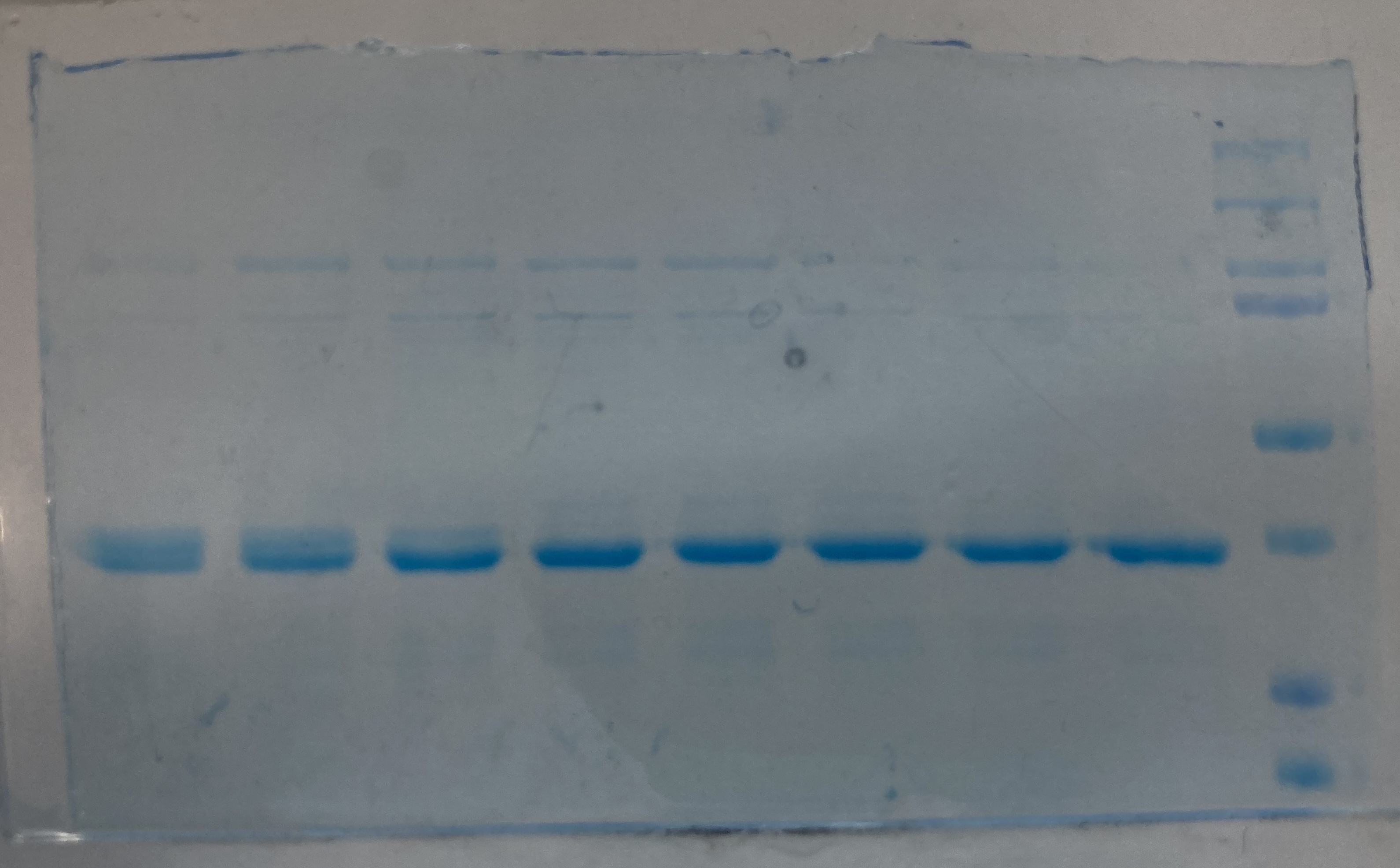

Supplement: Figure 2—figure supplement 1—source data 1. [file elife-98009-fig2-figsupp1-data1.zip › Figure 2-figure supplement 1-source data 1/Fig2-fig supp 1B-coomassie.tif]

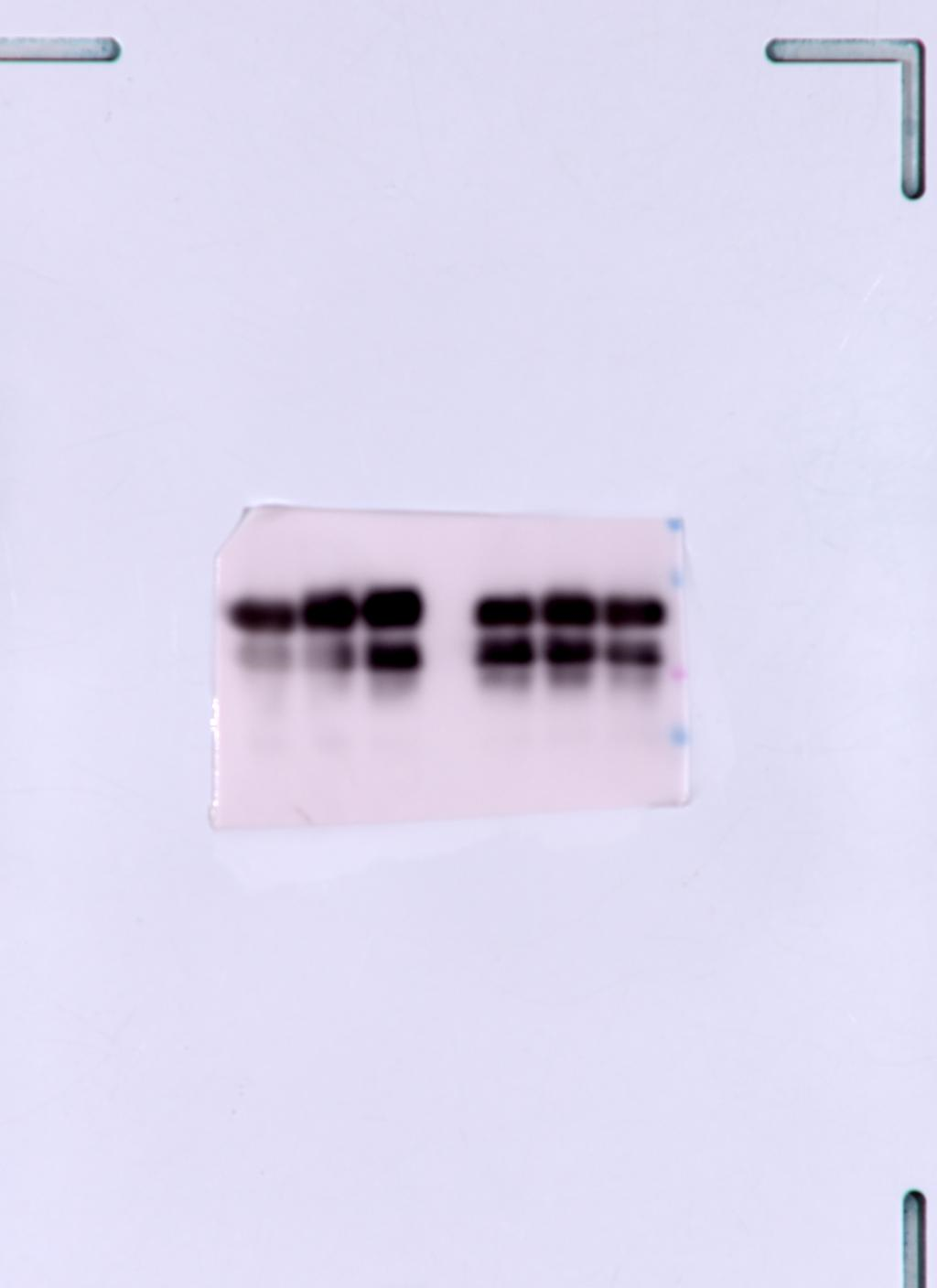

Supplement: Figure 2—figure supplement 1—source data 1. [file elife-98009-fig2-figsupp1-data1.zip › Figure 2-figure supplement 1-source data 1/Fig2-fig supp 1D-gst.tif]

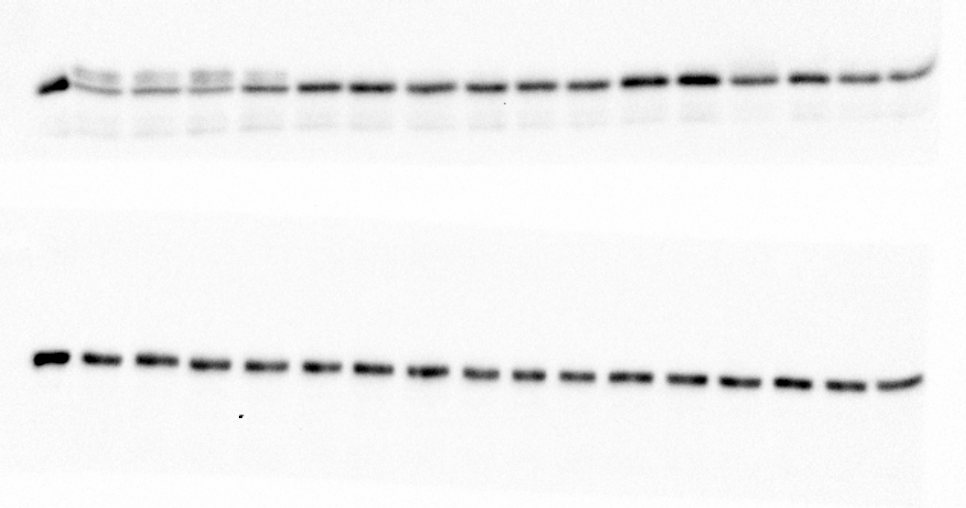

Supplement: Figure 2—figure supplement 1—source data 1. [file elife-98009-fig2-figsupp1-data1.zip › Figure 2-figure supplement 1-source data 1/Fig2-fig supp 1C-GST and IKK.tif]

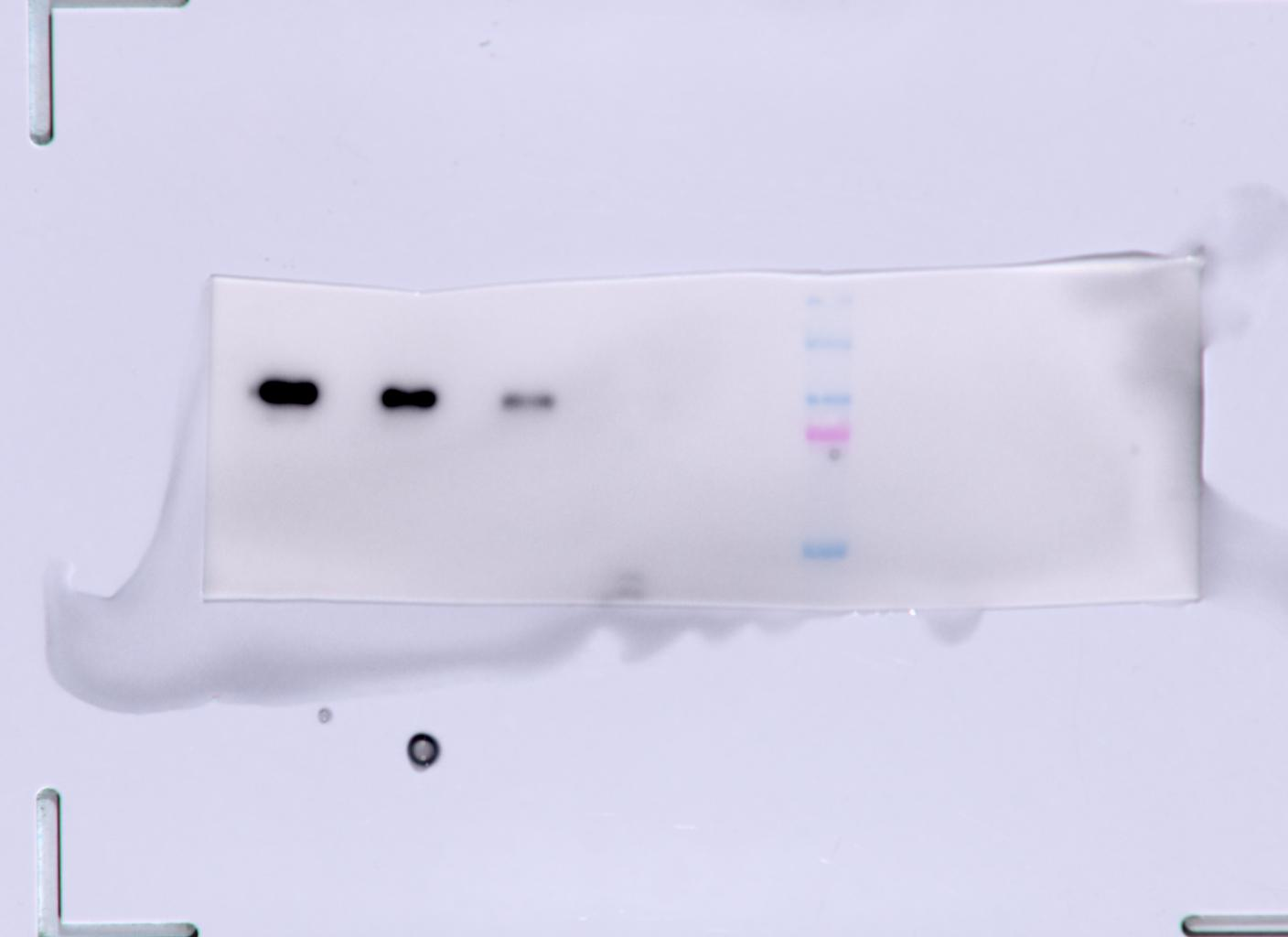

Supplement: Figure 2—figure supplement 1—source data 1. [file elife-98009-fig2-figsupp1-data1.zip › Figure 2-figure supplement 1-source data 1/Fig2-fig supp 1A-pTyr.tif]

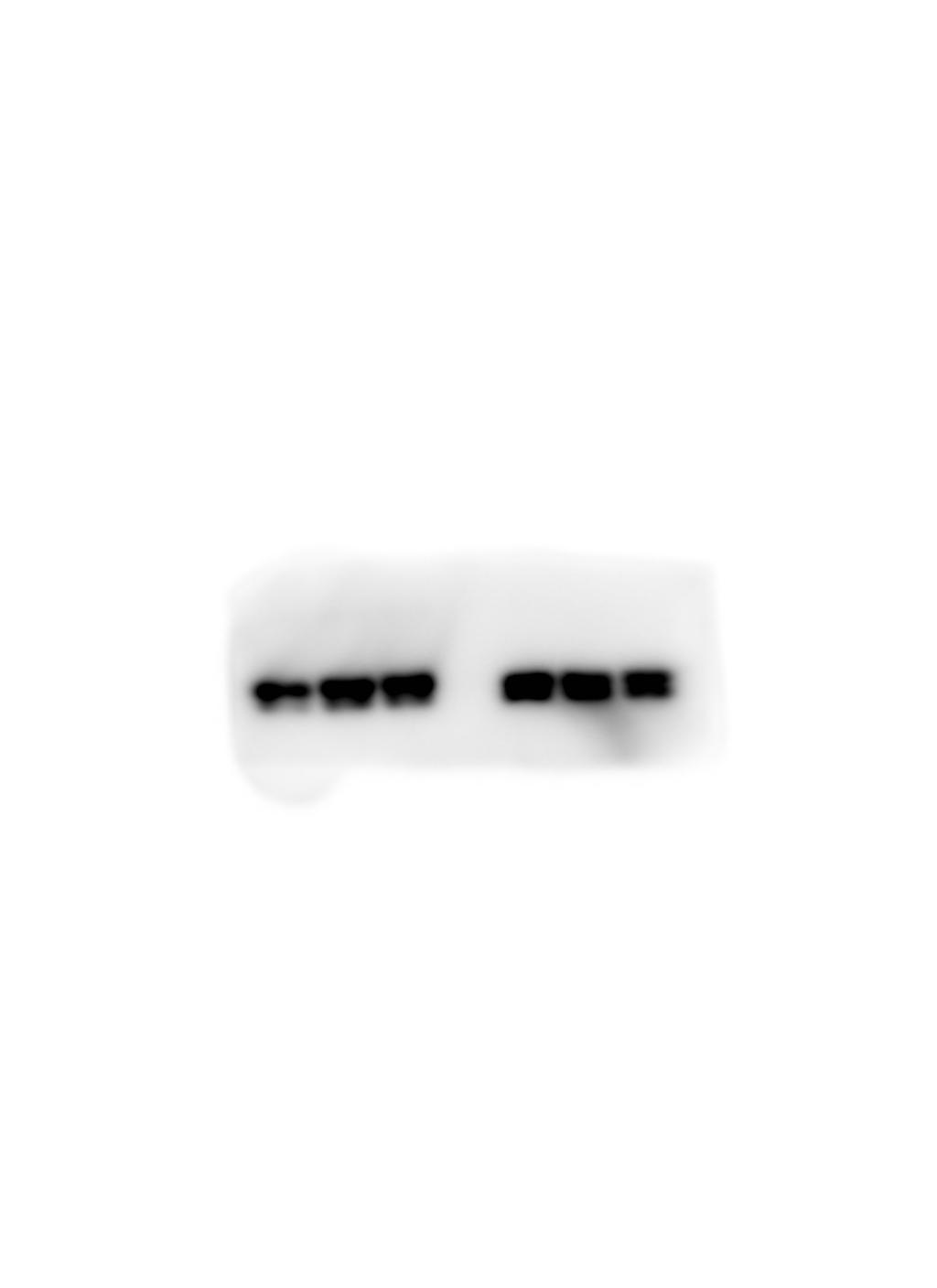

Supplement: Figure 2—figure supplement 1—source data 1. [file elife-98009-fig2-figsupp1-data1.zip › Figure 2-figure supplement 1-source data 1/Fig2-fig supp 1D-IKK2.tif]

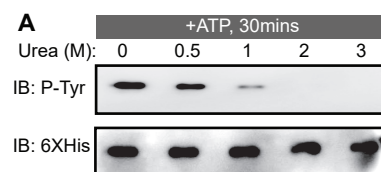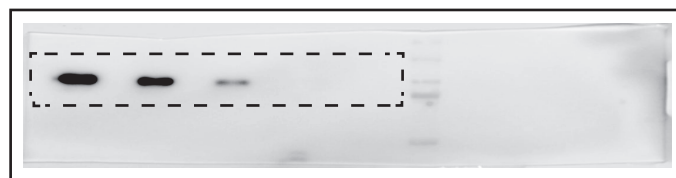

IB: P-Tyr

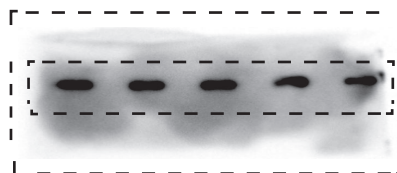

IB: 6XHis

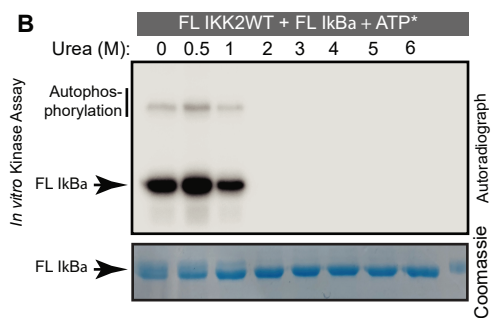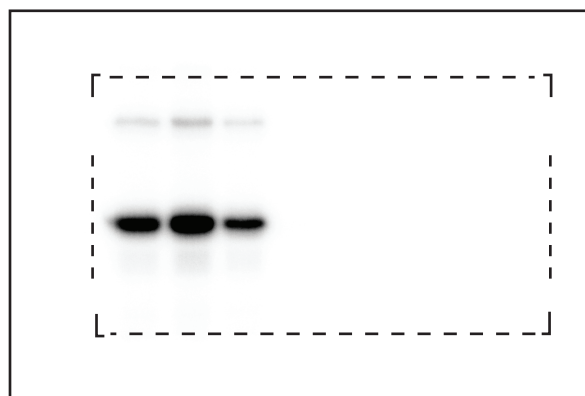

Autoradiograph

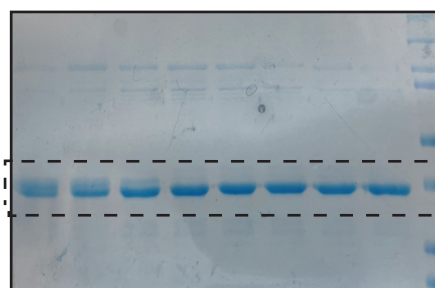

Coomassie

Supplement: Figure 2—figure supplement 1—source data 2. [file elife-98009-fig2-figsupp1-data2.zip › Figure 2-figure supplement 1-source data 2/Fig2-figure supplement 1A and 1B.pdf]

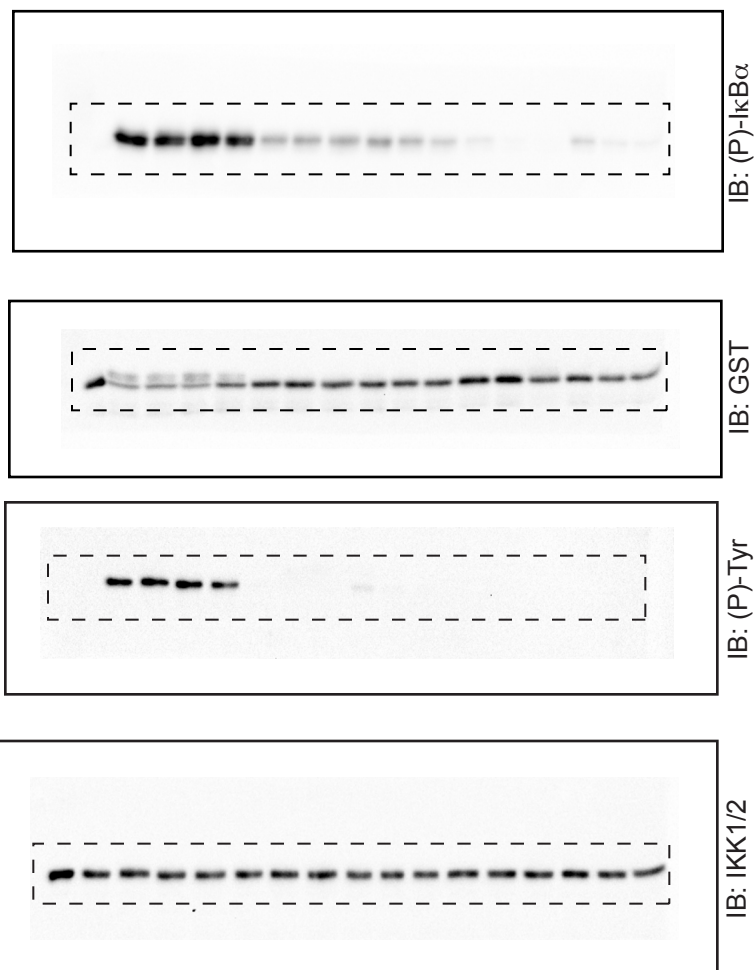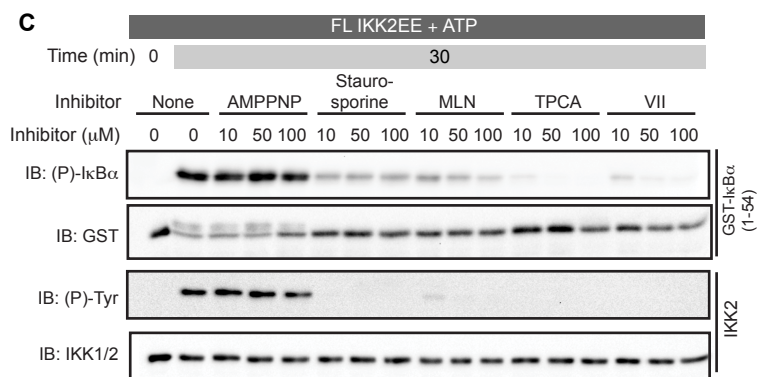

Supplement: Figure 2—figure supplement 1—source data 2. [file elife-98009-fig2-figsupp1-data2.zip › Figure 2-figure supplement 1-source data 2/Fig2-figure supplement 1C.pdf]

**D**

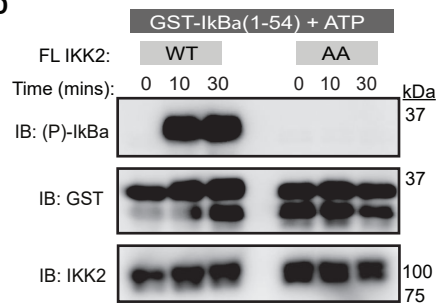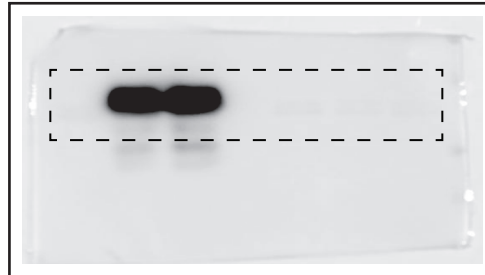

IB: (P)-IkBa

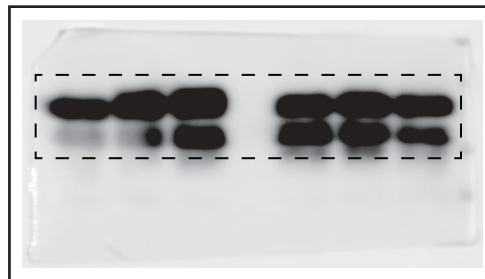

IB: GST

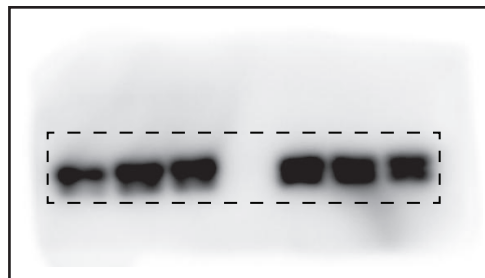

IB: IKK2

Supplement: Figure 2—figure supplement 1—source data 2. [file elife-98009-fig2-figsupp1-data2.zip › Figure 2-figure supplement 1-source data 2/Fig2-figure supplement 1D.pdf]

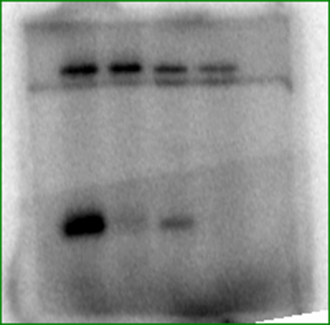

Supplement: Figure 3—source data 1. [file elife-98009-fig3-data1.zip › Figure 3-source data 1/Fig3E-KA-autorad-gray.tif]

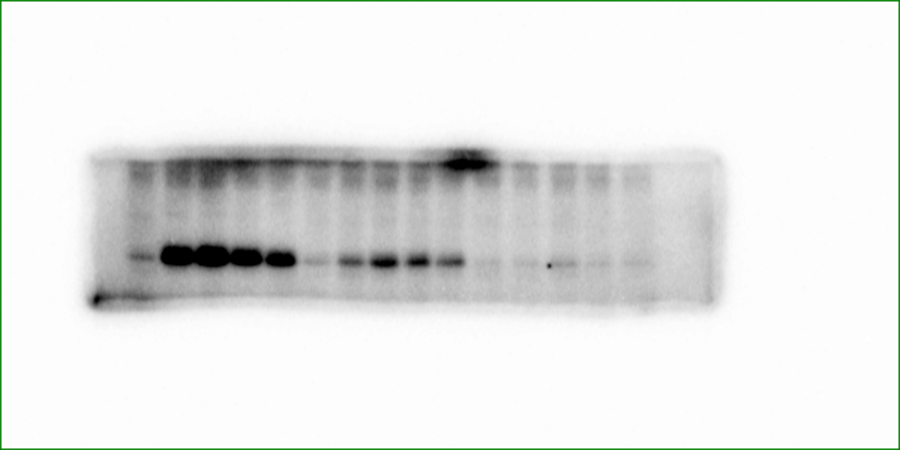

Supplement: Figure 3—source data 1. [file elife-98009-fig3-data1.zip › Figure 3-source data 1/Fig3G-autorad.tif]

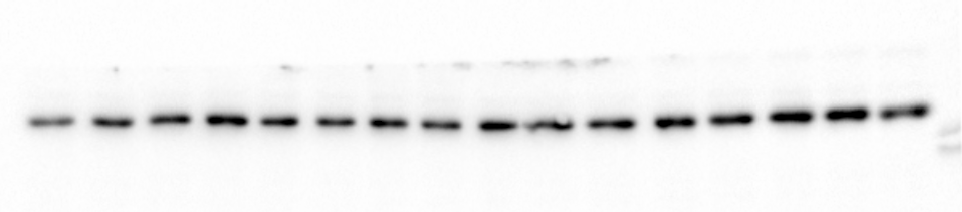

Supplement: Figure 3—source data 1. [file elife-98009-fig3-data1.zip › Figure 3-source data 1/Fig3G-Tubulin.tif]

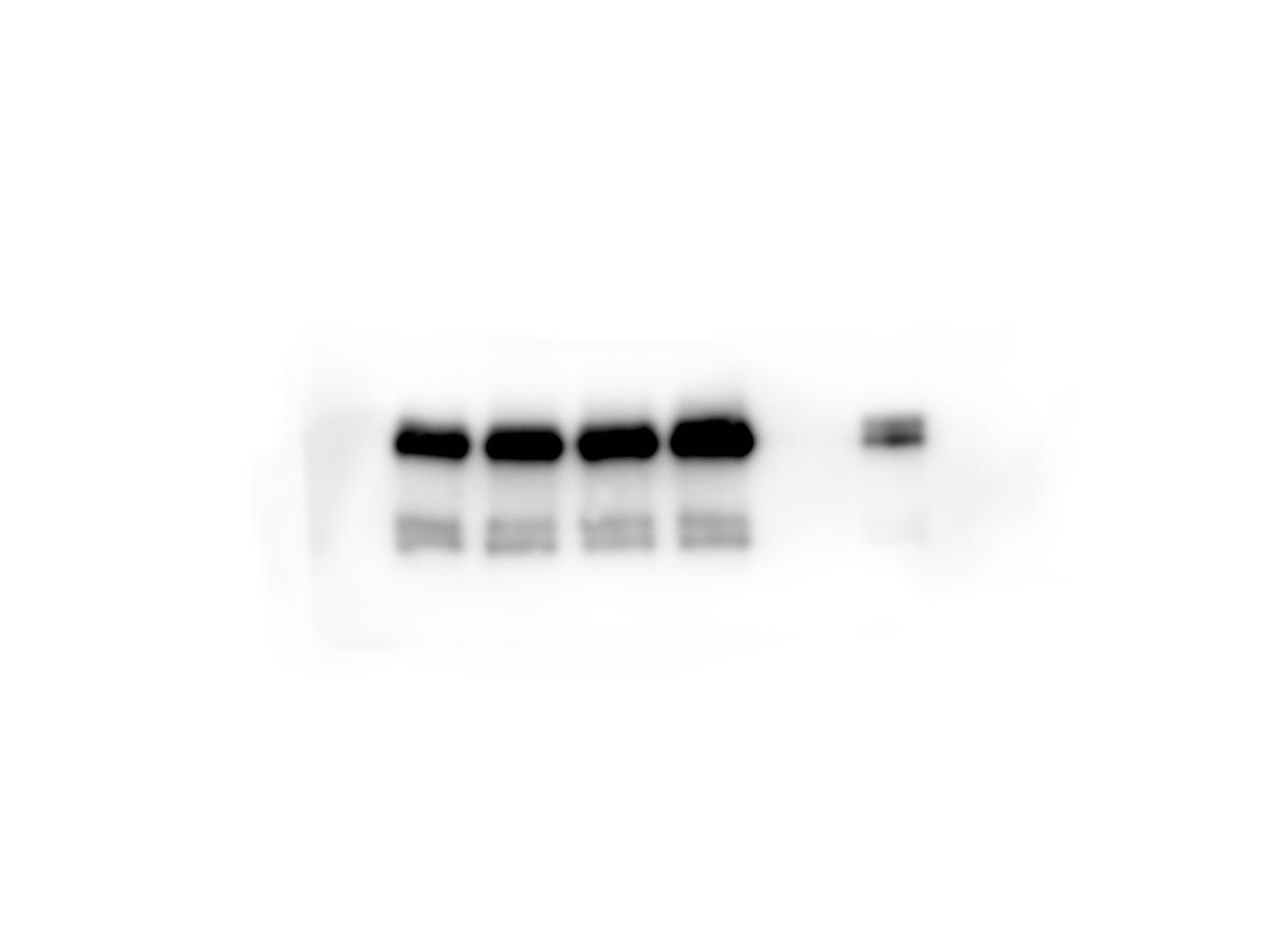

Supplement: Figure 3—source data 1. [file elife-98009-fig3-data1.zip › Figure 3-source data 1/Fig3D-IkBa.tif]

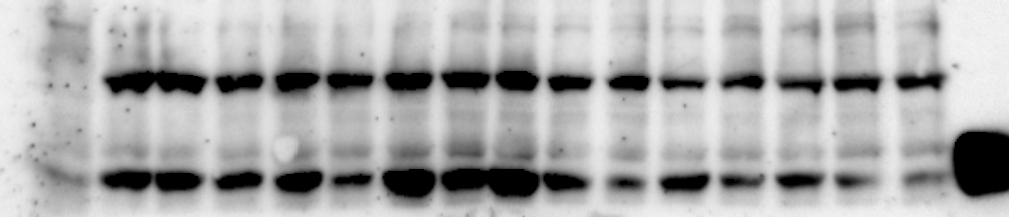

Supplement: Figure 3—source data 1. [file elife-98009-fig3-data1.zip › Figure 3-source data 1/Fig3G-HA.tif]

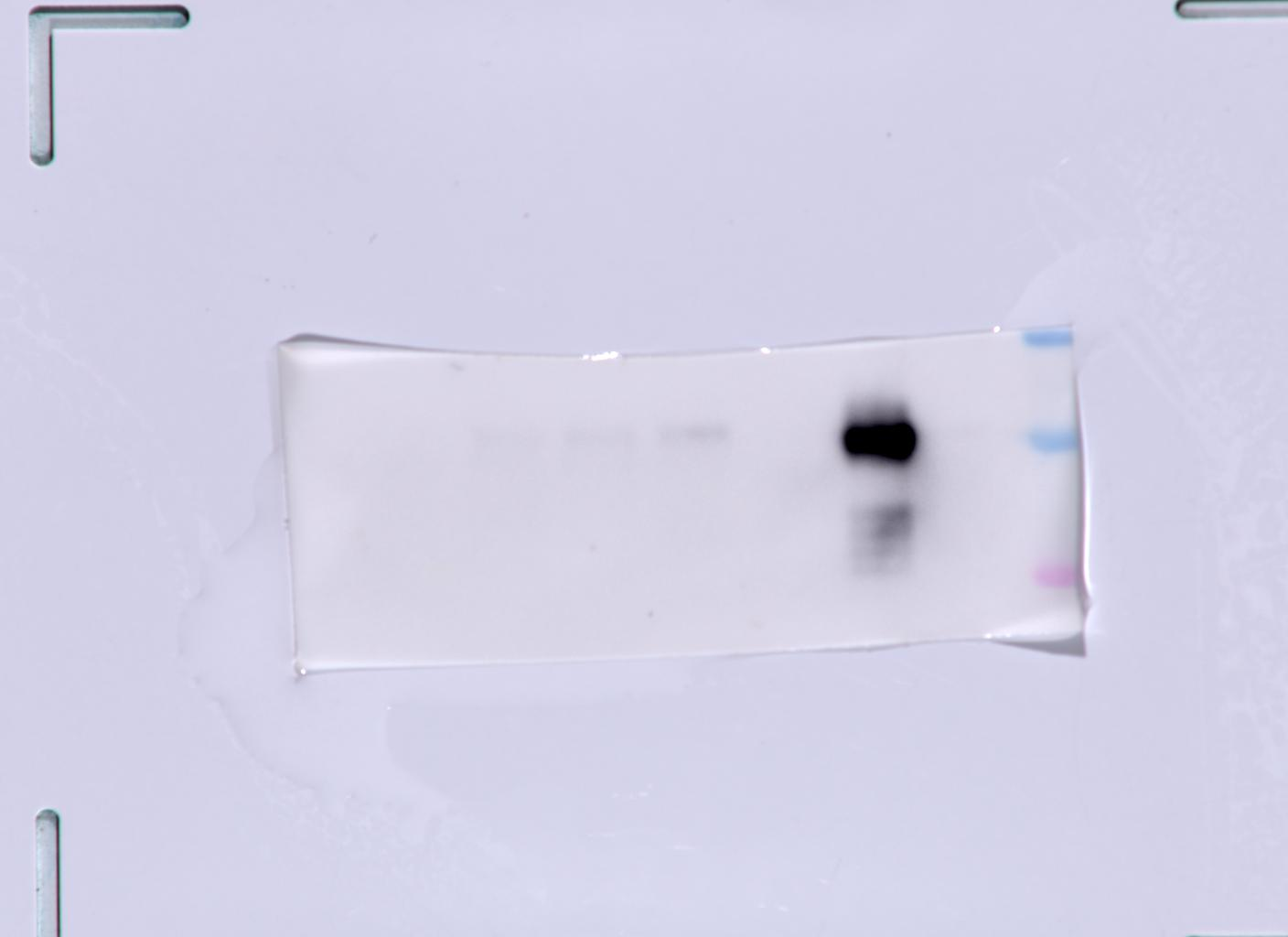

Supplement: Figure 3—source data 1. [file elife-98009-fig3-data1.zip › Figure 3-source data 1/Fig3D-pIkBa.tif]

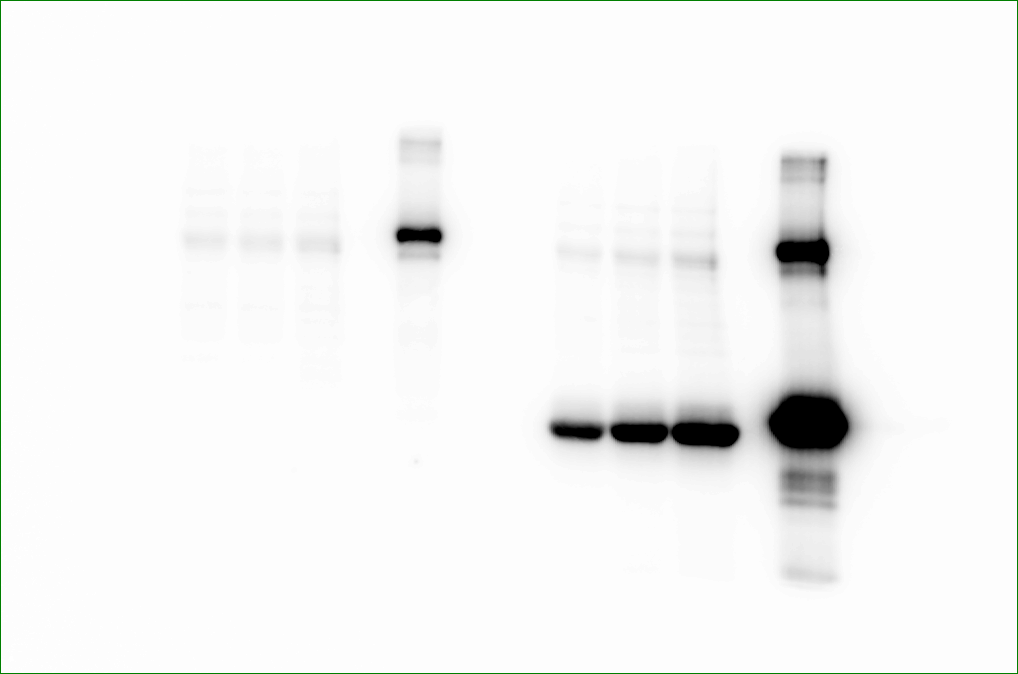

Supplement: Figure 3—source data 1. [file elife-98009-fig3-data1.zip › Figure 3-source data 1/Fig3C-autorad.tif]

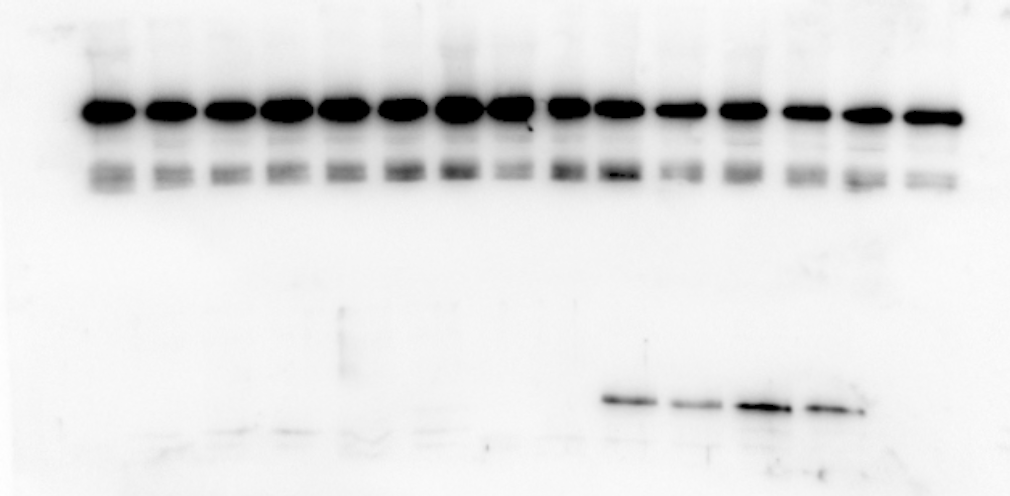

Supplement: Figure 3—source data 1. [file elife-98009-fig3-data1.zip › Figure 3-source data 1/Fig3G-IKK.tif]

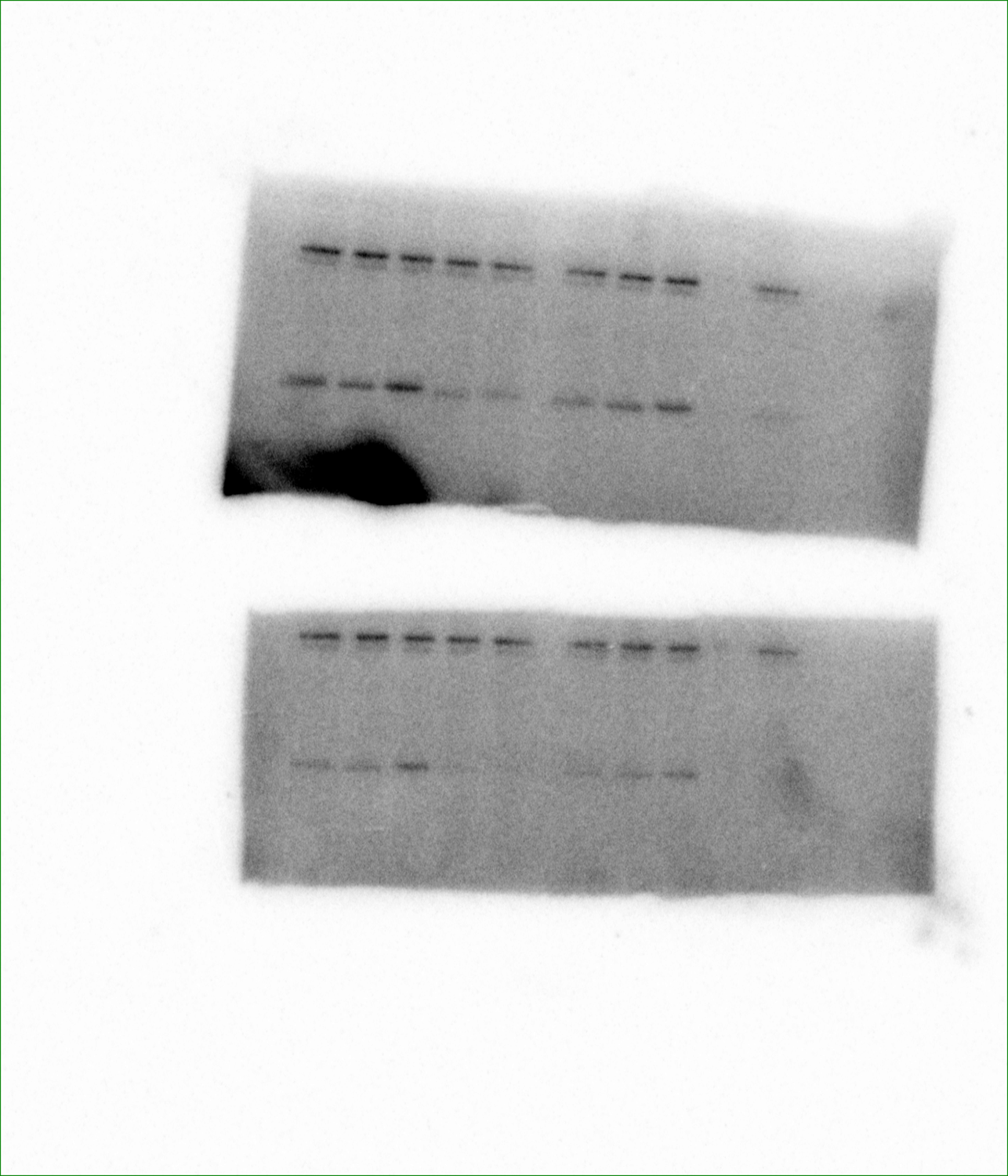

Supplement: Figure 3—source data 1. [file elife-98009-fig3-data1.zip › Figure 3-source data 1/Fig3F-autorad.tif]

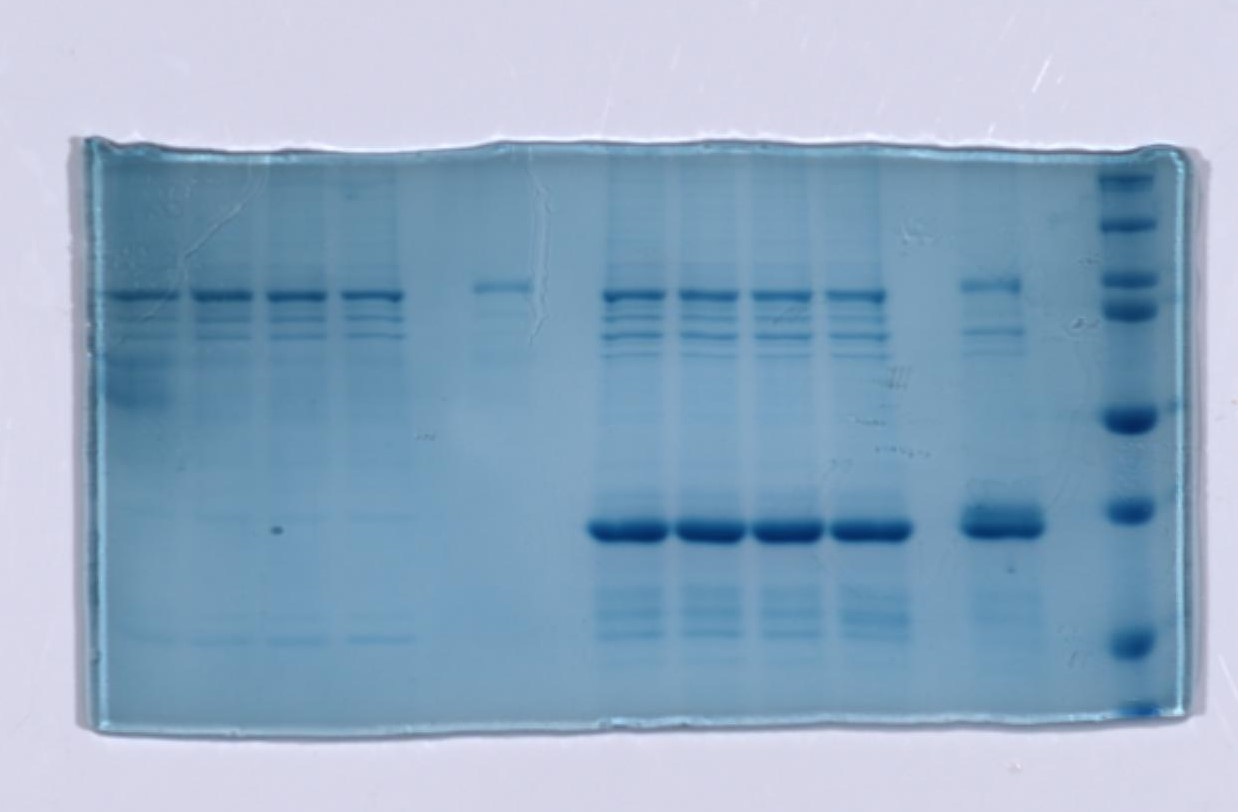

Supplement: Figure 3—source data 1. [file elife-98009-fig3-data1.zip › Figure 3-source data 1/Fig3C-coomassie.tif]

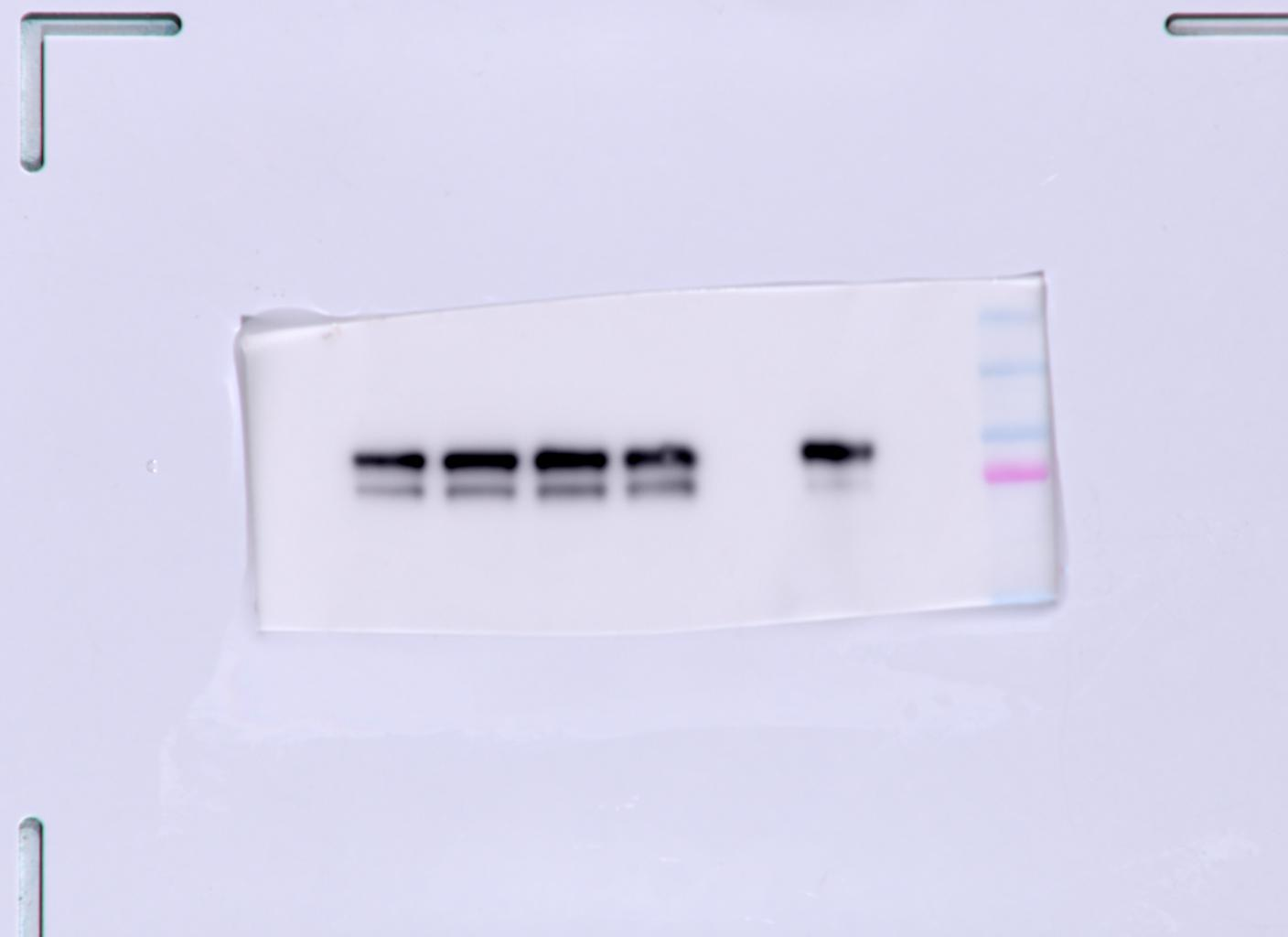

Supplement: Figure 3—source data 1. [file elife-98009-fig3-data1.zip › Figure 3-source data 1/Fig3D-IKK2.tif]

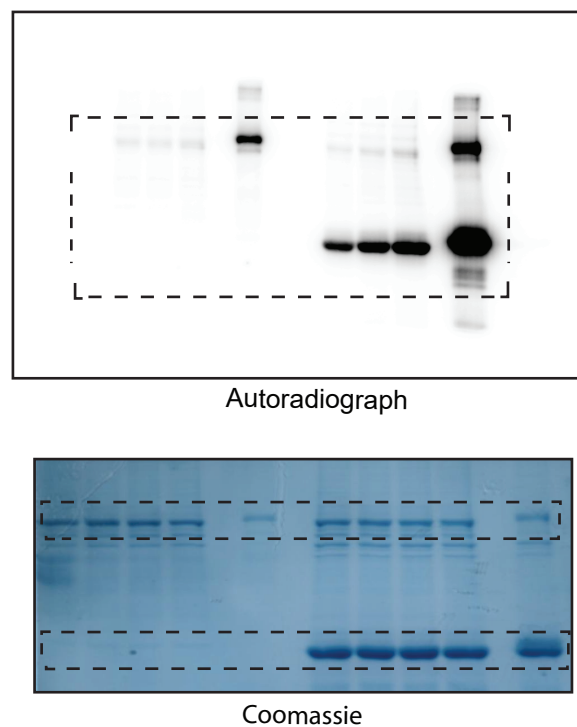

Coomassie

Supplement: Figure 3—source data 2. [file elife-98009-fig3-data2.zip › Figure 3-source data 2/Fig3C.pdf]

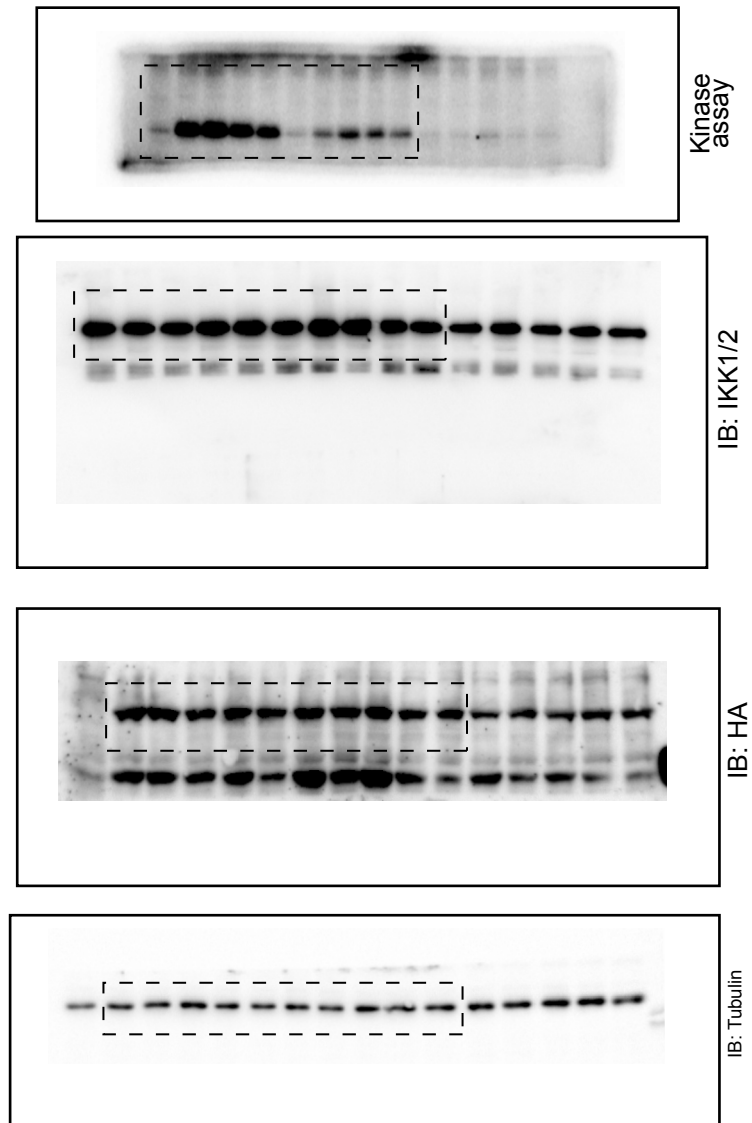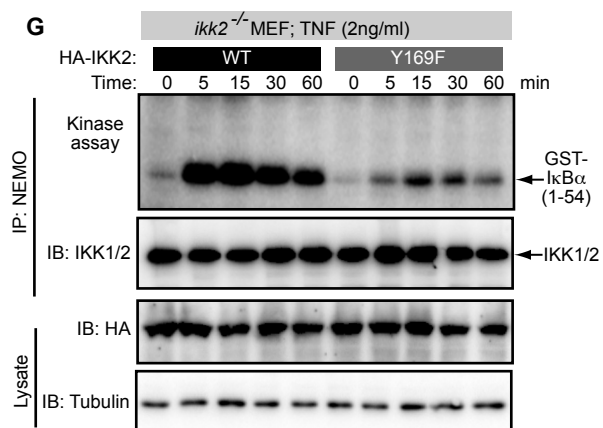

Supplement: Figure 3—source data 2. [file elife-98009-fig3-data2.zip › Figure 3-source data 2/Fig3G.pdf]

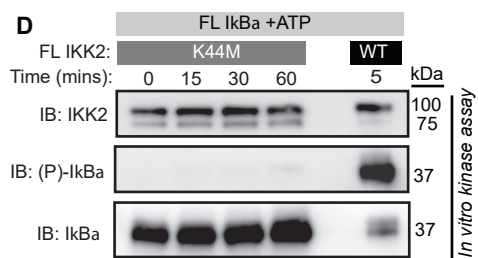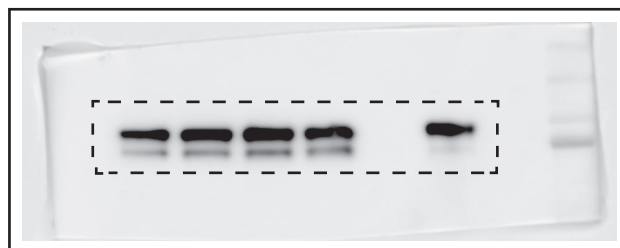

IB: IKK2

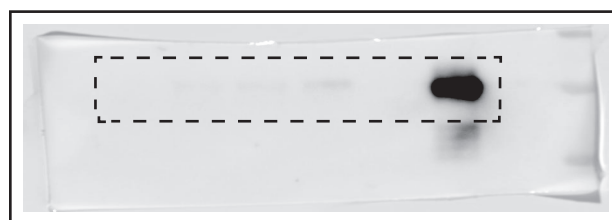

IB: (P)-IkBa

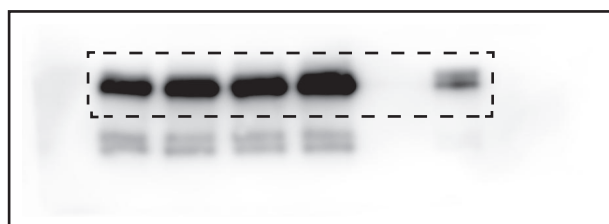

IB: IkBa

Supplement: Figure 3—source data 2. [file elife-98009-fig3-data2.zip › Figure 3-source data 2/Fig3D.pdf]

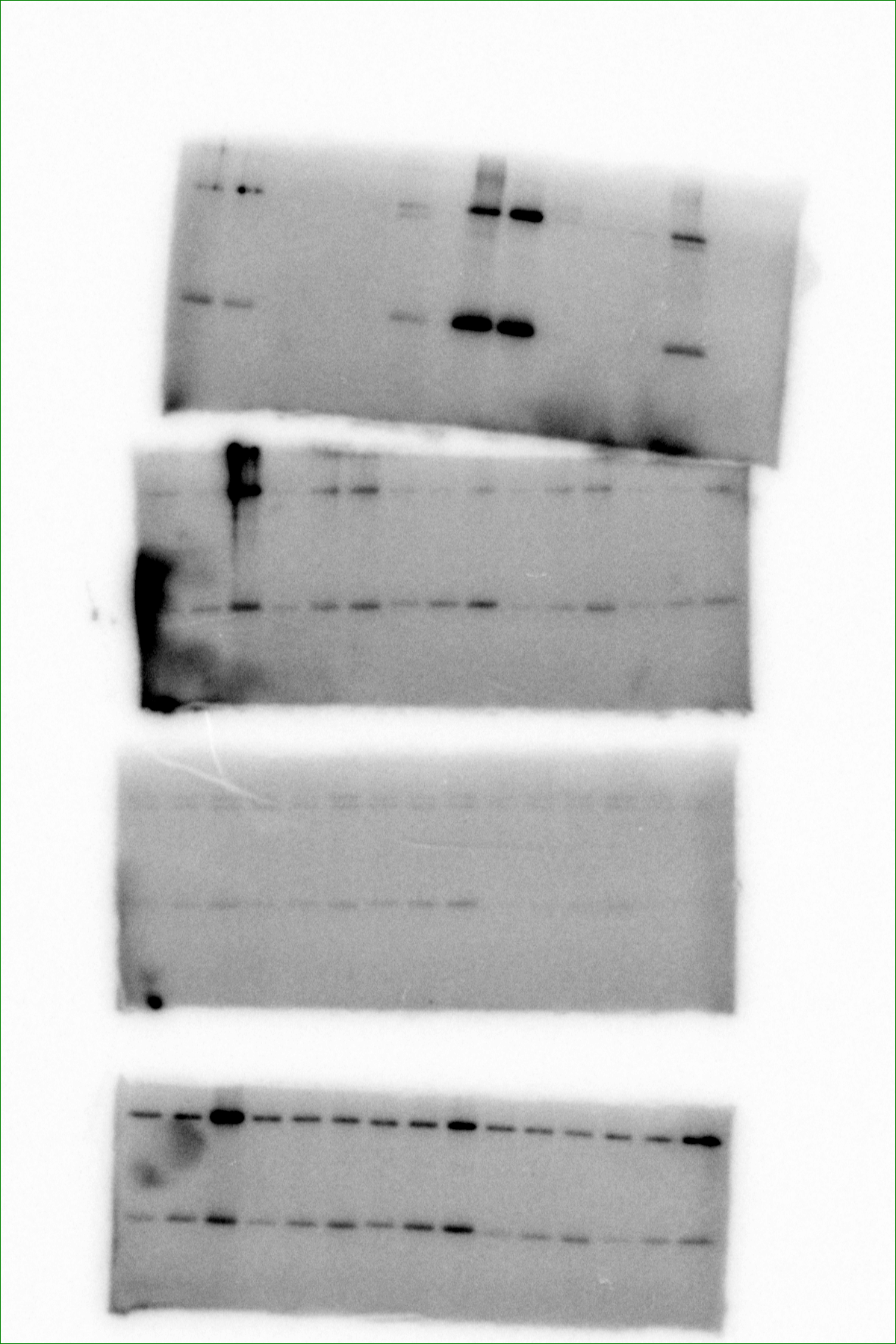

Supplement: Figure 3—figure supplement 1—source data 1. [file elife-98009-fig3-figsupp1-data1.zip › Figure 3-figure supplement 1-source data 1/Fig3-fig supp 1F-wt and muts IKK2.tif]

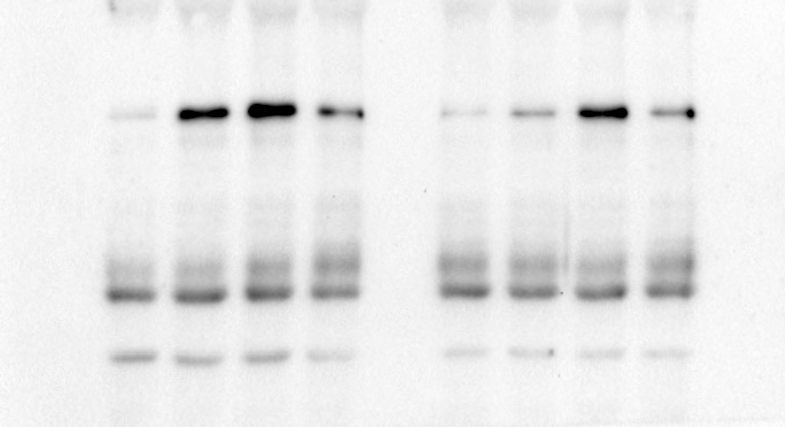

Supplement: Figure 3—figure supplement 1—source data 1. [file elife-98009-fig3-figsupp1-data1.zip › Figure 3-figure supplement 1-source data 1/Fig3-fig supp 1G-(P)-Ser-IKK2.tif]

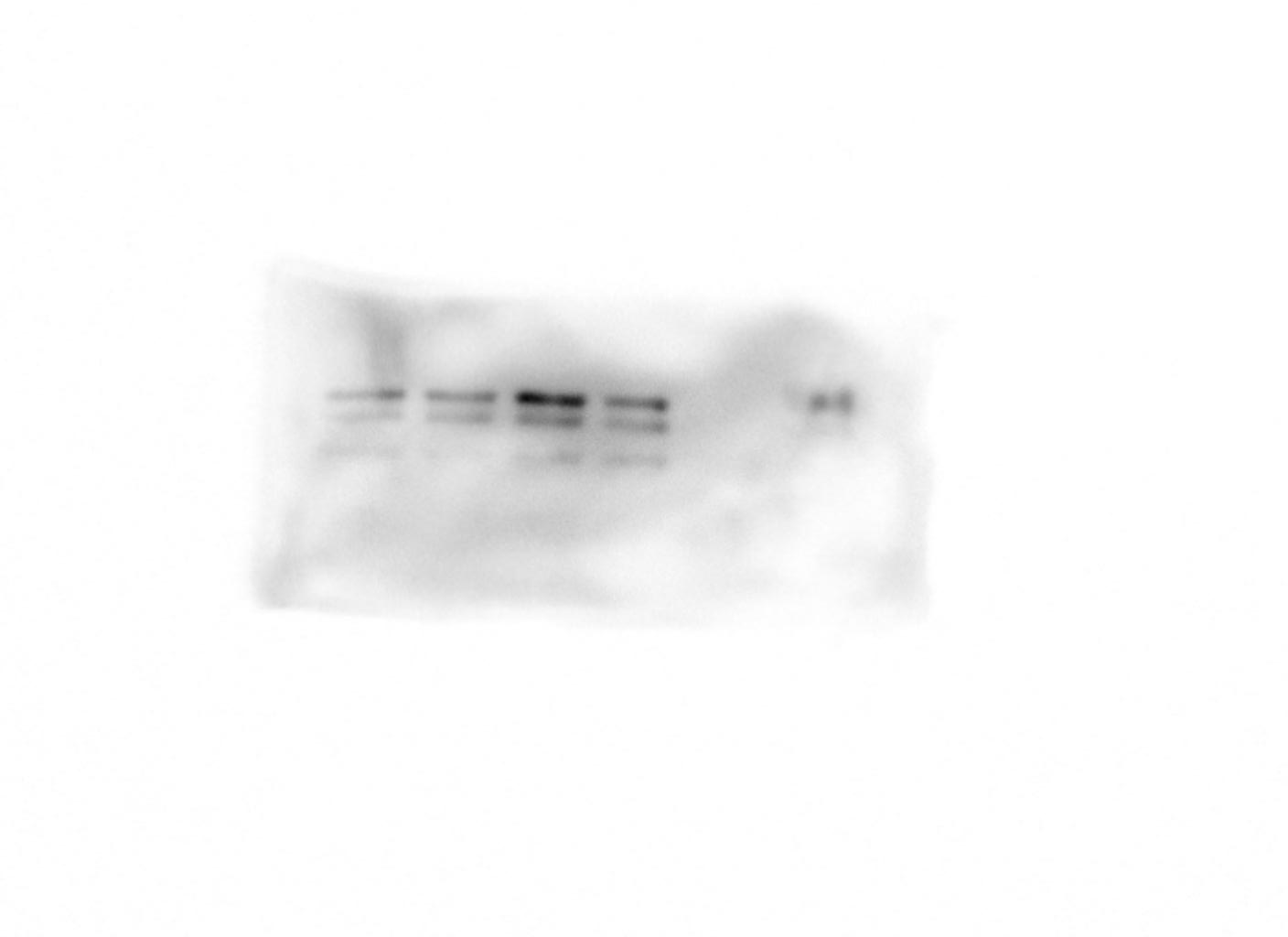

Supplement: Figure 3—figure supplement 1—source data 1. [file elife-98009-fig3-figsupp1-data1.zip › Figure 3-figure supplement 1-source data 1/Fig3-fig supp 1D-IKK2.tif]

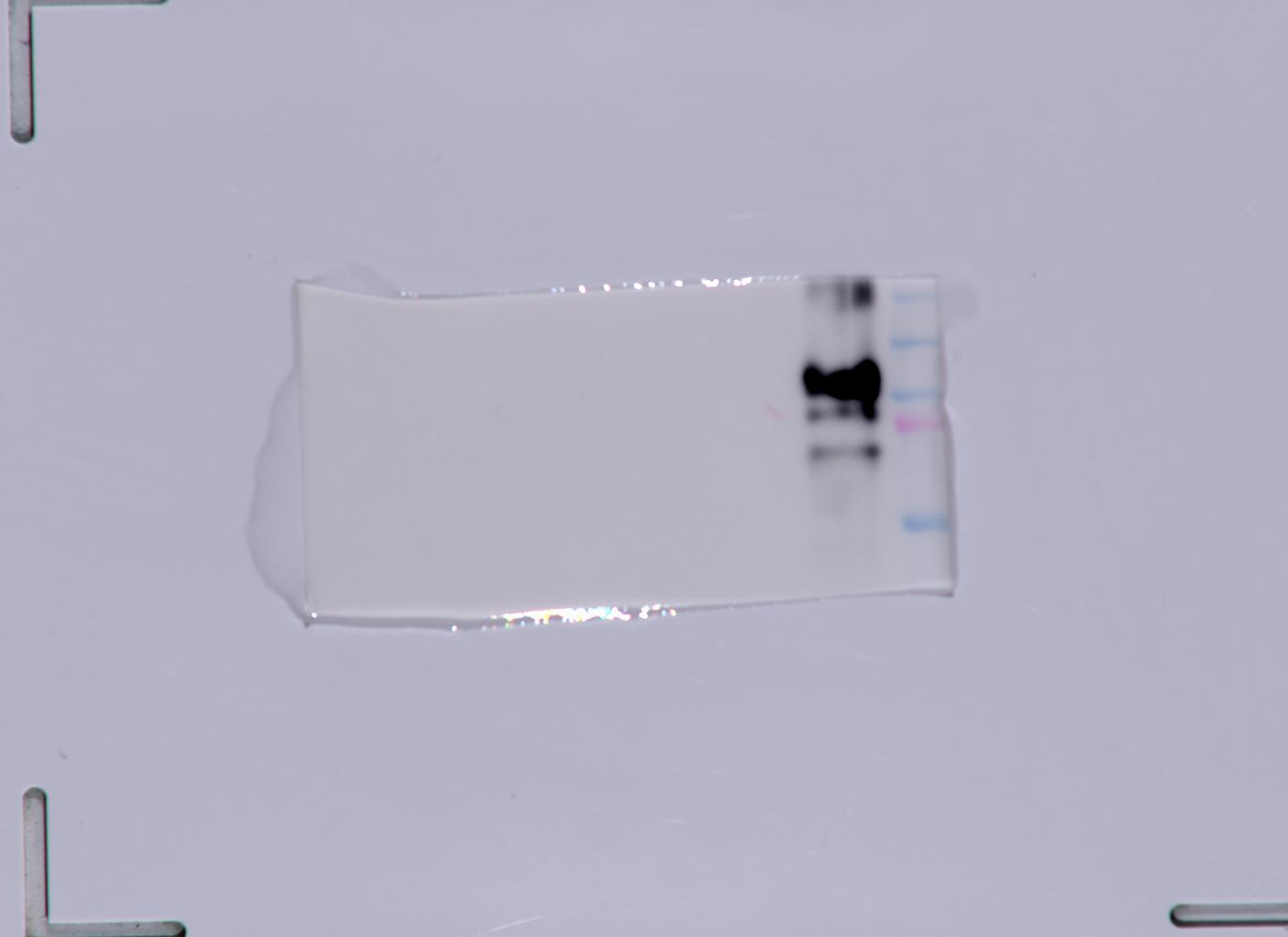

Supplement: Figure 3—figure supplement 1—source data 1. [file elife-98009-fig3-figsupp1-data1.zip › Figure 3-figure supplement 1-source data 1/Fig3-fig supp 1D-pSer.tif]

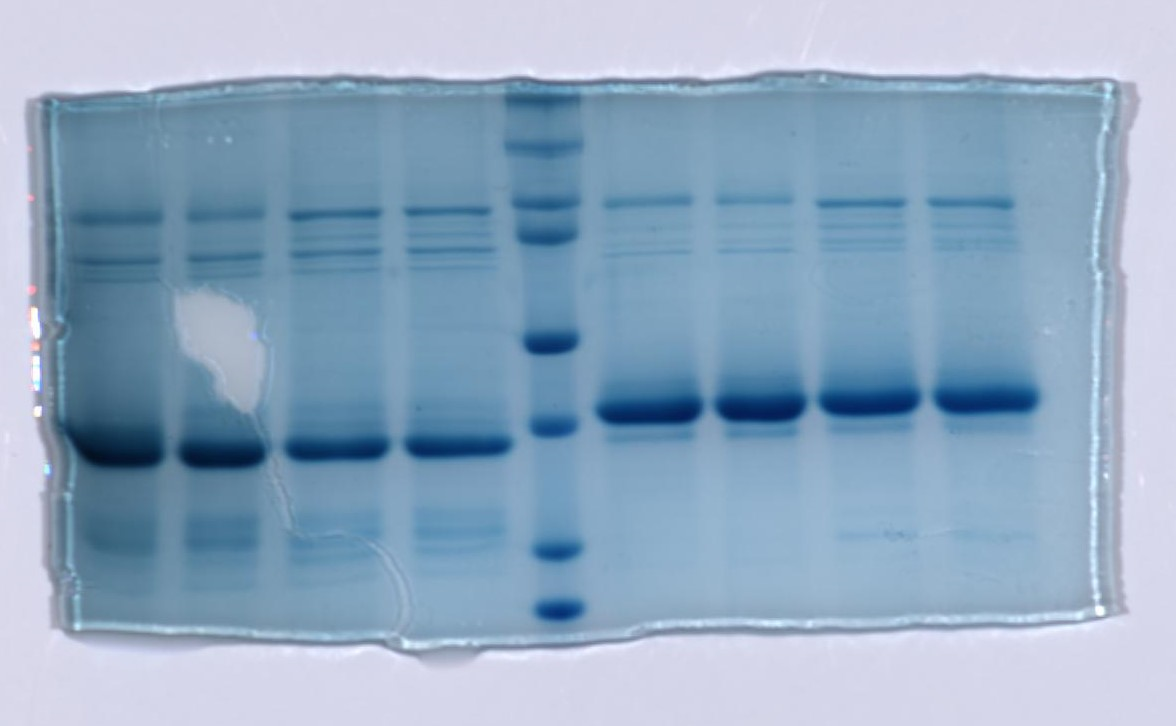

Supplement: Figure 3—figure supplement 1—source data 1. [file elife-98009-fig3-figsupp1-data1.zip › Figure 3-figure supplement 1-source data 1/Fig3-fig supp 1E-coomassie.tif]

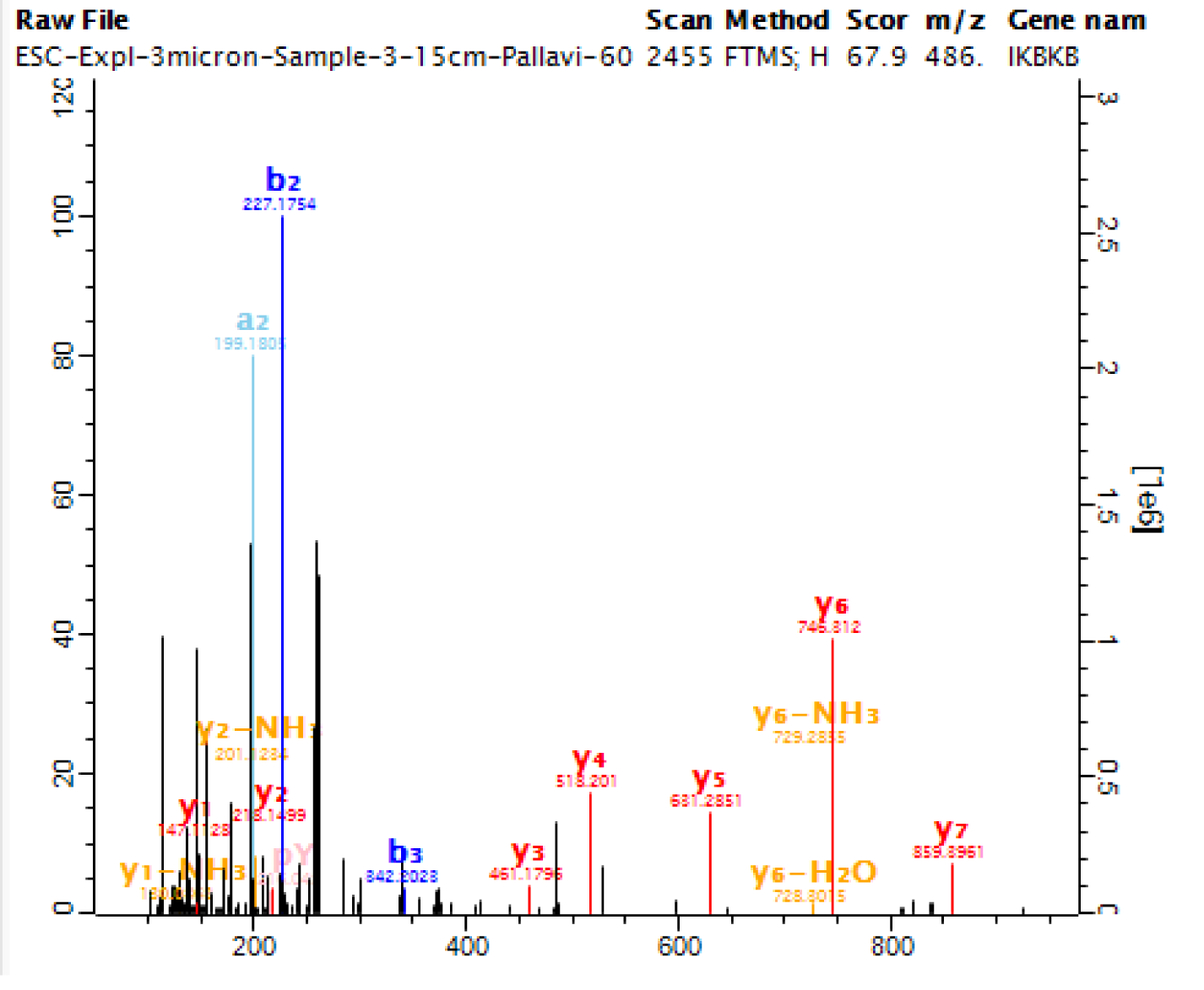

Supplement: Figure 3—figure supplement 1—source data 1. [file elife-98009-fig3-figsupp1-data1.zip › Figure 3-figure supplement 1-source data 1/Fig3-fig supp 1A.tif]

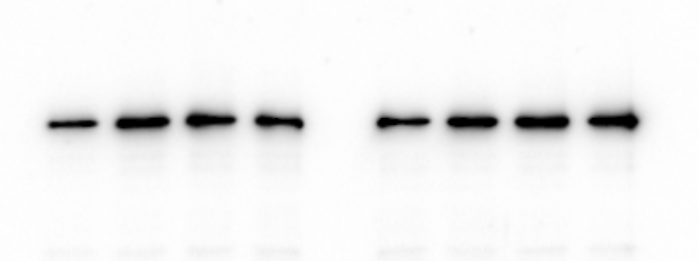

Supplement: Figure 3—figure supplement 1—source data 1. [file elife-98009-fig3-figsupp1-data1.zip › Figure 3-figure supplement 1-source data 1/Fig3-fig supp 1G-IKK.tif]

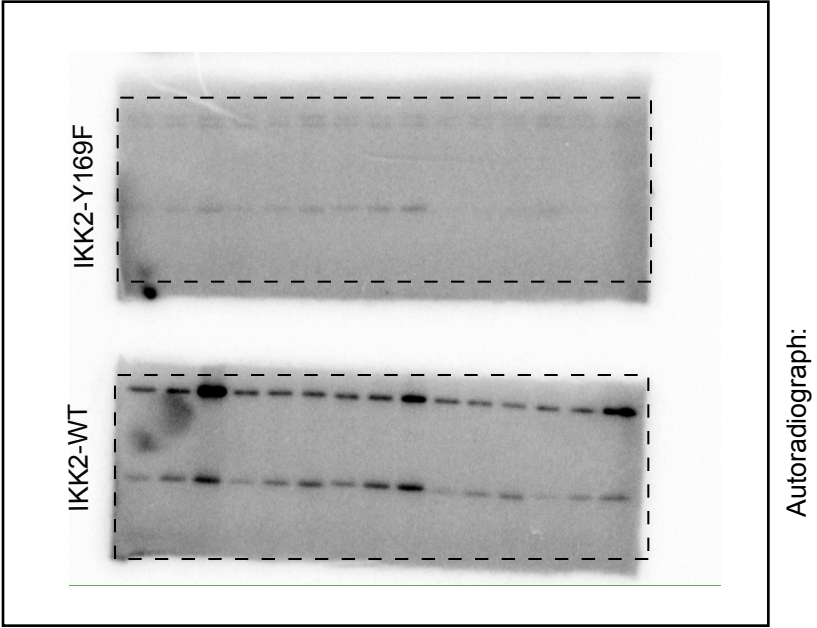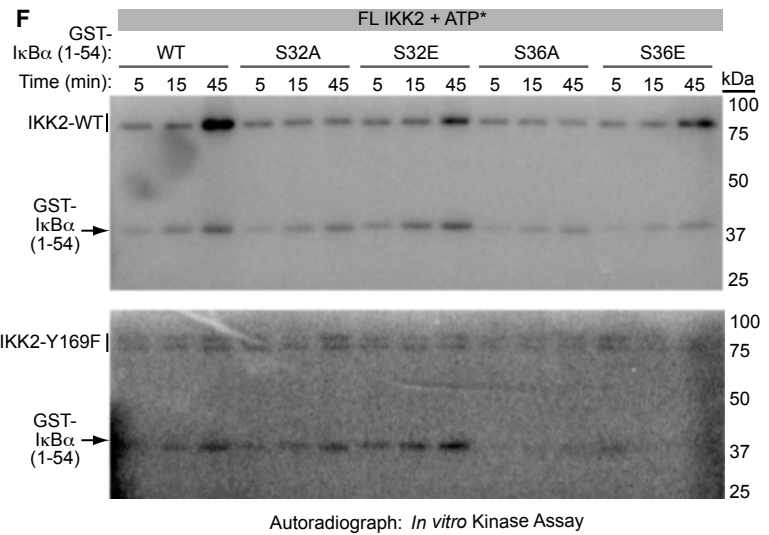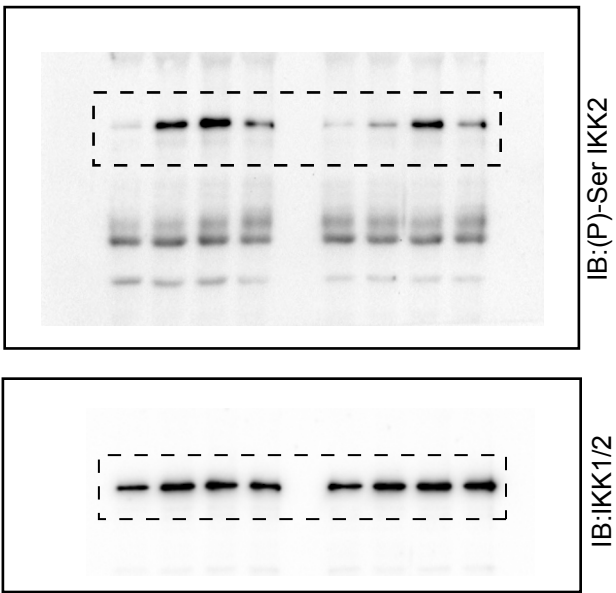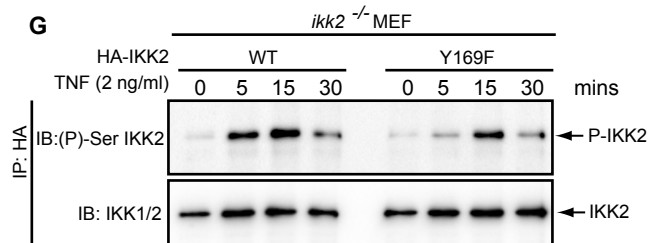

Supplement: Figure 3—figure supplement 1—source data 2. [file elife-98009-fig3-figsupp1-data2.zip › Figure 3-figure supplement 1-source data 2/Fig3-figure supplement 1F and 1G.pdf]

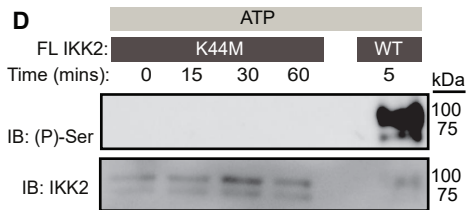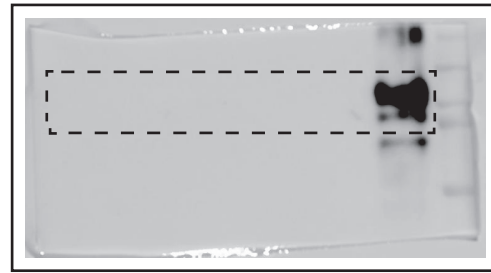

IB: (P)-Ser

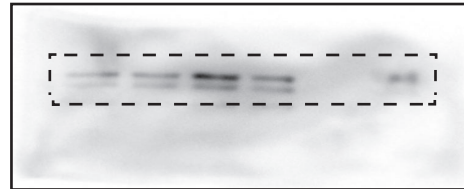

IB: IKK2

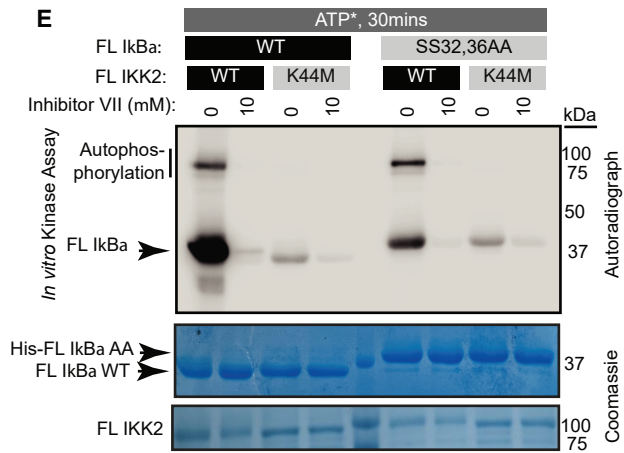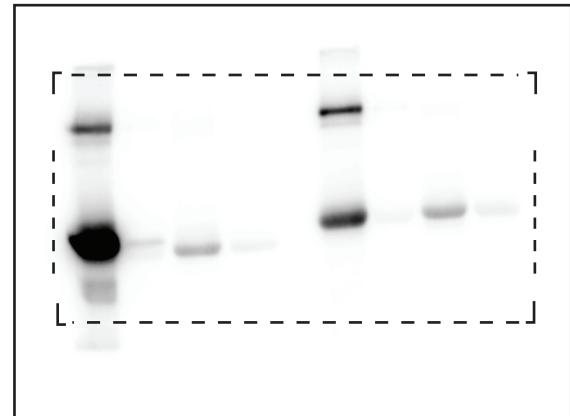

Autoradiograph

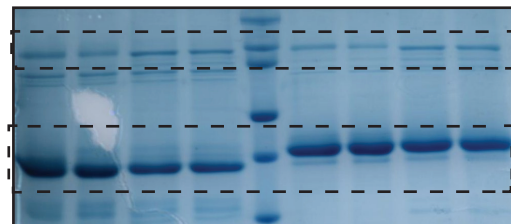

Coomassie

Supplement: Figure 3—figure supplement 1—source data 2. [file elife-98009-fig3-figsupp1-data2.zip › Figure 3-figure supplement 1-source data 2/Fig3-figure supplement 1D and 1E.pdf]

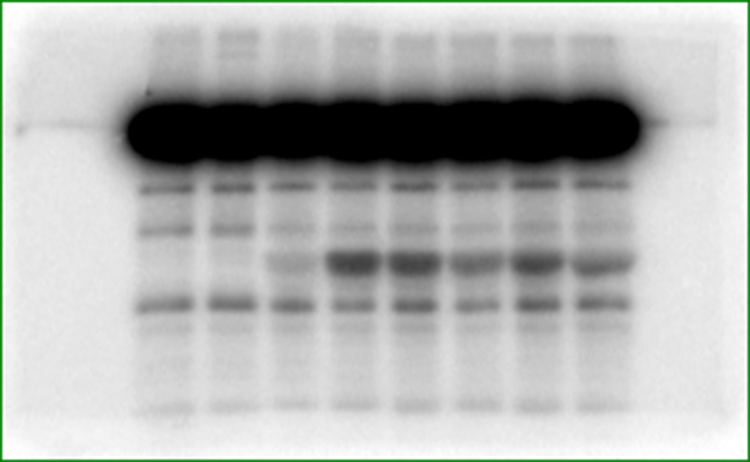

Supplement: Figure 5—source data 1. [file elife-98009-fig5-data1.zip › Figure 5-source data 1/Fig5B-autorad.tif]

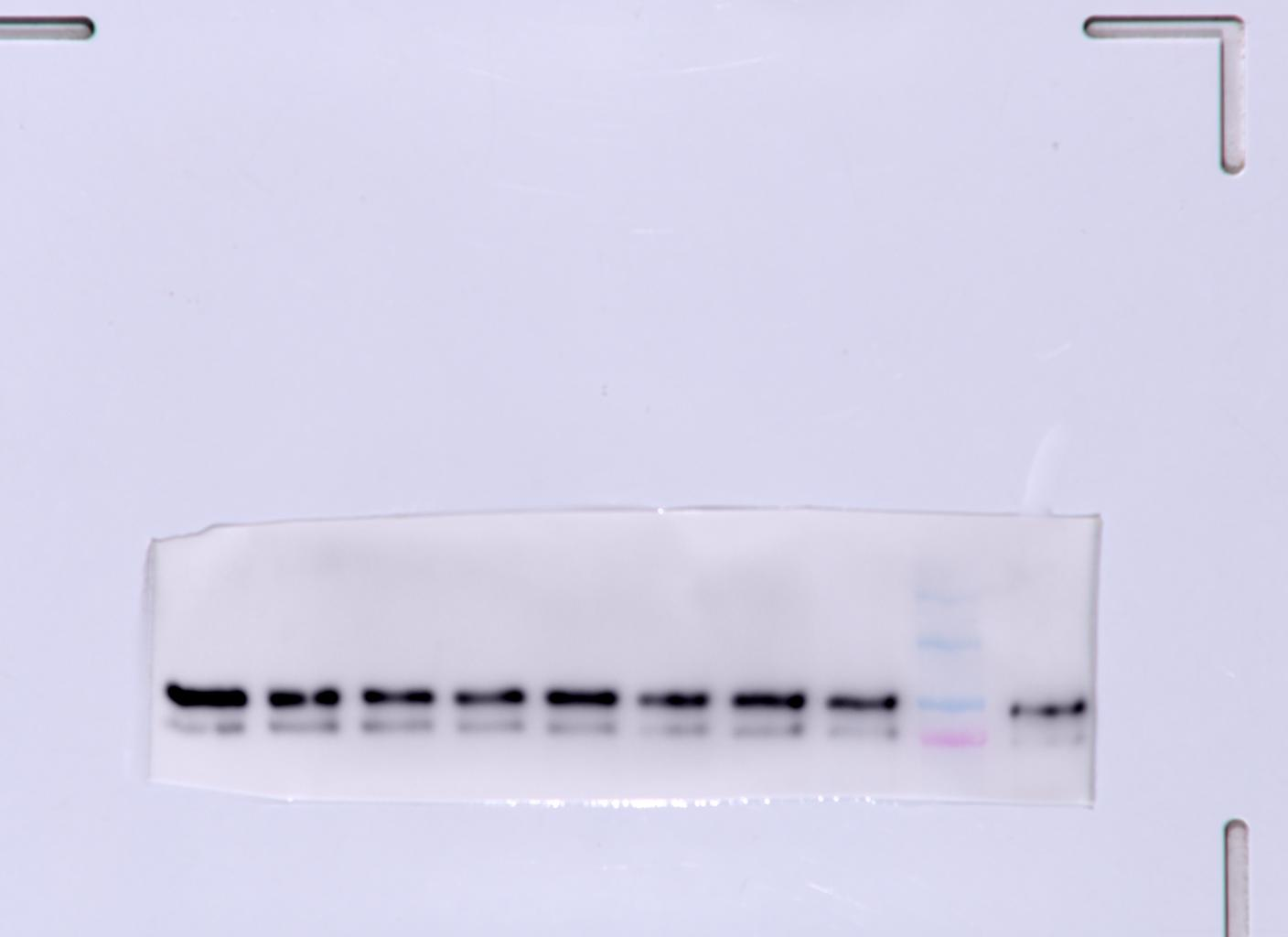

Supplement: Figure 5—source data 1. [file elife-98009-fig5-data1.zip › Figure 5-source data 1/Fig5E-pTyr.tif]

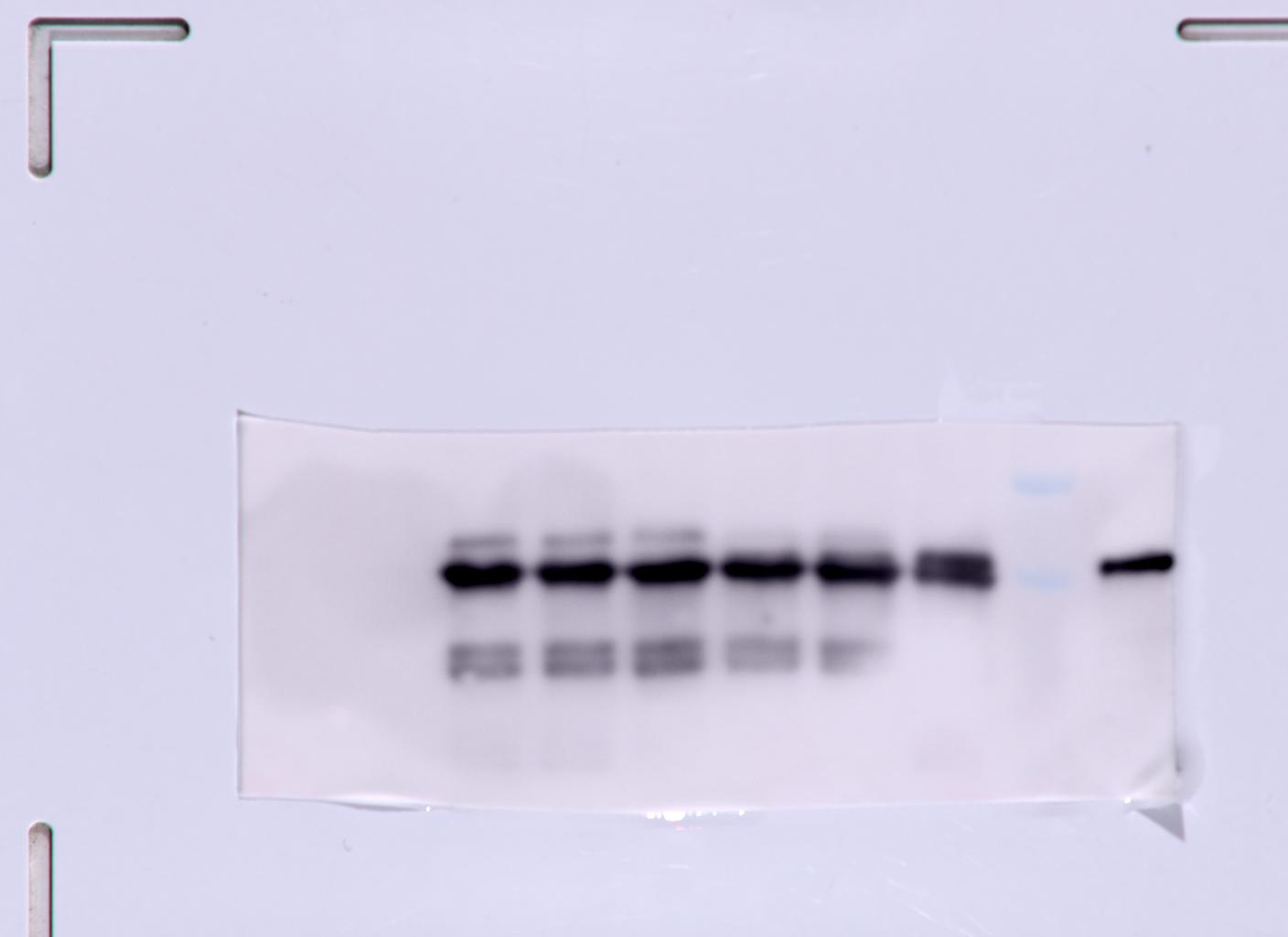

Supplement: Figure 5—source data 1. [file elife-98009-fig5-data1.zip › Figure 5-source data 1/Fig5E-IkBa.tif]

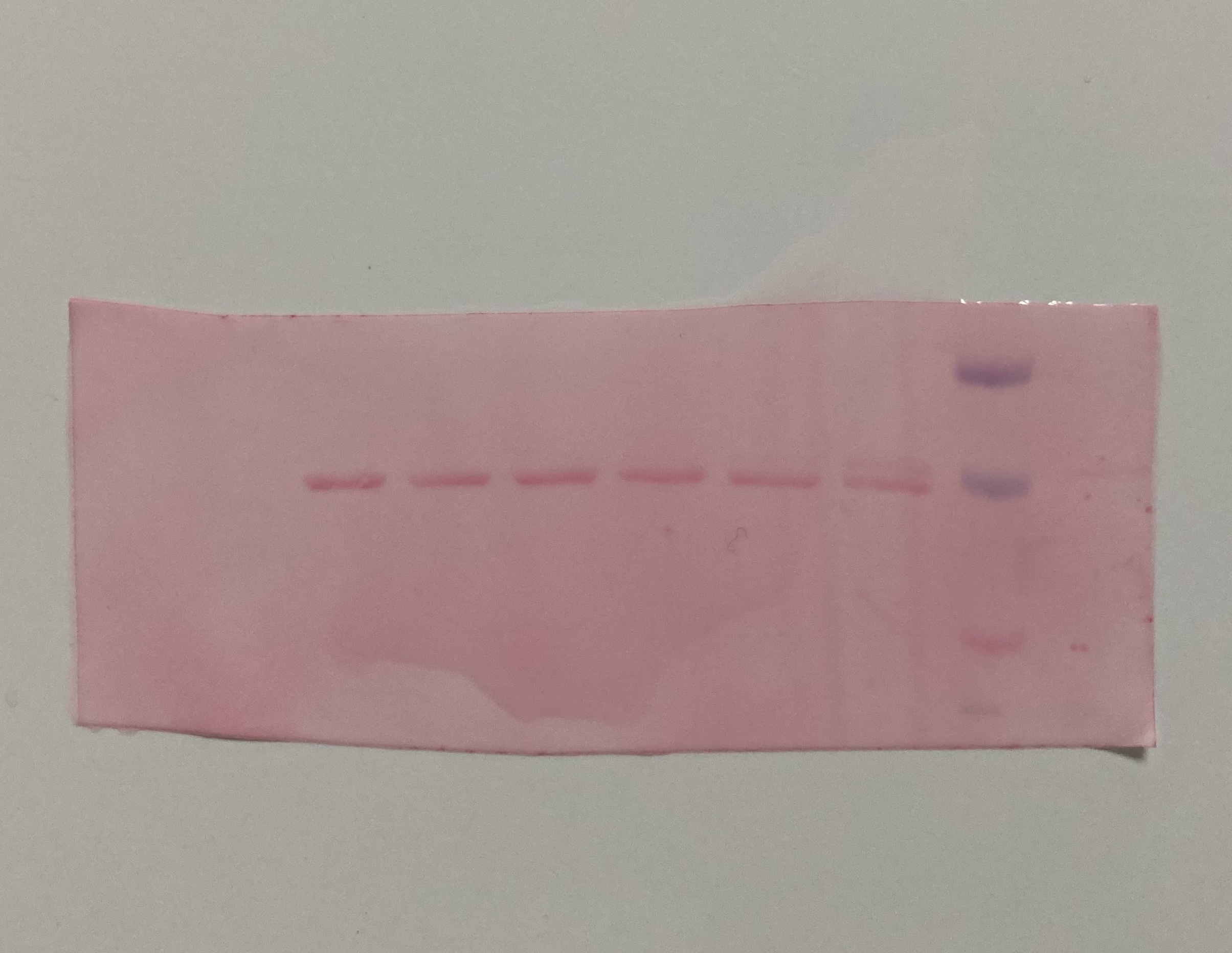

Supplement: Figure 5—source data 1. [file elife-98009-fig5-data1.zip › Figure 5-source data 1/Fig5E-Ponceau.tif]

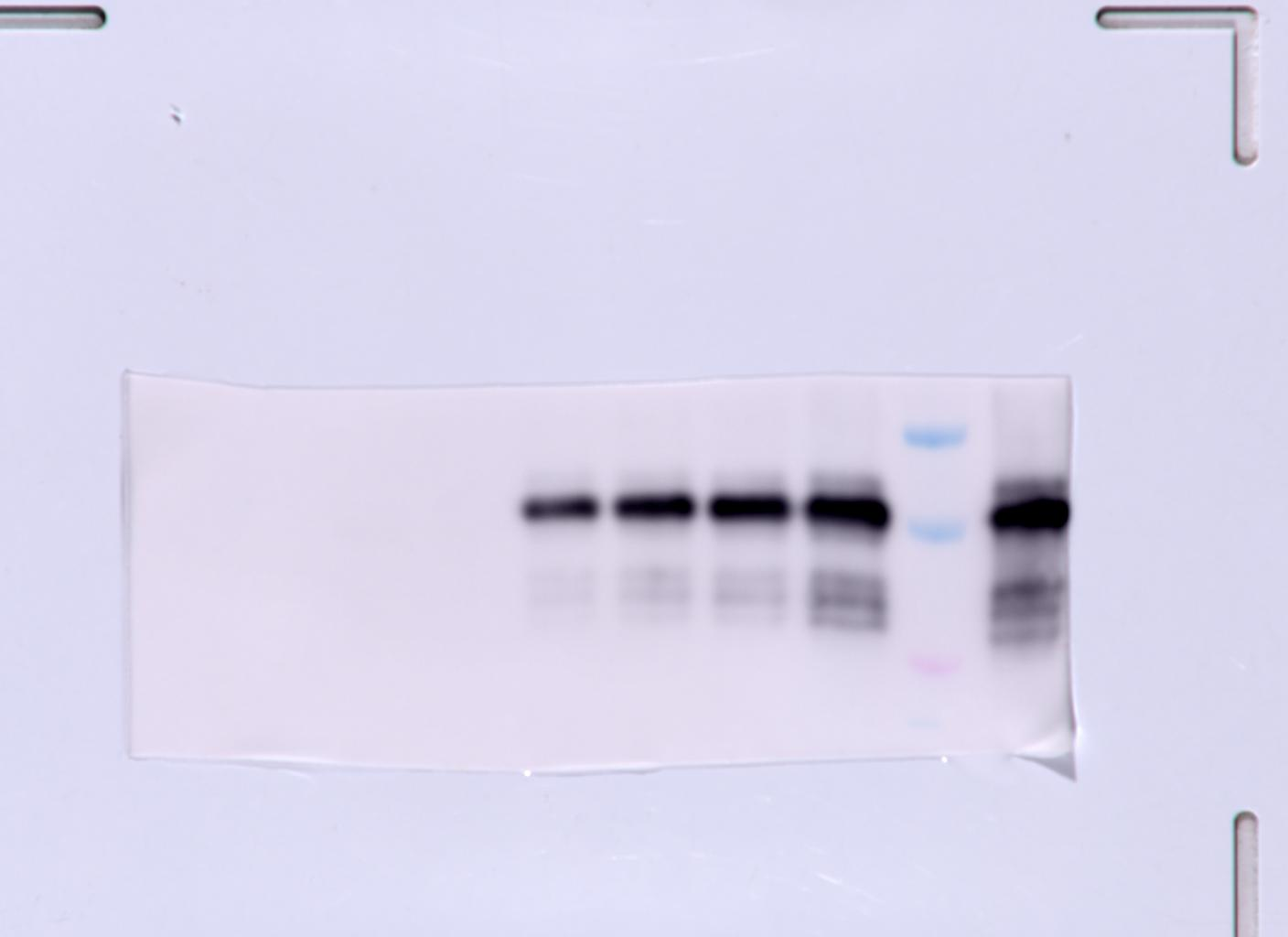

Supplement: Figure 5—source data 1. [file elife-98009-fig5-data1.zip › Figure 5-source data 1/Fig5E-pIkBa.tif]

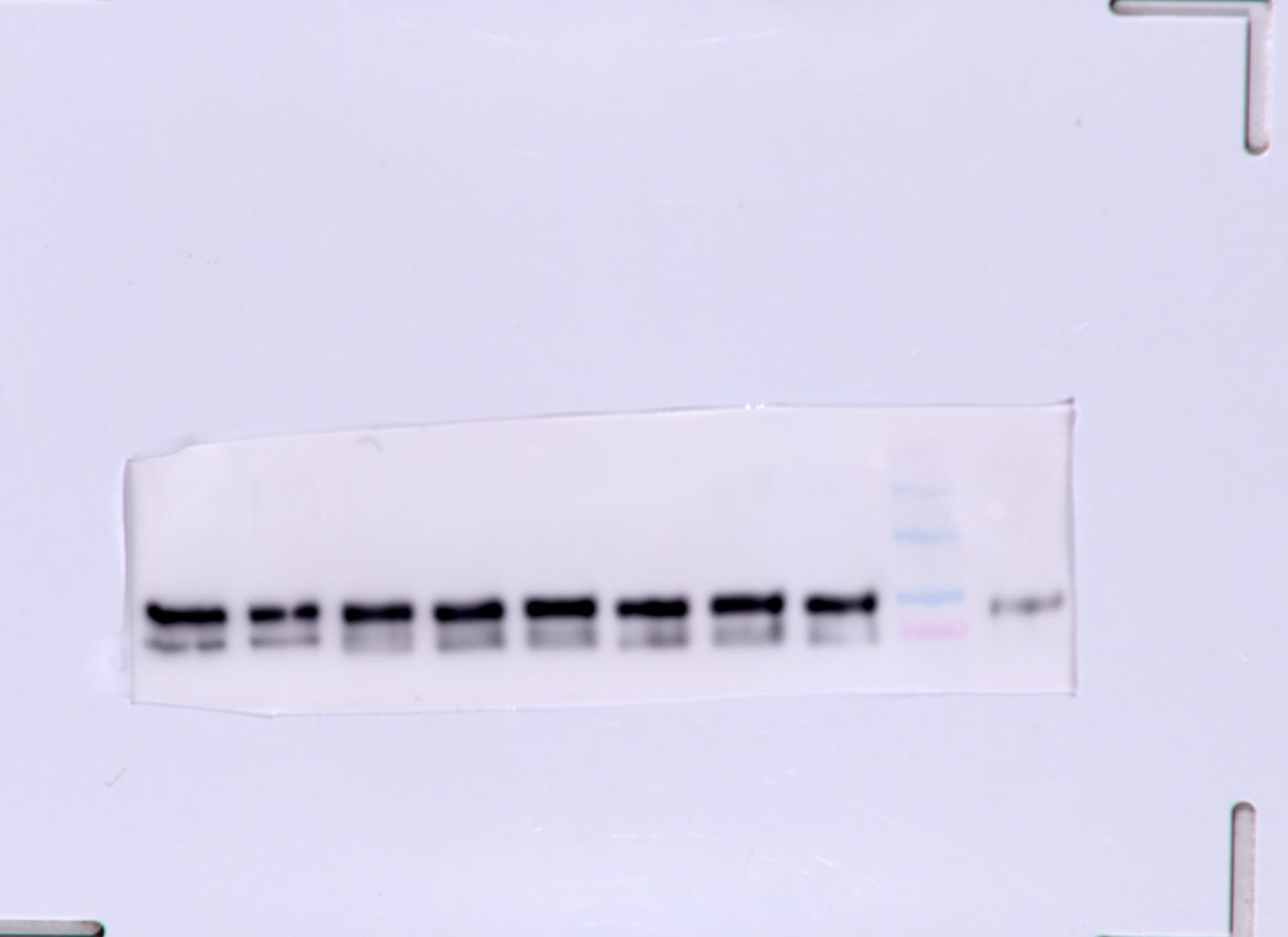

Supplement: Figure 5—source data 1. [file elife-98009-fig5-data1.zip › Figure 5-source data 1/Fig5E-IKK2.tif]

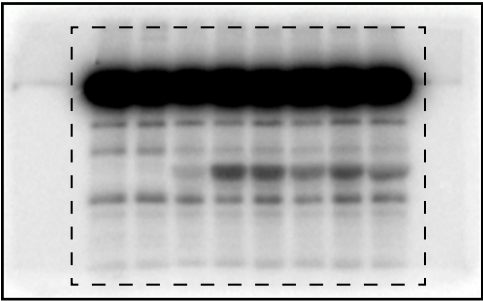

Autoradiograph

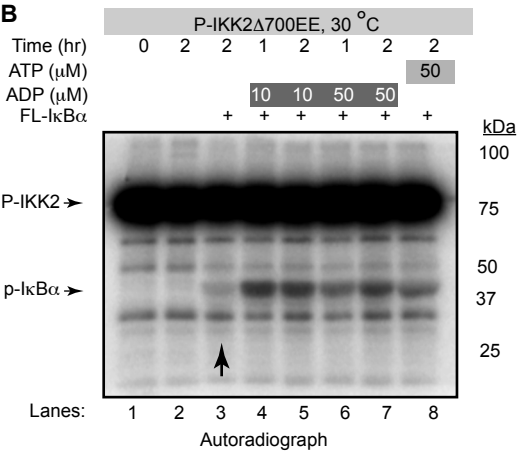

Supplement: Figure 5—source data 2. [file elife-98009-fig5-data2.zip › Figure 5-source data 2/Fig5B.pdf]

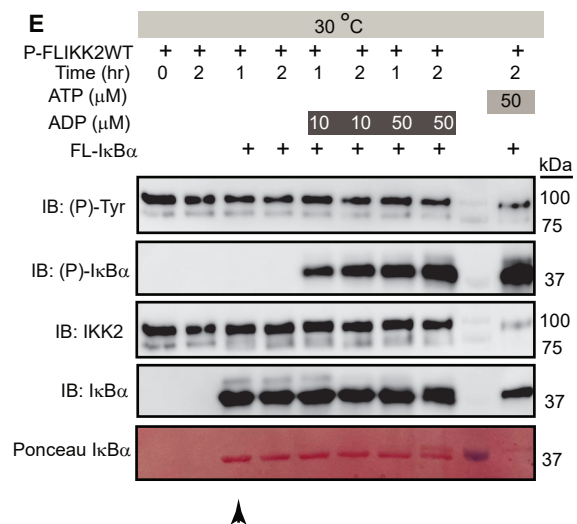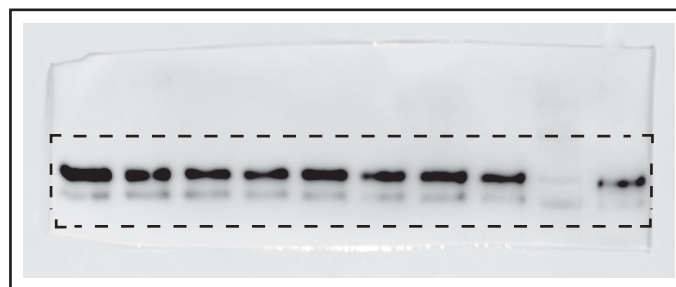

IB: (P)-Tyr

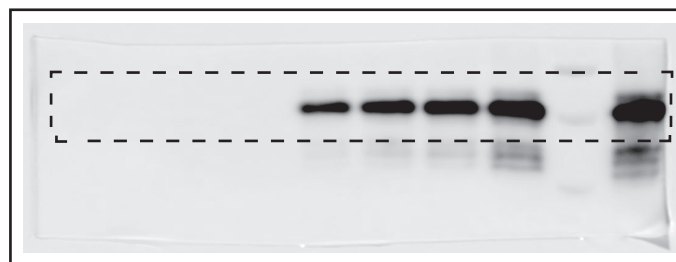

IB: (P)-IκBα

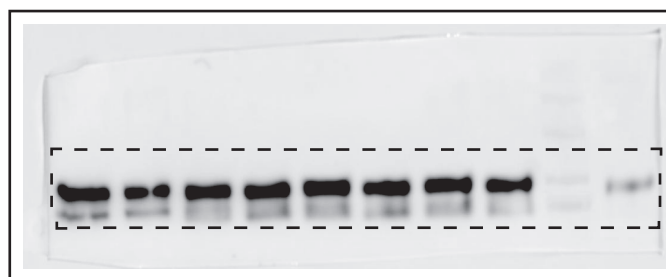

IB: IKK2

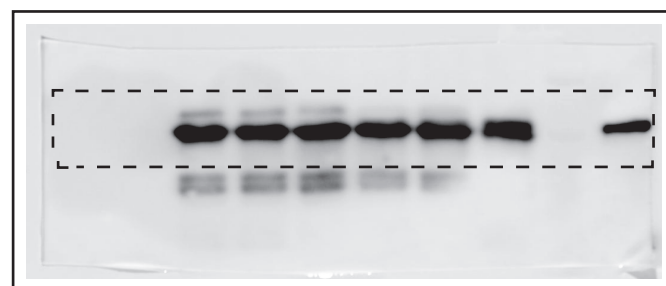

IB: IκBα

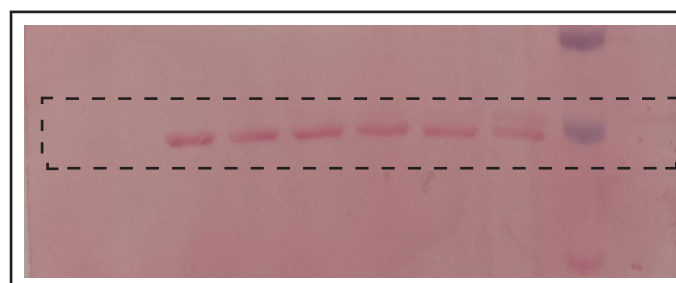

Ponceau IκBα

Supplement: Figure 5—source data 2. [file elife-98009-fig5-data2.zip › Figure 5-source data 2/Fig5E.pdf]

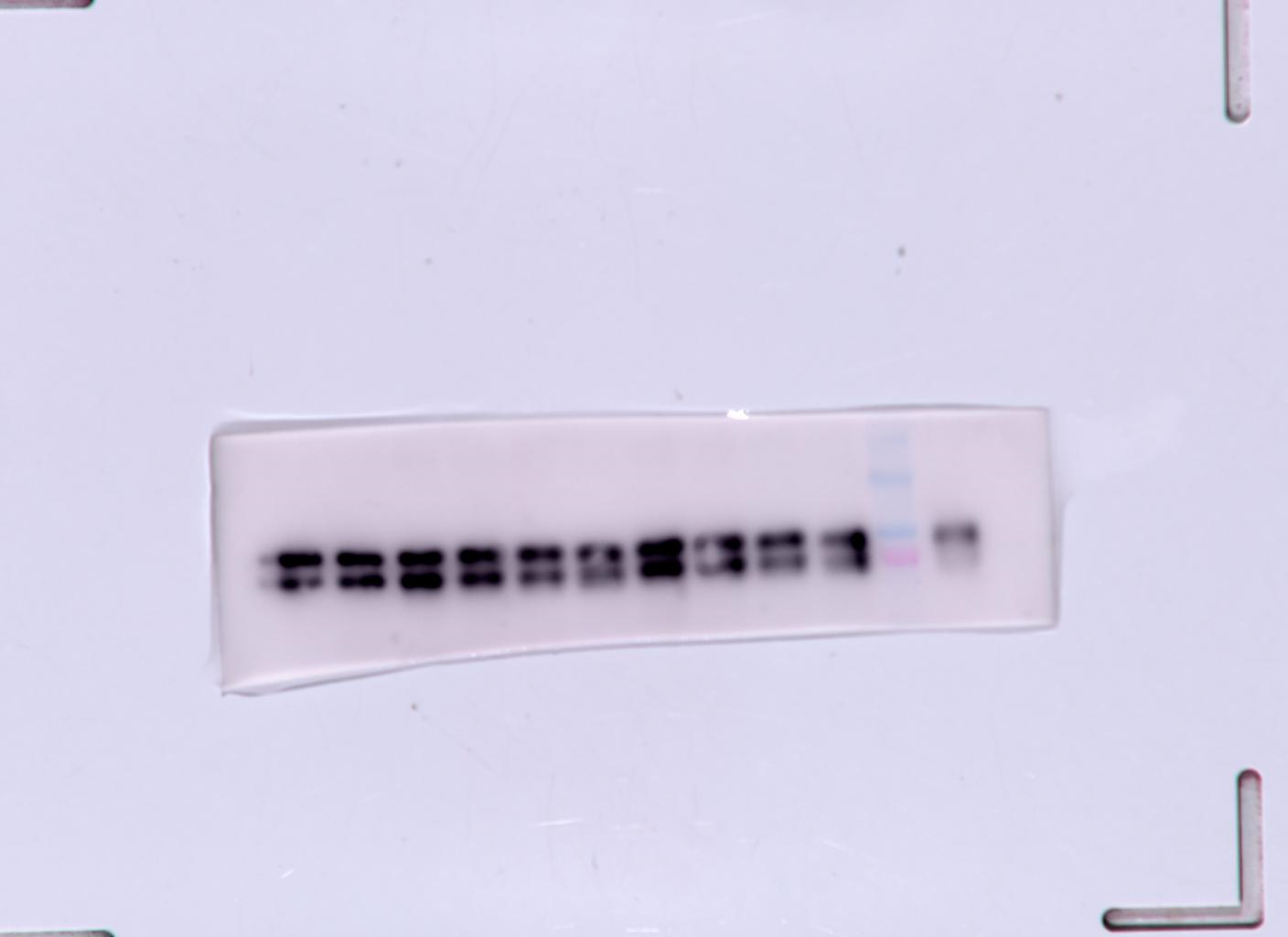

Supplement: Figure 5—figure supplement 1—source data 1. [file elife-98009-fig5-figsupp1-data1.zip › Figure 5-figure supplement 1-source data 1/Fig5-fig supp 1B-IKK2.tif]

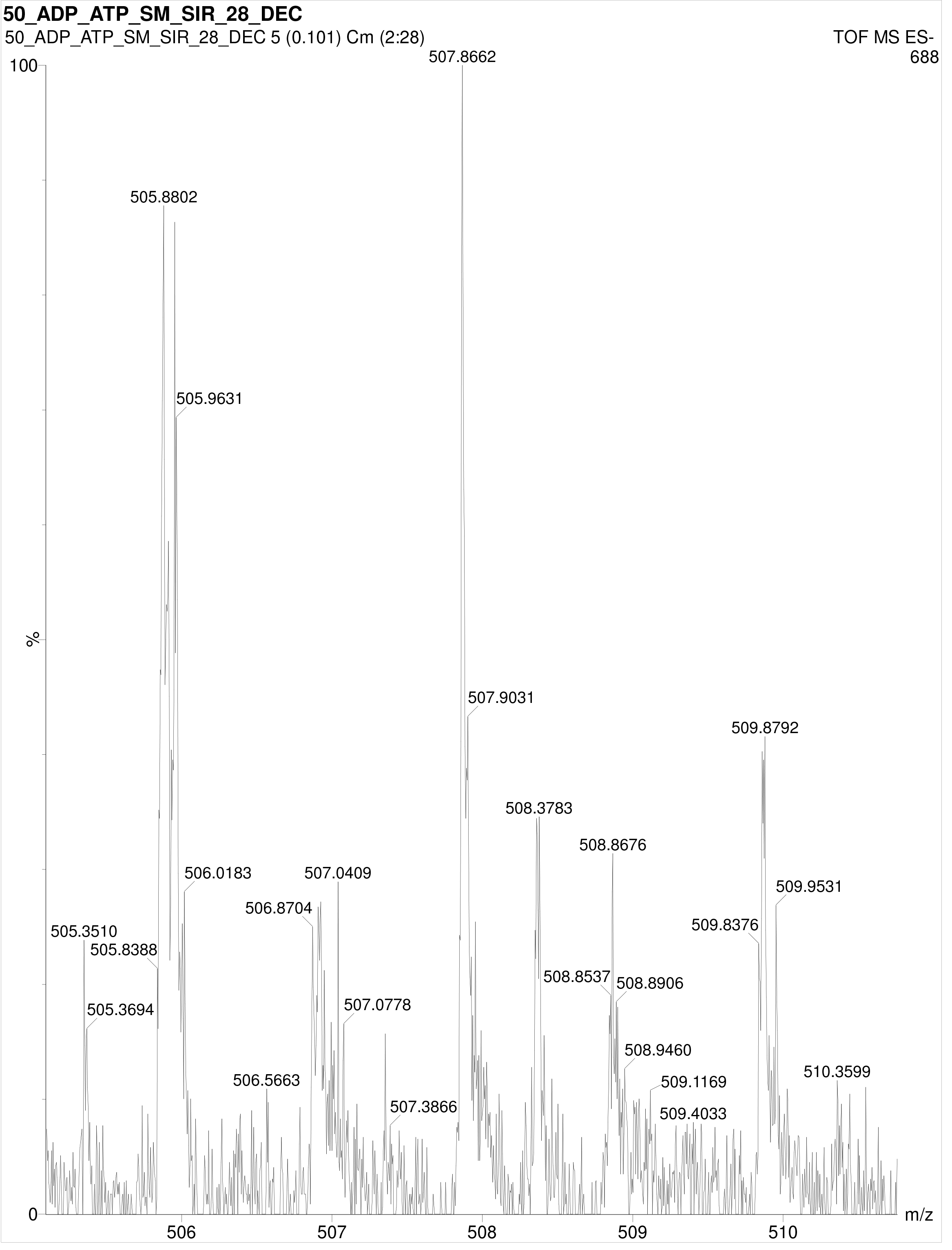

Supplement: Figure 5—figure supplement 1—source data 1. [file elife-98009-fig5-figsupp1-data1.zip › Figure 5-figure supplement 1-source data 1/Fig5-fig supp 1C-50_adp_atp_500-510.tif]

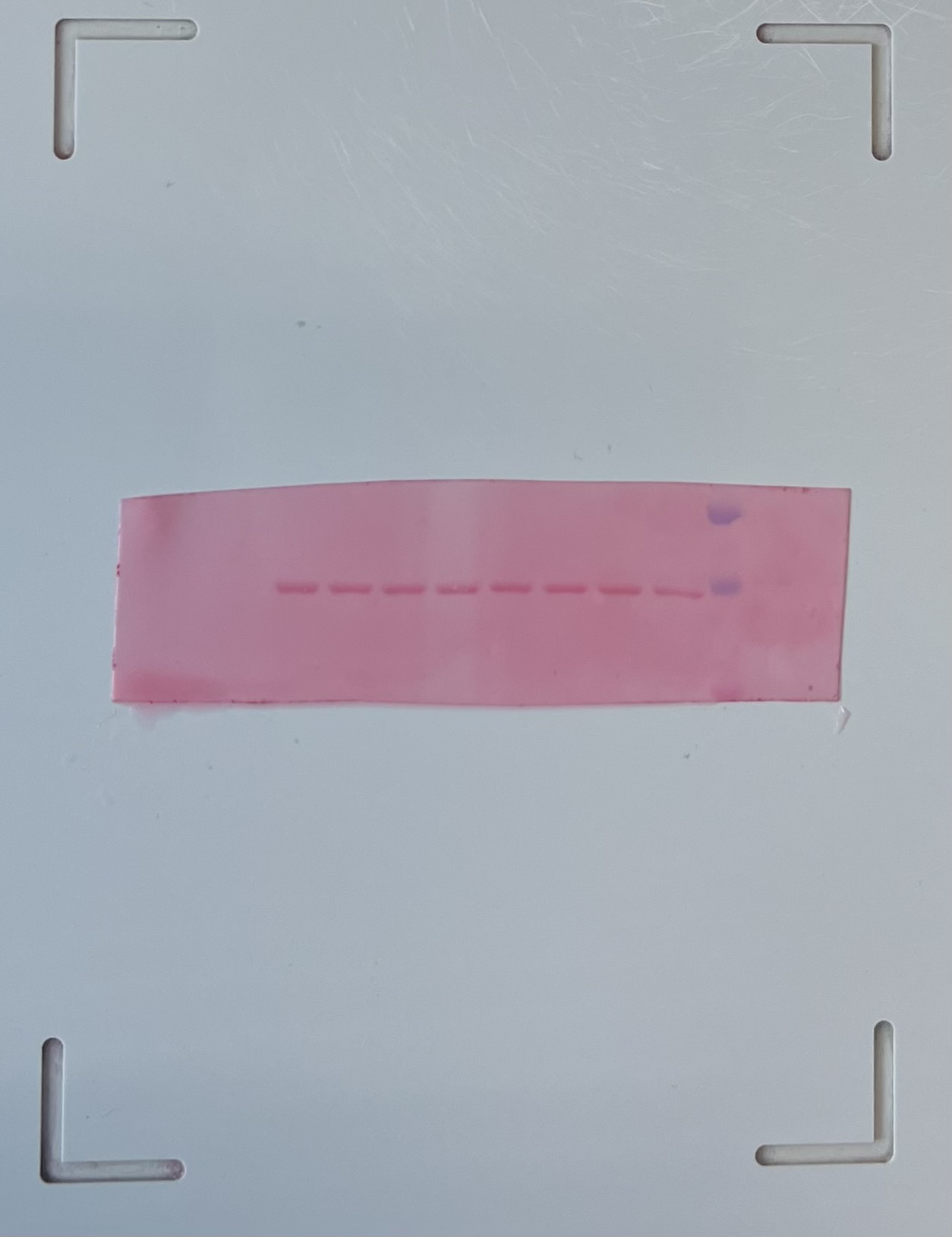

Supplement: Figure 5—figure supplement 1—source data 1. [file elife-98009-fig5-figsupp1-data1.zip › Figure 5-figure supplement 1-source data 1/Fig5-fig supp 1B-Ponceau.tif]

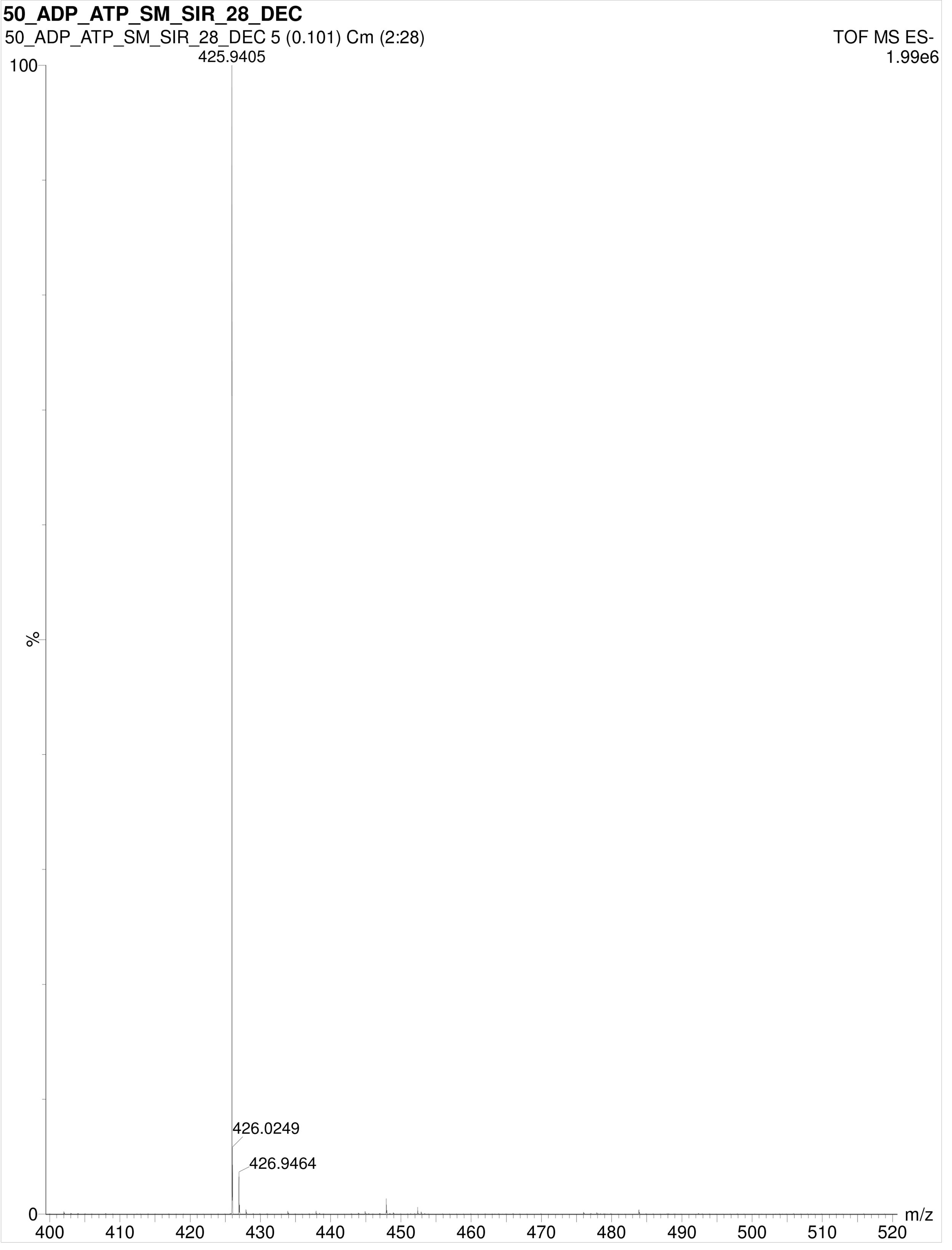

Supplement: Figure 5—figure supplement 1—source data 1. [file elife-98009-fig5-figsupp1-data1.zip › Figure 5-figure supplement 1-source data 1/Fig5-fig supp 1C-50_adp_atp_400-520.tif]

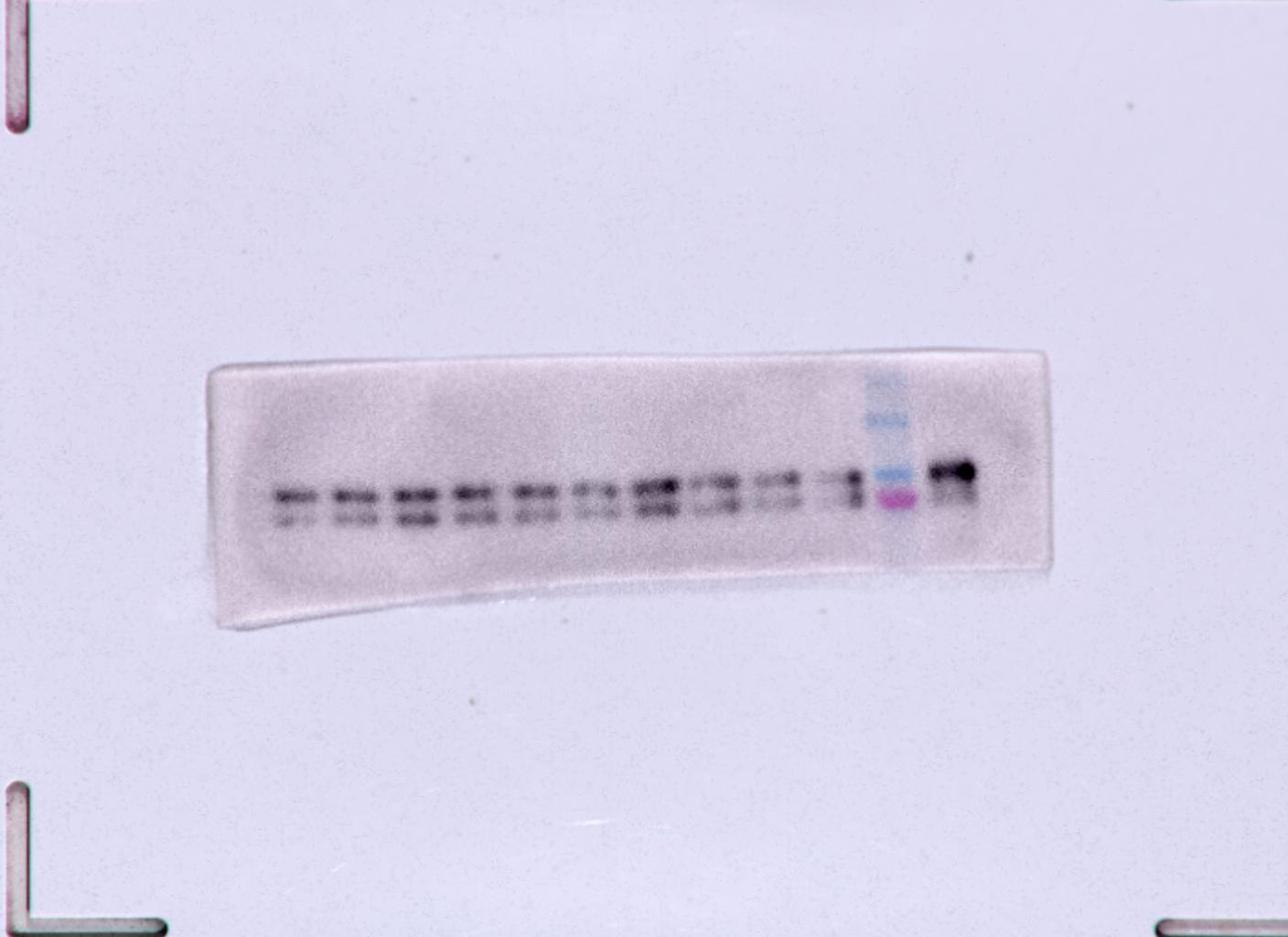

Supplement: Figure 5—figure supplement 1—source data 1. [file elife-98009-fig5-figsupp1-data1.zip › Figure 5-figure supplement 1-source data 1/Fig5-fig supp 1B-pTyr.tif]

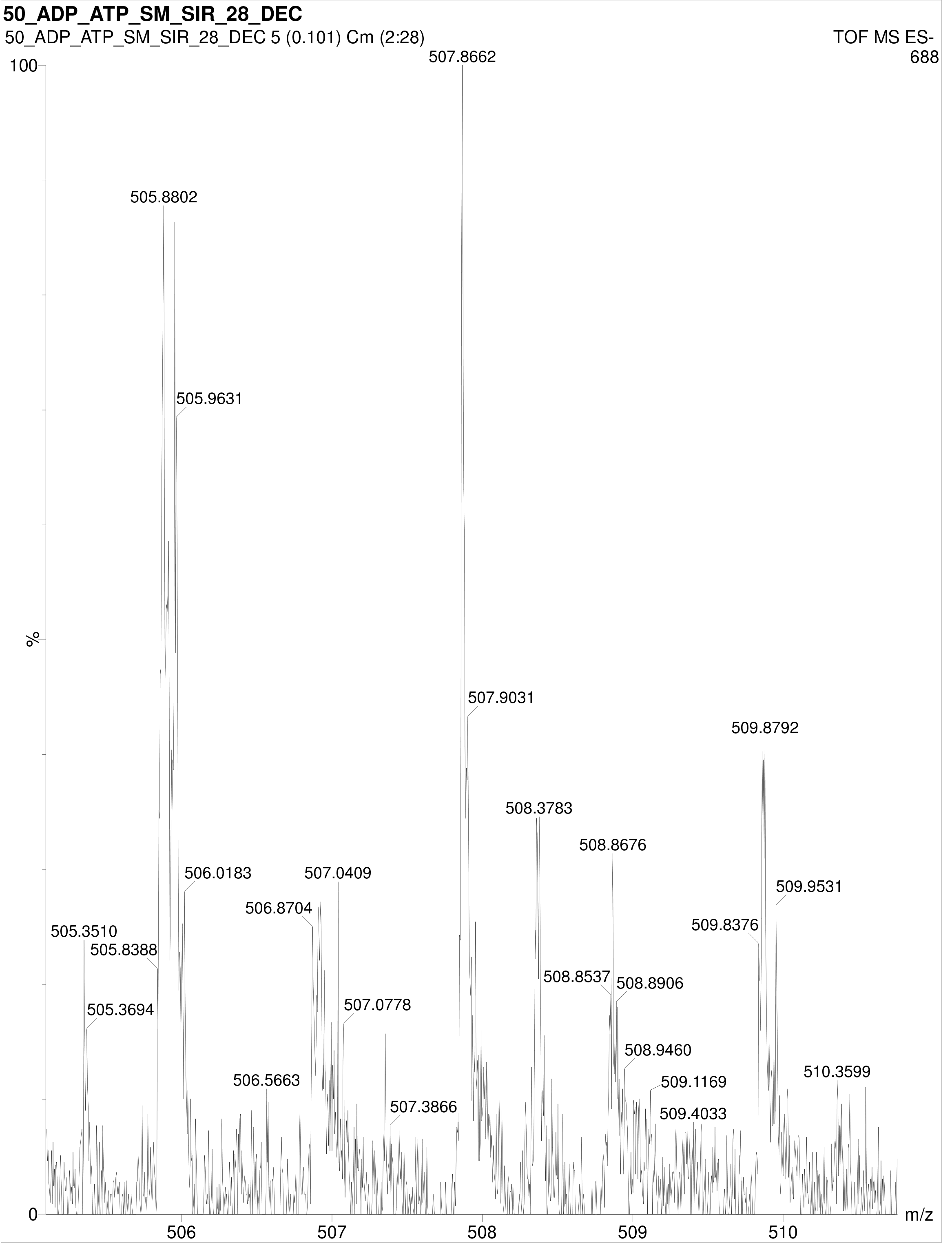

Supplement: Figure 5—figure supplement 1—source data 1. [file elife-98009-fig5-figsupp1-data1.zip › Figure 5-figure supplement 1-source data 1/Fig5-fig supp 1C-50_adp_atp_505-511.tif]

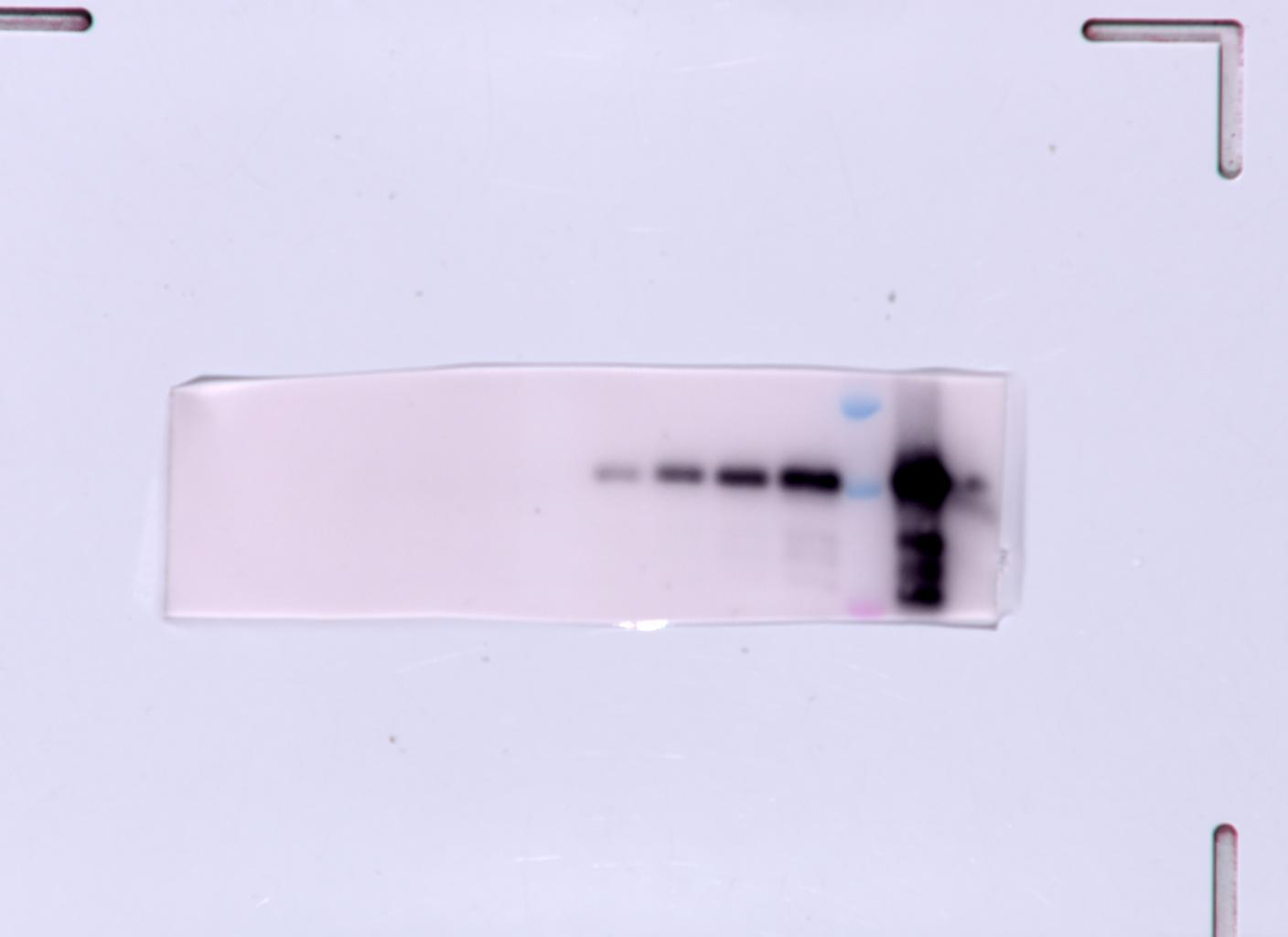

Supplement: Figure 5—figure supplement 1—source data 1. [file elife-98009-fig5-figsupp1-data1.zip › Figure 5-figure supplement 1-source data 1/Fig5-fig supp 1B-pikba.tif]
