# Supplementary figures and images for: Dual-specific autophosphorylation of kinase IKK2 enables phosphorylation of substrate IκBα through a phosphoenzyme intermediate (part 2 of 2)
Source: eLife. 2025 Jun 30;13:RP98009. doi: 10.7554/eLife.98009 (PMC12208667; doi:10.7554/eLife.98009)

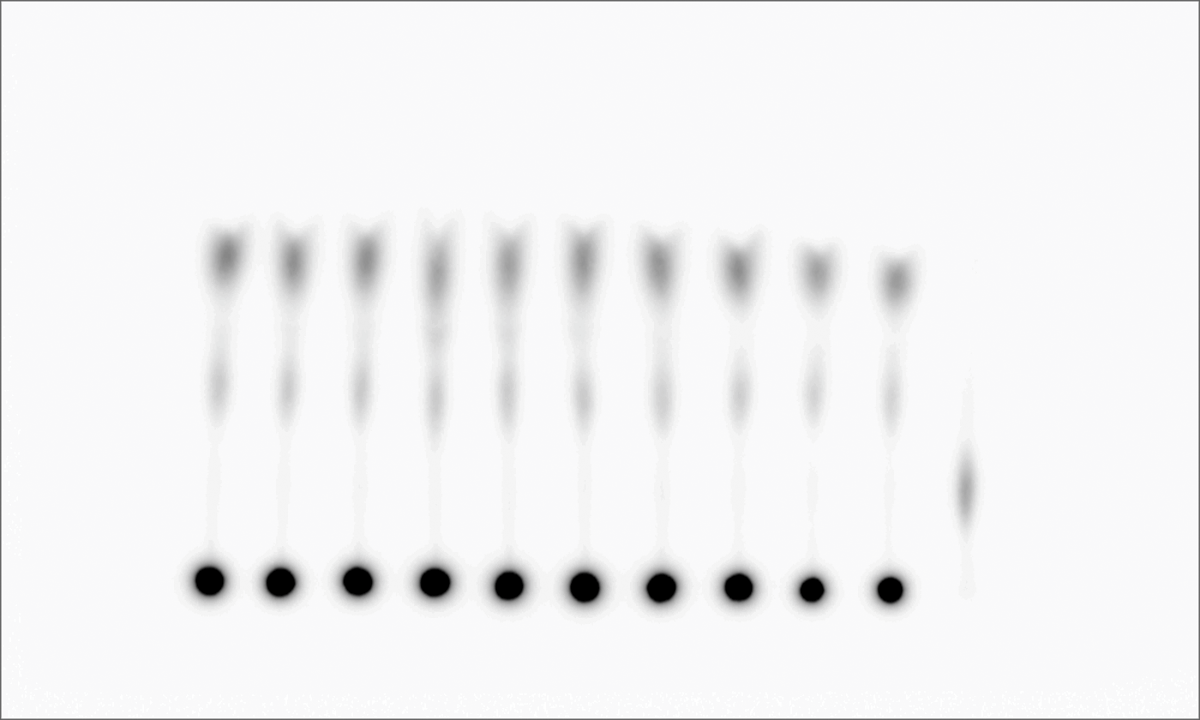

Supplement: Figure 5—figure supplement 1—source data 1. [file elife-98009-fig5-figsupp1-data1.zip › Figure 5-figure supplement 1-source data 1/Fig5-fig supp 1D-TLC-gray.tif]

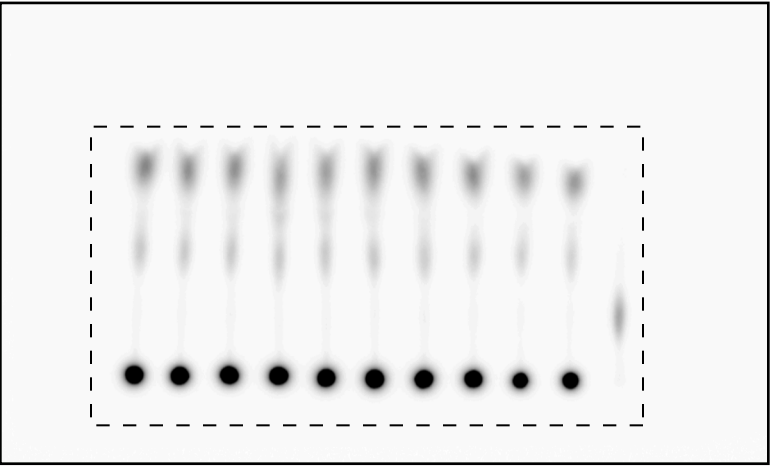

**D**

|                          |     |   |    |    |     |    |    |   |    |    |
|--------------------------|-----|---|----|----|-----|----|----|---|----|----|
|                          | 30C |   | RT |    | 30C |    | RT |   | RT |    |
| Time (mins):             | 30  | 5 | 15 | 30 | 5   | 15 | 30 | 0 | 30 | 30 |
| ADP (μM):                | 20  |   | 20 | 20 | 50  | 50 | 50 | 0 | 20 | 20 |
| P <sup>32</sup> -Δ700EE: | +   | + | +  | +  | +   | +  | +  | + | +  | +  |

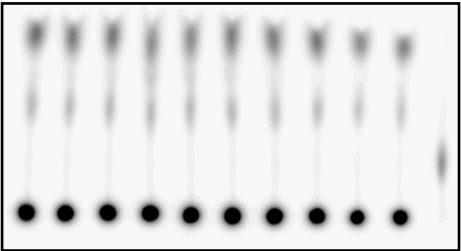

←ATP

PEI Cellulose TLC: Mobile Phase 0.5M K-Phosphate pH 3.5

Supplement: Figure 5—figure supplement 1—source data 2. [file elife-98009-fig5-figsupp1-data2.zip › Figure 5-figure supplement 1-source data 2/Fig5-figure supplement 1D.pdf]

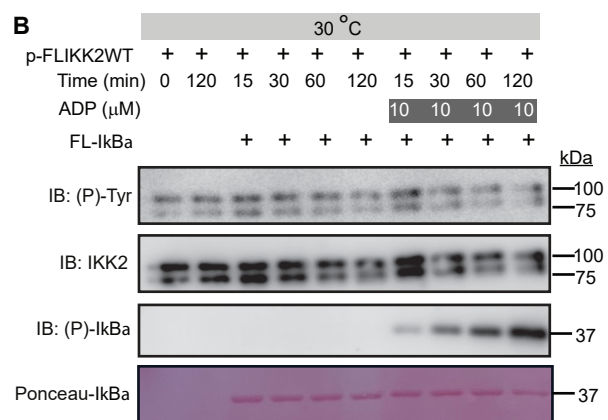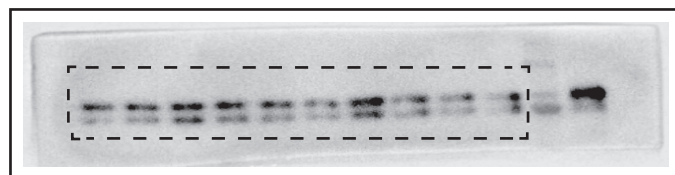

IB: (P)-Tyr

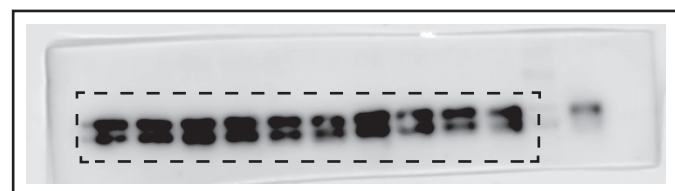

IB: IKK2

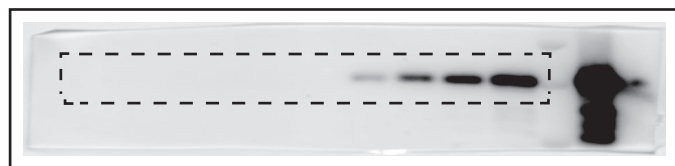

IB: (P)-IκBα

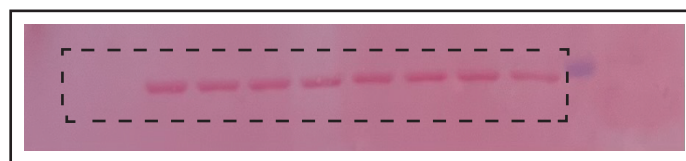

Ponceau-IκBα

Supplement: Figure 5—figure supplement 1—source data 2. [file elife-98009-fig5-figsupp1-data2.zip › Figure 5-figure supplement 1-source data 2/Fig5-figure supplement 1B.pdf]

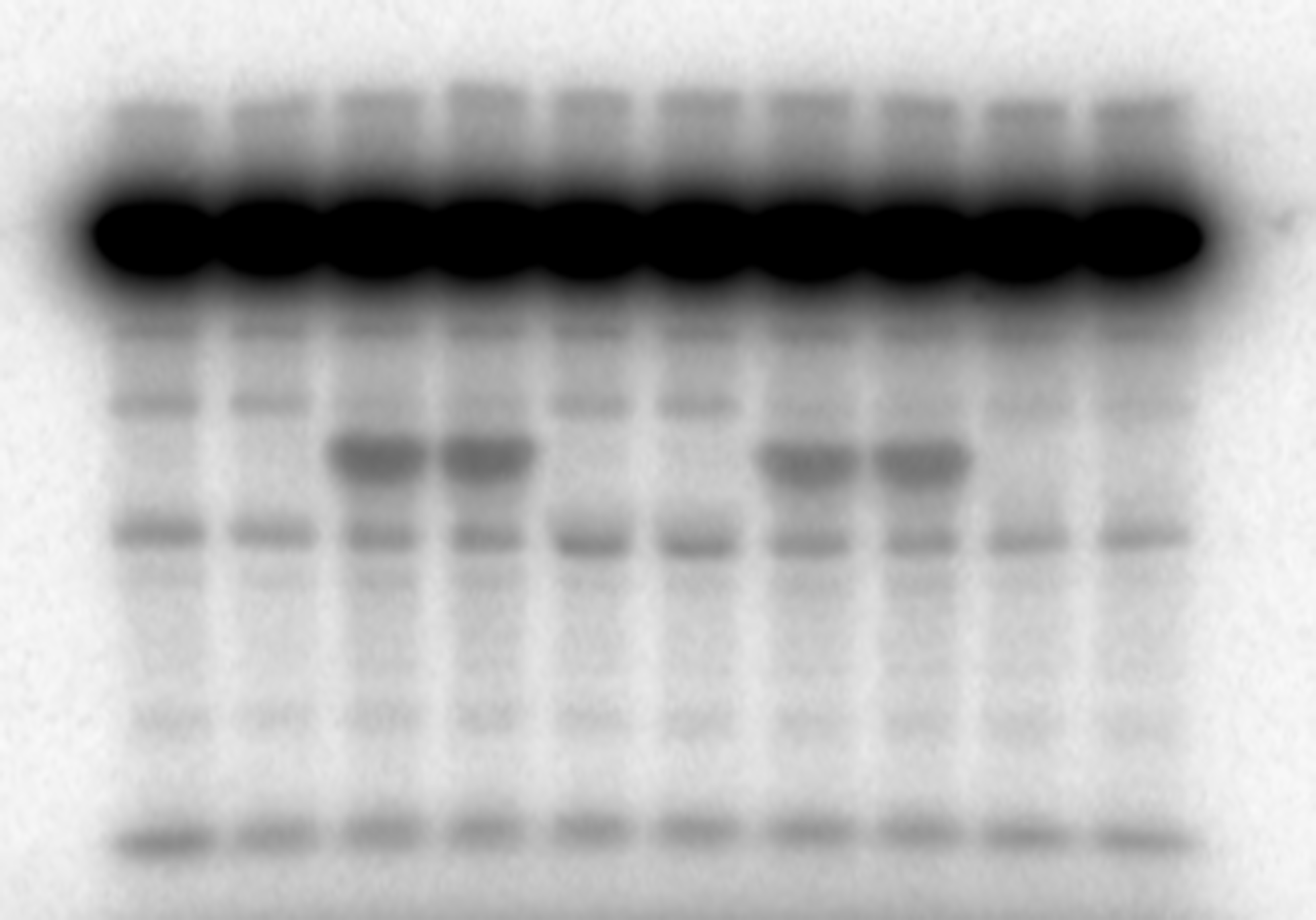

Supplement: Figure 6—source data 1. [file elife-98009-fig6-data1.zip › Figure 6-source data 1/Fig6A-autorad.tif]

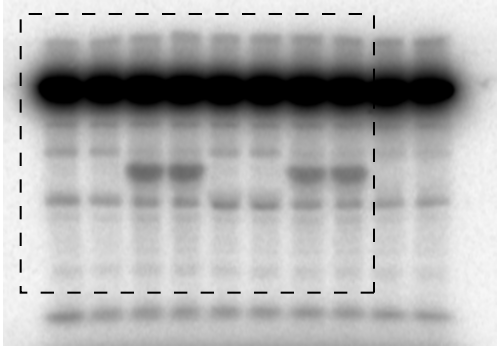

Autoradiograph

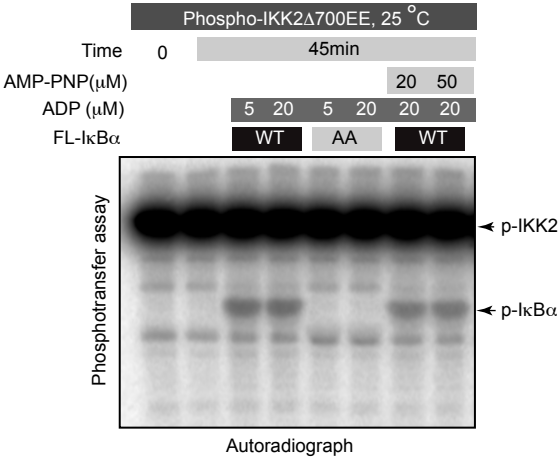

Supplement: Figure 6—source data 2. [file elife-98009-fig6-data2.zip › Figure 6-source data 2/Fig6.pdf]

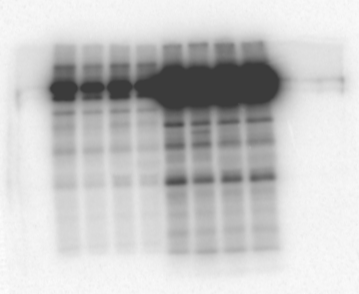

Supplement: Figure 6—figure supplement 1—source data 1. [file elife-98009-fig6-figsupp1-data1.zip › Figure 6-figure supplement 1-source data 1/Fig6-fig supp 1-autorad.tif]

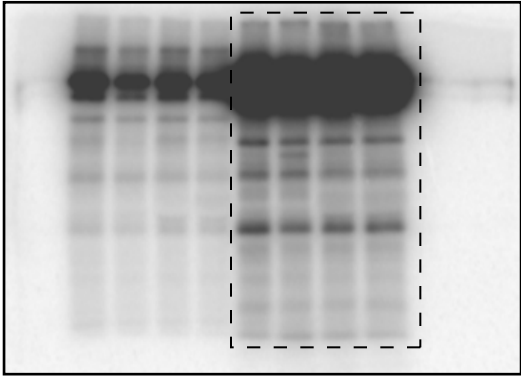

Autoradiograph

|              |       |     |     |     |
|--------------|-------|-----|-----|-----|
|              | 30 °C |     |     |     |
| Time (hr)    | 0     | 1.5 | 1.5 | 1.5 |
| p-IKK2 Y169F | +     | +   | +   | +   |
| ADP (μM)     |       |     | 20  |     |
| FL-IκBα WT   |       |     | +   | +   |

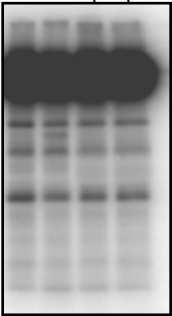

← p-IKK2  
Y169F

Autoradiograph

Supplement: Figure 6—figure supplement 1—source data 2. [file elife-98009-fig6-figsupp1-data2.zip › Figure 6-figure supplement 1-source data 2/Fig6-figure supplement 1.pdf]
