# Supplementary material for: Dual-specific autophosphorylation of kinase IKK2 enables phosphorylation of substrate IκBα through a phosphoenzyme intermediate
Source: eLife. 2025 Jun 30;13:RP98009. doi: 10.7554/eLife.98009 (PMC12208667; doi:10.7554/eLife.98009)
Supplement: Figure 3—source data 2. [file Fig3E and 3F.pdf › Figure 3-source data 2/Fig3E and 3F.pdf]

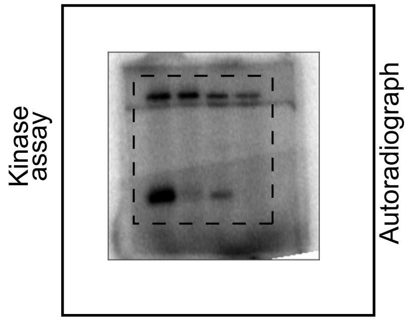

Kinase assay
Autoradiograph

E

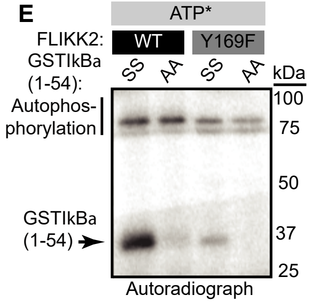

ATP*
FLIKK2:
WT
Y169F
GSTIkBa (1-54):
SS
AA
SS
AA
kDa
100
75
50
37
25
Autophos-phorylation
GSTIkBa (1-54)
Autoradiograph

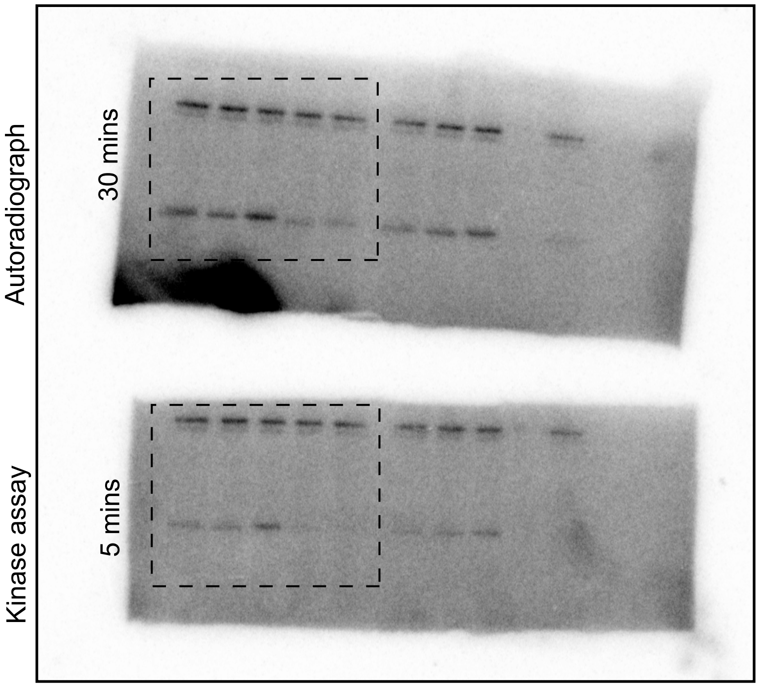

Autoradiograph
30 mins
Kinase assay
5 mins

F

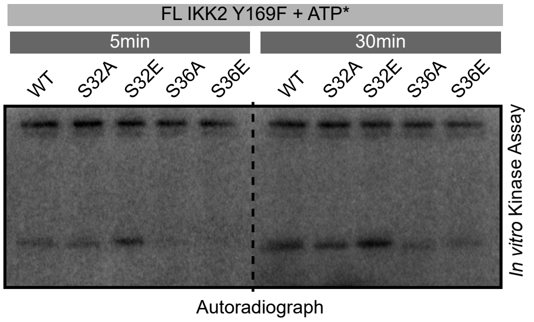

FL IKK2 Y169F + ATP*
5min
30min
WT
S32A
S32E
S36A
S36E
WT
S32A
S32E
S36A
S36E
In vitro Kinase Assay
Autoradiograph
